# Exploring potential phytocompounds from black cumin as drug molecules against SARS-CoV-2 infections through bioinformatics analysis

**DOI:** 10.1371/journal.pone.0337970

**Published:** 2026-03-11

**Authors:** Md. Ahad Ali, Humaira Sheikh, Md. Selim Reza, Tripti Rani Paul, Tasfia Noor, Neeraj Kumar, Mashooq Ahmad Bhat, Md. Nurul Haque Mollah

**Affiliations:** 1 Bioinformatics Lab, Department of Statistics, University of Rajshahi, Rajshahi, Bangladesh; 2 Department of Computational Chemistry and Drug Design, Panacea Research Center, Rajshahi, Bangladesh; 3 Department of Chemistry, Gopalganj Science and Technology University, Gopalganj, Bangladesh; 4 Department of Medicine, Henry Ford Health, Detroit, Michigan, United States of America; 5 Department of Pharmacy, School of Science and Technology, Varendra University, Rajshahi, Bangladesh; 6 Dept. of Computer Science and Engineering, Rajshahi University of Engineering & Technology (RUET), Rajshahi, Bangladesh; 7 Department of Pharmaceutical Chemistry, Bhupal Nobles’ College of Pharmacy, Udaipur, Rajasthan, India; 8 Department of Pharmaceutical Chemistry, College of Pharmacy, King Saud University, Riyadh, Saudi Arabia; Guru Nanak College, INDIA

## Abstract

SARS-CoV-2 was identified at the end of 2019 as the key cause of COVID-19, a global pandemic. As remedies, different vaccines as well as synthetic drugs have been recommended. However, the availability of natural drugs against SARS-CoV-2 infection is limited, although natural drugs are considered as less toxic than synthetic drugs, and vaccine efficacy is gradually weakened due to unstable RNA sequence patterns of SARS-CoV-2. Black-cumin is a well-known medicinal plant, but it was not rigorously investigated against SARS-CoV-2 infections. This study attempted to investigate this issue, rigorously. In order to explore effective bioactive phytocompounds from black-cumin (BC) through bioinformatics analysis, we selected top-ranked 11 drug target proteins/receptors of which five receptors were SARS-CoV-2 proteins/proteases (S, N, RdRp, 3CLpro, PLpro) and the other six receptors were host proteins (ACE2, MAPK8, TMPRSS2, IL6, TNF, and NFKBIA) associated with the infection by the systematic literature review. We computed binding affinity scores (BAS) for each phytochemical of BC with each of those 11 receptors. Top-ranked five phytocompounds (Silibinin, Taraxerol, Beta amyrin, Cycloartenol, and Alpha-sitosterol) were selected based on their highest average BAS across our proposed receptor, these phytocompounds also showed better binding capabilities against the other independent receptors. Then we selected top-ranked three complexes (ACE2 vs. silibinin, Spike vs. beta amyrin, and MAPK8 vs. Taraxerol) to investigate their binding stability using dynamic simulation (i.e., RMSD, RMSF, DCCM, PCA, and FEL) and MM-GBSA studies and found their stable performance. The ADMET, Bioactivity, and DFT analysis results supported the drug-likeness properties of the proposed phytocompounds. Therefore, the findings of this article might be useful resources for taking an alternative treatment plan against SARS-CoV-2 infections.

## 1. Introduction

The epidemic of severe acute respiratory syndrome coronavirus 2 (SARS-CoV-2) infection is a crucial risk factors for the ongoing socio*-*economic crisis worldwide. The SARS-CoV-2 was identified at the end of 2019 in Hubei Province of Wuhan, China, as the key cause of COVID-19 that is now a global pandemic. The SARS-CoV-2 has scatteredly spread worldwide due to the random mutation in its genome. It’s genetic mutations lead to the evolution of new variants that classified into variants of interest (VOI), variants of concern (VOCs), and variants being monitored (VBM) [1]. The VOIs like the Lambda (C.37) variant has showed potential increased transmissibility and immune escape [2], while VOCs such as Alpha (B.1.1.7), Beta (B.1.351), Delta (B.1.617.2), and Omicron (B.1.1.529) have demonstrated significant impacts on transmissibility and disease severity, necessitating adjustments in public health strategies [3]. As of 13 April 2024, SARS-CoV-2 infections were spread in 231 countries around the world with 704,753,890 infected cases in total in which 675,619,811 (96.05%) recovered, 7,010,681 (.998%) deaths and the rest 22,123,398 were actively infected. Among the actively infected cases, 22,088,604 (99.8%) were in mild condition and the rest 34,794 (0.2%) were in serious or critical condition [4].

The major protease Mpro/3ClPro, PLpro [5–7] and the RNA-dependent RNA polymerase (RdRp or also known as NSP12) [8,9] influence the COVID-19 by accelerating the replication of RNA from RNA template. These proteins are crucial for viral replication which make them potential drug targets for immunotherapy [6]. Also, previous studies exposed that SARS-CoV-2 infections are also stimulated by several host proteins including ACE2, TMPRSS2, IL6, TNF, NFKBIA and MAPK8 [10–21]. The host protein ACE2 (angiotensin-converting enzyme 2) is a primary receptor for viral entry into host cells, while TMPRSS2 (transmembrane serine protease 2) cleaves viral spike protein to activate membrane fusion and is co-expressed with ACE2 in lung/airway cells [22,23]. Thereby, dual inhibition of ACE2 and TMPRSS2 is a validated strategy to block cell entry. The core cytokines, IL-6 (Interleukin-6) and TNF (Tumor Necrosis Factor), drive the “cytokine storm” in severe COVID-19, leading to ARDS and multi-organ failure. These two proteins are clinically validated targets for inhibition; for example, IL-6 inhibitors (tocilizumab) and TNF blockers (infliximab) improve survival in critical patients. Both MAPK8 and NFkBIA are the key regulators for promoting viral replication, apoptosis, and inflammation. Therefore, researchers may consider viral protein and/or host-protein as drug targets [24–26].

Though most of the peoples around the world are already vaccinated by several vaccines (including, Pfizer, SII/COVISHEILD, Moderna, Janssen, Sinopharm, and Sinovac-CoronaVac), but COVID-19 is not yet fully controlled due vaccine efficacy is gradually weakened due to unstable RNA sequence patterns of SARS-CoV-2. Currently, different protease/proteins guided approved synthetic drugs **–** riboflavin [27], ritonavir/lopinavir [28], oseltamivir [29], minocycline [30], tocilizumab [31], corticosteroids [32], niclosamide [33], ciclesonide [34], and ribavirin [35] **–** are available for the treatment of SARS-CoV-2 infection. However these synthetic drugs might have some adverse effect on the immune system of human body, for example, hematologic abnormalities and flu-like symptoms [36]. The most common side effects of remdesivir using in the COVID-19 infections are respiratory failure and organ dysfunction, such as low albumin, low red blood cell count, low potassium, low platelet count, which helps clots, and yellow skin discoloration [37,38]. As well as other possible effects, including headache, nausea, low blood pressure (BP), sweating, vomiting and chills are reported in different literature [38,39]. Therefore, it is required to explore potential antiviral drug molecules against SARS-CoV-2 infections targeting both viral and host-guided proteins, since natural compounds are considered as less toxic than synthetic drugs [35]. *Nigella sativa* is a scientific name of Black cumin (BC) plant which is considered as a traditional medicinal plant due to its different therapeutics’ effects including, antibacterial, antiviral, anti-inflammatory, and anticancer activities [40–47]. The BC Seeds was also recommended as a medicinal source against SARS-CoV-2 infections in several studies [48–50]. Some studies have recommended some major phytocompounds derived from BC seeds, including nigellidine, nigellamine, alpha-hederin, thymoquinone, dithymoquinone (nigellone), thymol, carvacrol, and thymohydroquinone, as potential drug candidates against SARS-CoV-2 infections [22,46,47,51–61]. These studies did not analyze a complete list of BC phytocompounds during their investigation and considered only the seed and seed oil extracted major compounds, such as nigellidine, thymoquinone, dithymoquinone, and thymohydroquinone. Most of these studies considered only one major phytocompound of BC as the ligand during their investigation [47,53–55,58,62]. However, when researchers considered the larger library of BC phytocompounds, they found that some previously mentioned major compounds (e.g., thymoquinone, dithymoquinone, and thymohydroquinone) demonstrate the less potentiality than some minor compounds of BC [46,60,63]. Among them, a study considered at most 58 phytocompounds against the SARS-CoV-2 that suggested nigellidine, rutin, alpha-hederin as the candidate drug molecules based on their binding affinities. However, we found 300 phytocompounds of BC available in the database, but no researcher yet investigated all of these phytocompounds against SARS-CoV-2.

**Fig 1:**
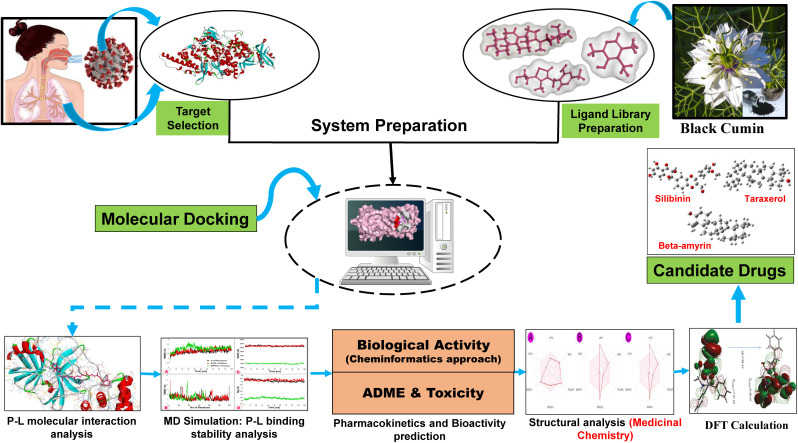
The graphical abstract and working flowcharts for this study.

On the other hand, two studies considered only host-proteins as the drug targets [55,56], and eight studies considered viral proteins as the drug target [47,51,52,57–61]. Few studies have considered both host and viral proteins as drug targets; but, they have overlooked several crucial proteins from both categories when exploring potential phytocompounds of BC as drug candidates [22,46,53,54]. It may be mentioned here that most of the antiviral drugs were developed by targeting only the viral proteins associated with virus replication and disease development [64–66]. However, the main problem with these antiviral drugs is that they often become resistant against the targeted viral proteins [67,68], since these proteins are often modified rapidly due to mutations during viral replication cycle. On the other hand, targeted host-proteins are stable compared to the targeted viral proteins (e.g., spike) [26,69]. It should be stated here that exploring candidate drugs against infectious diseases by targeting both host and viral proteins is better than exploring candidate drugs by targeting only viral or host proteins [70–72]. Therefore, in this *in-silico* study, an attempt was made to explore potential phytocompounds as the candidate drug molecules from the complete list of BC phytocompounds by targeting both viral and host key-proteins associated with SARS-CoV-2 infections. The full working procedure of this study illustrated in details in **Fig 1**.

## 2. Methods and materials

### 2.1. Data source and descriptions

In the case of drug discovery, the importance of the identification of target protein and therapeutic drugs/ligands are equal. In this study, we considered BC-phytochemicals as drug agents and SARS-CoV-2 infection-causing proteins/proteases as drug targets to explore potential BC-phytochemicals as the natural inhibitors against the infections. The detail descriptions about data source and their collection are given in subsections 2.1.1 and 2.1.2.

#### 2.1.1. Target protein selection & preparation.

A total of 100 published articles was reviewed associated with SARS-CoV-2 infections to find the infections causing key genes (KGs) or, hub-genes (HGs) using different key words from several database, for example Google Scholar, PubMed, etc. From these studies, we found total of 158 KGs published in the reviewed articles (S1 Table). After that, we selected top-ranked 11 SARS-CoV-2 infections causing proteins/proteases as drug targets based on their highest frequency on the reviewed articles (S2 Table), of which five receptors were SARS-CoV-2 proteins/proteases and the other six receptors were infection-causing host proteins for exploring potential phytocompounds from black-cumin by molecular docking analysis.

The crystalized 3D structures of these target proteins (ACE2, NSP12/RdRp, 3CLpro, S, TMPRSS2, PLpro, IL6, TNF, N, NFKBIA, MAPK8) were downloaded from the Uniport and RCSB protein data bank [73] by using their PDB ID’s 2ajf, 7bv2, 6lu7, 7t9k, 7meq, 6w9c, 1alu, 1a8m, 6m3m, 6y1j, and 4hyu, respectively. After extracting the proteins from the PDB database, the PyMOL software V2.0 [74] was used to visualize the sequences of those proteins and imputed the missing residues in the target proteins. All the heteroatoms, and co-crystallized ligands were removed from the PDB file by using the BIOVIA Discovery Studio program 2021 [75]. Then, the structures of the proteins were minimized by using Swiss-PDB Viewer *v*4.1.0 [76].

#### 2.1.2. Collection of Black Cumin’s phytochemicals.

In this study, we considered 300 phytochemicals of BC as the natural ligands that were available in the IMPPAT database [77] (*accessed on March 2025*). Finally, we select 241 unique phytocompounds out of 300, based on the duplication of their entry number and found 59 phytocompounds IMPPAT_ID duplication in the database. The 3D structures of the selected 241 phytocompounds were downloaded using PubChem database [78]. The universal force field (UFF) was used to minimized the energy of the phytocompounds in Pyrx 0.8v tools and the selected SDF files were converted into Autodock ligands as PDBQT format by using Open Babel [79] in PyRx 0.8v [80].

### 2.2. Screening of Phytochemical by Lipinski’s rule of 5

The druglikeness properties of the selected phytocompounds were investigated according to Lipinski’s rule of 5 (LOR5) [81] and Veber rule (VR). According to LOR5 the molecular weight should not be greater than 500Da, the hydrogen bond donor atom should be ≤ 5, the acceptor bond should be ≤ 10 and the lipophilicity index or LogP value should be ≤ 5 [82]. Whereas, in VR the rotatable bond should be ≤ 10 and the polar surface area (PSA/TPSA) should be equal to 140 or less [83]. The drug-likeness properties were investigated by using the web-based server ADMETlab 2.0 [84] and SwissADME [85].

### 2.3. Screening of phytochemical by molecular docking

To explore black cumin phytochemicals as the candidate drug molecules/ligands by molecular docking, we considered top-ranked hub-genes (HGs) as the target receptors. Molecular docking between receptors and ligands were conducted by using the Autodock-Vina [86] plugin in PyRx 0.8v tools [80] to calculate the BAS in kcal/mol of each pose and their non-bonding interactions. We ordered the receptors and phytocompounds in the matrix based on their average BAS to select the top-ranked phytocompounds as the candidate drug molecules. Then, we considered the negatively highest scorer complexes with the top-ranked phytocompounds for further analysis. The molecular interaction of the protein-ligand (P-L) complexes were investigated by using the BIOVIA Discovery Studio program 2021 [75]. All the information regarding docking analysis including the target receptors resolution, PDB id, their native ligand, grid box coordinates were showed in S3 and S4 Table.

### 2.4. MD Simulation Studies with top ranked drug-target complexes

Molecular dynamics is the study of the dynamic behavior of P-L complexes in a simulated physiological environment [87]. Herein, the molecular dynamics simulation was performed using the ‘desmond v2020-4 software’ in Schrödinger (Academic version) in Linux environment. The P-L complex system was solvated under the SPC (simple point charge) solvent system. To neutralized and emulates a biological system we construct a 10 × 10 × 10 Å shaped orthorhombic boundary box with a salt concentration of Na+ and Cl− ions were 0.15 M, assigned to both sides of the box. Furthermore, the OPLS3e force filed were used to stabilized the system. Additionally, the system was operated at a temperature of 300 K and a pressure of 1 atm. The MD investigation was then carried out for two femtosecond time steps with the NVT and NPT ensembles, which ultimately resulted in 5000 frames, with a duration of 200 nanoseconds. The stability and flexibility of the P-L system were then studied from the plot of the RMSD, RMSF, SASA, RoG, and H-Bonds calculation form the trajectories analysis.

#### 2.4.1. Root Mean Square Deviation (RMSD) analysis.

Th PL complexes stability can be predicted by using RMSD calculation. The RMSD results were plotted by using the following statistical equations:


$$RMSD=\sqrt{\frac{\text{1}}{N}}\sum_{i=\text{1}}^{n}{\left(r_{i}(t_{x})-r_{i}^{\prime }\;(t_{ref})\right)}^{2}$$

Where, N = selected atom number, t_ref_ = the reference time, t_x_ = the length of the recording intervals, r = the location of the particular atom in frame x after alignment with the reference frame

#### 2.4.2. Root Mean Square Fluctuation (RMSF) analysis.

RMSF measures the local conformational change within the residues of the protein structure. The RMSF value of a protein can be calculated by the given equation.


$$RMSF=\sqrt{\frac{\text{1}}{T}}\sum_{t=\text{1}}^{n}{\left(r_{i}(t)-r_{i}^{\prime }\;(t_{ref})\right)}^{2}$$

Where, T = calculated time from each trajectory

#### 2.4.3. MM-GBSA binding energy calculation.

Finally, the P-L binding free energy (BFE) for each snapshot was calculated using the gmx_MMPBSA tool. To explore the biophysical underpinnings of PL interactions, the Molecular Mechanics–Generalized Born Surface Area (MM-GBSA) approach was employed for the top-ranked three ligands and a standard compound. MM-GBSA calculations were utilized to estimate the binding affinities of the PL complexes.

The following equation was utilized for the BFE (Δ*G*_*bind*_) calculation [88]:


$$\Delta G_{bind}=\langle \text{GPL}\rangle -\langle \text{GP}\rangle -\langle \text{GL}\rangle$$

where ΔG_bind is the BFE, ⟨G_PL⟩ is the average free energy of the P-L complex, ⟨G_P⟩ is the free energy of the unbound protein, and ⟨G_L⟩ is the free energy of the unbound ligand.

Equation can be represented as:


$$\Delta G_{bind}=\Delta E_{MM}+{\Delta G}_{SOLV}-T\Delta S$$

where ΔE_MM_ represents the gas-phase molecular mechanics energy (including van der Waals and electrostatic interactions), is the solvation free energy change, and TΔS denotes the entropic contribution.

### 2.5. Pharmacokinetics (ADME/T) analysis

We performed *in-silico* validation for the selected phytocompounds *(< n*) by investigating their drug-likeness, ADME and Toxicity properties as discussed below:

The top-ranked phytocompounds were taken for their *in-silico* validation through the investigation of drug likeness, ADME (Absorption, Distribution, Metabolism, and Excretion) and toxicity or ADME/T properties. We considered three different web-tools SwissADME [85], pkCSM [89] and ProTox [90] for drug likeness and ADME & Toxicity analysis, since none of these tools support all computational results of our interest. The drug-likeness and bioavailability properties were investigated by using the web-tool SwissADME [85]. ADME analysis was performed through the web-tool pkCSM [89] and toxicity analysis through pkCSM & ProTox [90]. We used isomeric SMILE file format for all phytocompounds from PubChem database [78] as input file in all validation as mentioned above.

### 2.6. Density Functional Theory (DFT) analysis

We considered some top-ranked phytocompounds to study their structural and functional properties and their interactions with the receptors by using density functional theory (DFT) analysis which is a quantum mechanics-based model. It is widely used to calculate energies of molecular orbital (MO), energy gap (∆E), electronic properties (such as, electrophilicity index, electron affinity, softness, and hardness), and thermodynamic energies in the ground state of the proposed compounds by using the basis-set 6-311G [91] with Becke-3 Parameter-Lee-Yang-Parr (B3LYP) [92] method on Gaussian-09 software programs [93]. These properties are essential to understand the magnitude of ligand interaction with the receptor binding pocket [94,95]. The output data including the optimized geometry, dipole moment, HOMO-LUMO and other properties were visualized by using the GaussView-6 graphical interface [96].

## 3. Results

### 3.1. screening based on physicochemical properties (LOR5)

In this study, the canonical SMILE of 241 Phytocompounds were retrieved from the database and the physicochemical properties of these phytocompounds were calculated form SwissADME web server [85]. To determine the druglikeness properties of the selected compounds Lipinski’s rule of 5(LOR5) and Veber rule (VR) is mostly considered. The molecular wight (MW), lipophilicity index or LogP, number of hydrogen bond donor (nHBD) and acceptors (nHBA), polar surface area (PSA), rotatable bond (RotB), and Bioavailability score are considered in this study, and we found that 206 compounds were passed by both VR and LOR5 (S5 Table). **Table 1** represent the physicochemical properties of the selected phytocompounds that shows highest affinity to the target proteins of SARS CoV-2 infection.

**Table 1:** Physicochemical and Drug-likeness properties of the selected top-ranked 10 phytocompounds.

Phytocompounds	MW (Da)	LogP	nHBA	nHBD	PSA	RotB	Veber	Lipinski’s
Silibinin/Silymarin	482.12	2.21	10	5	155.14	4	Good	Pass
beta-Amyrin	426.39	7.713	1	1	20.23	0	Good	Pass
Taraxerol	426.39	6.782	1	1	20.23	0	Good	Pass
24-Methylenelophenol	412.37	7.529	1	1	20.23	5	Good	Pass
5-Dehydro-avenasterol	410.35	6.492	1	1	20.23	5	Good	Pass
alpha1-Sitosterol	426.39	7.558	1	1	20.23	5	Good	Pass
alpha-Spinasterol	412.37	7.492	1	1	20.23	5	Good	Pass
Betulinic acid	456.36	5.867	3	2	57.53	2	Good	Pass
Iso-avenasterol	412.37	7.247	1	1	20.23	5	Good	Pass
Butyrospermol	426.39	8.445	1	1	20.23	4	Good	Pass

### 3.2. screening based on binding affinity by molecular docking

**Fig 2:**
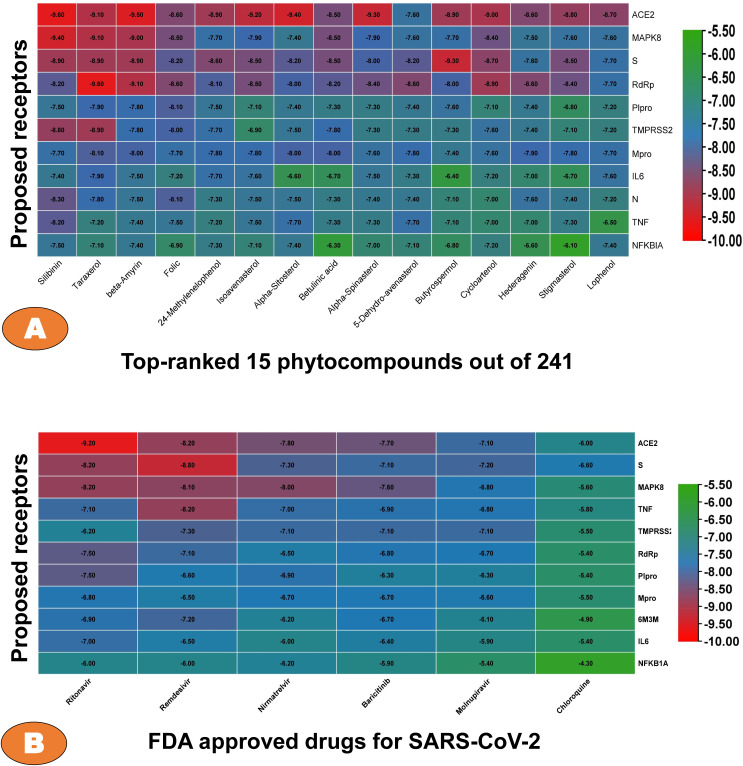
(A) The score matrix based on binding affinity scores (BAS) of the selected top-ranked 30 phytochemicals out of 241 against the proposed 11 receptor-proteins; (B) The docking score of FDA approved drugs as reference drug against SARS-CoV-2 infection vs proposed receptors, where blue color indicates our top-ranked proteins.

To explore potential phytocompounds of BC seed as the drug molecules against COVID-19, we performed pairwise P-L docking analysis among the top-ranked 11 target proteins and 241 phytocompounds (S6 Table). Then we displayed the top-ranked 15 phytocompounds vs. 11 target proteins binding affinity score matrix with a threshold value of −7.5 kcal/mol *(***Fig 2A**). Among them, the top-ranked 5 phytocompounds, Silibinin, Taraxerol, Beta amyrin, Folic Acid, and 24-Methylenelophenol, were selected as the candidate drug molecules based on their highest average binding affinity (∆G) with −9.60, −9.10, −8.90, −8.60, and −7.50 kcal/mol, respectively. These are considered as the best binding poses of these compounds to their corresponding target proteins.

Now, to compare the proposed phytocompounds with some standard/reference drugs (RDs) of SARS-CoV-2 infections by docking analysis, we considered 6 FDA approved drugs (Ritonavir, Remdesivir, Nirmatrelvir, Baricitinib, Molnupiravir, and Chloroquine) from world health organization’s web portal [97]. It was observed that the Ritonavir produces highest average docking score of −7.3 kcal/mol across proposed receptors compare to the other reference drugs (**Fig 2B**). Therefore, the proposed phytocompounds shows strong binding score compare to the RDs against the proposed receptors.

#### 3.2.1. Molecular interactions of the top-ranked complexes.

**Fig 3:**
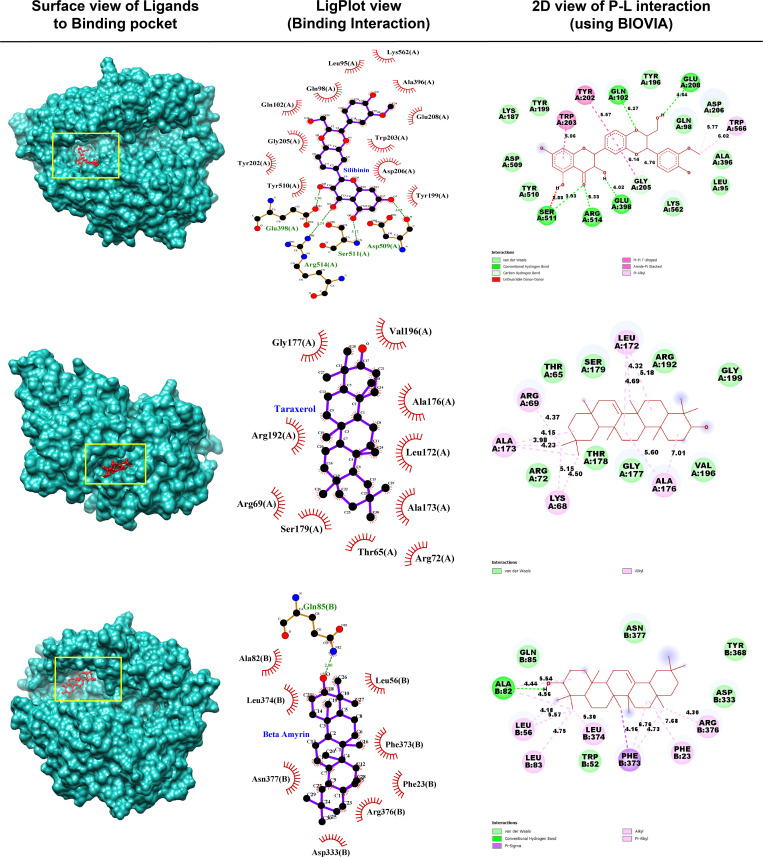
Displaying the surface view, pose view and amino acid residues for the protein-ligand interactions, where (A) ACE2 *vs* Silibinin complex, (B) MAPK8 *vs* Taraxerol complex, and (C) S *vs* Beta amyrin complex.

The selected top 5 phytocompounds were considered for further validation by molecular interaction with their corresponding target protein. It was observed that, only Silibinin, Taraxerol and Beta amyrin shows strong affinity toward their target protein ACE2, MAPK8, and S with their highest BAS of −9.6, −9.1 and −8.9 kcal/mol respectively. High binding affinity is primarily accompanied by H-bonding, which is an important indicator of strong P-L interaction. The amount of hydrogen bonds in a P-L interaction usually increases the inhibitor’s activity against the target protein. As shown in **Fig 3**, the ACE2 vs Silibinin complex (**Fig 3A**) has five H-bonds with Gln102,108,398, ARG514, and SER511 residues and also has π -alkyl, Alkyl and other hydrophobic interactions with residue of the ACE2 receptor protein. The Spike vs Beta amyrin complex (**Fig 3B**) has one H-bonds with ALA82 residue. In the case of the MAPK8 vs Taraxerol complex (**Fig 3C**), taraxerol showed no conventional hydrogen bonding but formed significant hydrophobic interaction with THR65, LYS68, ARG69, ARG72, LEU172, ALA173, ALA176, THR178, SER179, GLU177, ARG192, VAL196, and GLY199 residues respectively (**Table 2**). However, other two complexes of RdRp and Mpro shows a good interaction as well, but the interaction of the complexes of ACE2, Spike, and MAPK8 were better than that two of Mpro and RdRp (S1 Fig).

**Table 2:** The key molecular interactions of the selected protein-ligand complexes and their interaction types.

Receptors (Proposed)	Drug molecules (Proposed)	Interacting Amino acid
ACE2	Silibinin	Hydrophilic: Gln102,108,398, ARG514, and SER511 Hydrophobic: LYS187,562, TYR199,510,196,202, ASP509, 206, TRP566,203, GLY205, LEU95, ALA396, GLN98
Spike (S)	Beta Amyrin	Hydrophilic: ALA82 Hydrophobic: GLN85, LEU56,83,374, TRP52, PHE23,373, ARG376, ASP333, TYR368, ASN377
MAPK8	Taraxerol	Hydrophilic: None Hydrophobic: THR65, LYS68, ARG69, ARG72, LEU172, ALA173, ALA176, THR178, SER179, GLU177, ARG192, VAL196, GLY199,

To validate the molecular interaction of the proposed drugs with our top-ranked receptors (ACE2, S, and MAPK8), we performed site specific docking analysis of the target protein with both reference drug (FDA approved) and proposed drug (S5 Table). The molecular interaction between proposed drugs and reference drugs displayed in S2 Fig, it observed that the interaction types with interacting residues between proposed and reference drugs are almost similar and at least two amino acid residues are common in both proposed and reference (receptor-ligand) complexes. In ACE2 receptor protein both silibinin and ritonavir interacting with three common residues (ALA348, GLU375, and HIS378), in case of MAPK8 both taraxerol and nirmatrelvir the interacting common residues are LEU172 and ALA173, whereas in case of spike protein the common residues interacting with beta-amayrin (**Fig 3C**) and remdesivir are (ASP365 and HIS384). Therefore, our proposed drugs have a good interacting properties and binding affinity score as compared with reference drugs.

#### 3.2.2. Re-docking with co-crystalized ligand of the top-ranked receptors.

**Fig 4:**
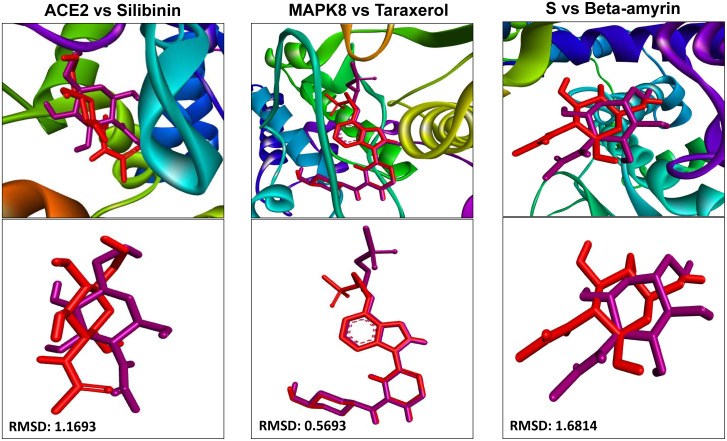
The re-docking of the co-crystal ligand originally in PDB file and superimposition of docked ligand (red color) on the co-crystalline ligand (magenta color) for target protein.

The validation of the docking study is generally performed by re-docking the co-crystal ligand presents at the binding site. Calculating the RMSD of the docking poses in comparison to the co-crystal pose of the ligand to compare between the position of experimental ligand and the docked poses. In this study the RMSD of the co-crystal ligand NAG (2-acetamido-2-deoxy-beta-D-glucopyranose) and 1BK (trans-4-[(4-{4-[3-(methylsulfonyl)propoxy]-1H-indazol-1-yl}pyrimidin-2-yl)amino]cyclohexanol) was calculated (**Fig 4**) for the best binding poses with their corresponding protein ACE2 (2AJF), S (7T9K), and MAPK8 (4HYU) [98–100]. In structure-based drug design and docking validation, a re-docking RMSD <2.0 Å between the re-docked ligand and its crystallographic (co-crystal) pose is generally considered as standard value, as it indicates that the docking protocol can reliably reproduce experimentally observed binding modes [101]. The RMSD of the co-crystal ligand by re-docking was 0.5693, 1.1693, and 1.6814 Å for taraxerol, silibinin and beta-amyrin respectively. Our study, the majority of re-docked co-crystal ligands RMSD values are within the range with <2.0 Å, validating the accuracy of our docking parameters and scoring function. Here the beta-amyrin shows close to the standard values with 1.6814 Å, which suggest a slight conformational differences, possibly due to ligand flexibility, shallow binding pockets, or protein dynamics not fully captured by rigid docking methods. These findings confirm the validity of our docking setup and justify the subsequent binding energy and interaction analyses.

### 3.3. MD Simulation of the top-ranked phytocompounds

**Fig 5:**
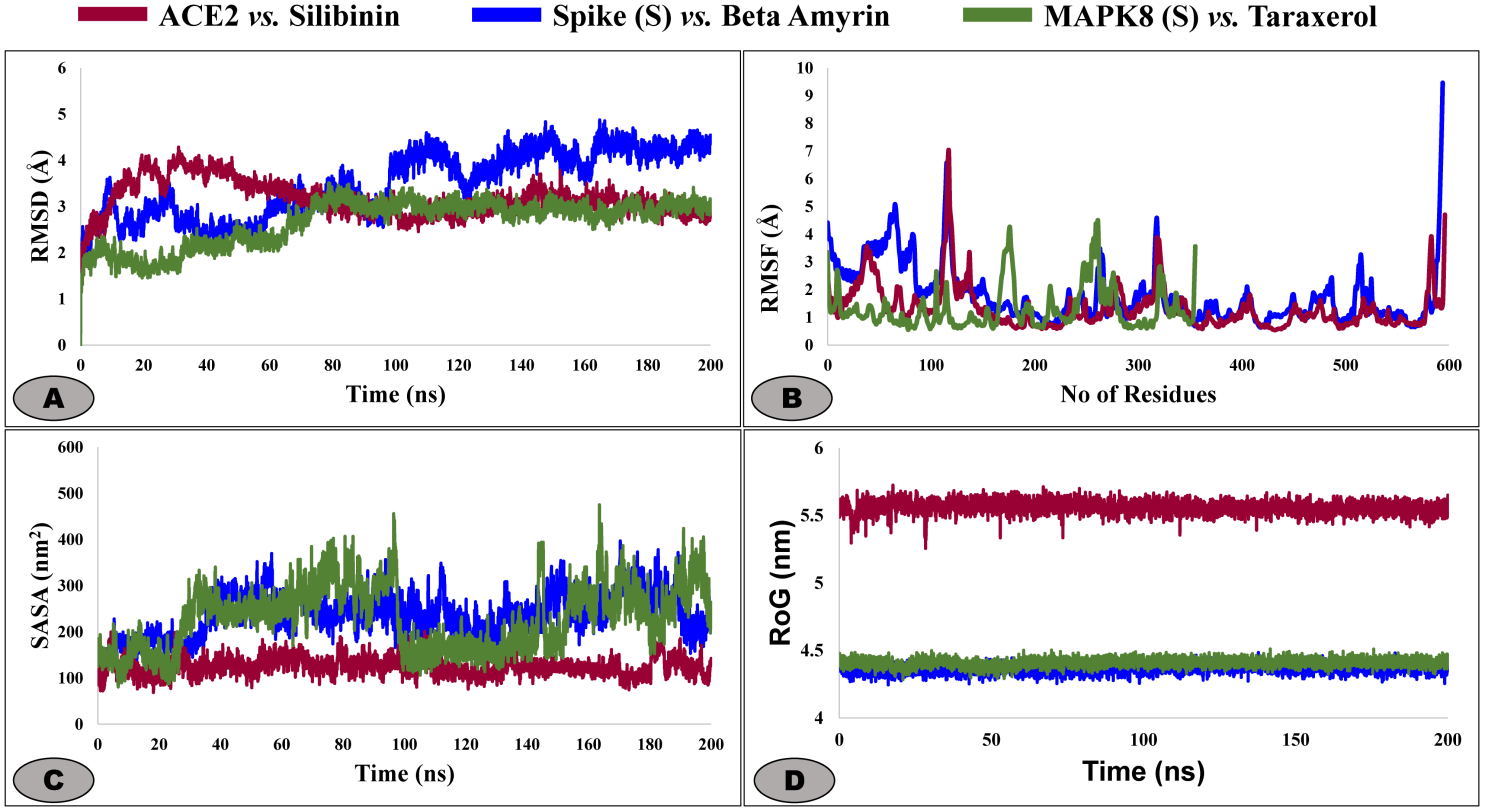
(A) The representation of the Root Mean Square Deviations (RMSD), (B) Root mean square fluctuations (RMSF), (C) Solvent accessible surface area (SASA), and (D) the Radius of gyration (Rg) plot in (nm) of the selected complexes.

From the BAS and P-L interaction analysis, we observed that ACE2 vs Silibinin, MAPK8 vs Taraxerol, and S vs Beta amyrin are the top-ranked complexes based on their higher binding affinities (**Fig 5**). Therefore, we selected these three complexes for their stability analysis by 100 ns MD simulation. The RMSF, RMSD, SASA, Rg, H Bonds, and MM-GBSA bonding energy were calculated using trajectories to concisely evaluate the results of MD simulation.

#### 3.3.1. Root mean square deviation (RMSD).

The amount of conformational change experienced by a molecular system relative to the original conformation at t = 0 can be calculated using the simulated time curve of RMSD. **Table 3** showed that the complex ACE2_Silibinin and MAPK8_Taraxerol have the average RMSD value of 3.16 and 2.66 Å respectively. During the whole simulation time ACE2 and MAPK8 complexes shows deviation from its mean position up to 80 ns (**Fig 5A**). These two complexes seems to be stable after 80 ns and their deviation range was within the standard range 2.5–3.0 Å [102,103] up to 200 ns. Whereas, the S_beta amyrin complex shows the higher fluctuation within 2.0–3.0 Å up to ~100 ns, with an average value of RMSD 3.48 Å and after 100 ns the RMSD plots shows the increased deviation up to 4.0 Å. It indicates that S_Beta amyrin complex is comparatively less stable than the ACE2_Silibinin and MAPK8_Taraxerol complexes. Thus, it could conclude that, the ligands have decent binding interaction with the target protein during the simulation period.

**Table 3:** The calculated average values of RMSD, RMSF, Rg, SASA, and MM-GBSA based binding free energy (∆G) for all protein-ligand complexes.

Name of ligand-protein complex	MD simulation Study				
	RMSD (Å)	RMSF(Å)	RoG (nm)	SASA (nm^2^)	Binding Energy (kcal/mol)
ACE2 vs Silibinin	3.16	1.33	5.56	124.72	−38.27
S vs Beta-amyrin	3.48	1.79	4.36	236.27	−24.67
MAPK8 vs Taraxerol	2.66	1.31	4.41	229.87	−22.84

#### 3.3.2. Root mean square fluctuations (RMSF).

To quantify functionality, flexibility and mobility of the ligand-protein complex can be explained by using the RMSF graph. The RMSF graph show the fluctuation and dynamic behavior of a specific atoms or, residues of protein structure from their mean position during the simulation time. The regions with higher RMSF values are more flexible, whereas regions with lower RMSF values are more rigid [104]. **Fig 5B** represent the individual residual RMSF diagram of each molecular system and the average value of each complex are given in **Table 3**. The estimated average RMSF value for ACE2_Silibinin, S_Beta-amyrin, and MAPK8_Taraxerol are 1.33, 1.79, and 1.31 Å respectively (**Table 3**). The plots represent three different proteins backbone residual plots so the number of amino acid residues are different for each complex. The Fig 5B, shows that MAPK8 system have the lowest RMSF than the ACE2 and Spike, which indicate that the complex mostly rigid and remain stable throughout the simulation period. This stability can be due to being part of well-structured regions like α-helices, β-sheets, or other secondary structural elements.

#### 3.3.3. Solvent accessible surface area (SASA).

SASA is a crucial measurement to calculate the surface area of a biomolecule (such as a protein, nucleic acid, or complex) that is accessible to a solvent. SASA is an important concept for understanding the interactions of biomolecules with their environment, including solvent molecules and other biomolecules. The average value of SASA for each complex (ACE2_Silibinin, S_Beta-amyrin, and MAPK8_Taraxerol) are 124.72, 236.27, and 229.87 nm^2^ respectively (**Table 3**). The higher SASA values for S_Beta-amyrin, and MAPK8_Taraxerol suggest that these complexes have larger or more exposed surfaces compared to ACE2_Silibinin (**Fig 5C**). The significantly lower SASA for ACE2_Silibinin likely indicates a smaller or more compact complex with less solvent-exposed surface area. Thus, the ACE2_Silibinin complex is expected to be more stable than the S_Beta-amyrin, and MAPK8_Taraxerol complexes due to its lower SASA.

#### 3.3.4. Radius of gyration (Rg).

The radius of gyration (Rg) is an important metric in MD simulations for understanding the compactness and stability of P-L complexes. By analyzing Rg along with other measures like RMSD and SASA, researchers can gain comprehensive insights into the structural dynamics, stability, and conformational changes of these complexes, ultimately aiding in the interpretation of simulation results and the design of more stable and effective drugs. The average value of Rg for each complex (ACE2_Silibinin, S_Beta-amyrin, and MAPK8_Taraxerol) are 5.56, 4.36, and 4.41 nm respectively (**Table 3**). The plots suggest that, the S_Beta-amyrin, and MAPK8_Taraxerol system shows the similar trends for RoG, indicating comparable sizes and degrees of compactness, the highest Rg value of ACE2_Silibinin indicating a comparatively less compact structure (**Fig 5D**). Therefore, the findings of this study suggest that, S_Beta-amyrin and MAPK8_Taraxerol complex is more stable compared with ACE2_Silibinin system.

#### 3.3.5. Total hydrogen bonds calculations.

**Fig 6:**
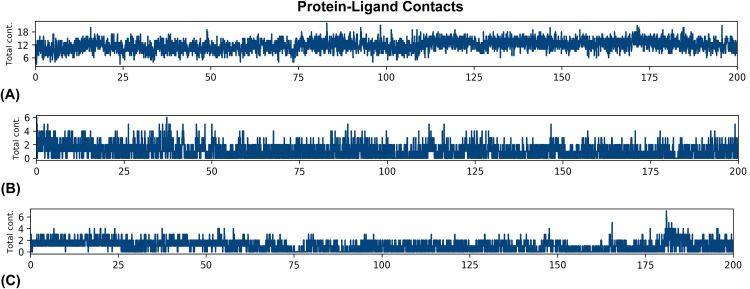
Hydrogen bond profiles of the top-ranked protein-ligand complex: (i) ACE2_Silibinin (Red) (ii) S_Beta-amyrin (Black) (iii) MAPK8-Taraxerol (Green).

High binding affinity is primarily accompanied by H-bonding, which is an important indicator of strong P-L interaction [105,106]. The amount of hydrogen bonds in a P-L interaction usually increases the inhibitor’s activity against the target protein. The given Figure shows the amount of H bonds formed between P-L complexes that were calculated using the MD trajectories. **Fig 6A** showed larger number of P-L contacts, indicating stable binding than others. Whereas, the Beta-amyrin and Taraxerol bound to S and MAPK8 protein complex shows similar trends for forming P-L contact or, H bonds (**Fig 6B and 6C**). The maximum bond formation between these two P-L systems is six, whereas silibinin formed maximum 20 bonds with its corresponding protein structure.

#### 3.3.6. MM-GBSA binding free energy.

**Fig 7:**
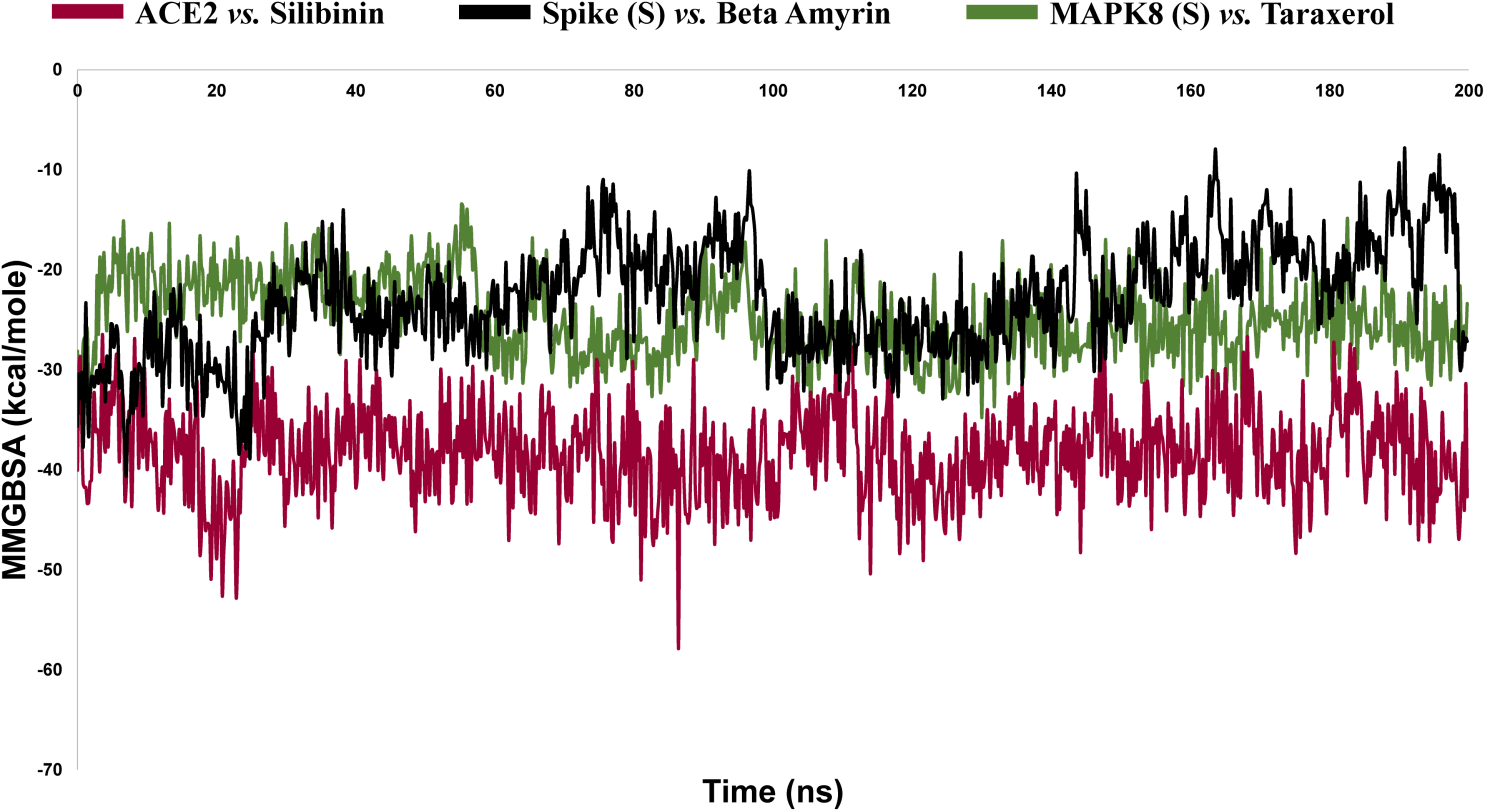
The graphical representation of the MM-GBSA based binding free energy (BFE) using the trajectories from 200 ns MD simulation.

To estimate the binding free energy (ΔG_bind_) or BFE of ligand-protein Molecular Mechanics Generalized Born Surface Area (MM-GBSA) is a widely accepted computational techniques, that calculate the ΔG_bind form the MD trajectories. The MM-GBSA based BFE of the docked P-L complexes were calculated to validate the binding affinity of the selected phytocompounds to their corresponding receptor protein. In our study, we calculated the MM-GBSA to estimate the BFE for 1001 frames with interval of 5 steps out of 5000 frames, and the outcomes were illustrated in **Fig 7**. Generally, the negative BFE indicate the binding of the ligand to the target protein is thermodynamically favorable and spontaneous [107,108]. According to this **Fig 7**, S_Beta-amyrin and MAPK8_Taraxerol showed almost the similar trend of BFE with average BFE value of −24.67, and −22.84 kcal/mol respectively (**Table 3**) which indicate that, both complexes have almost same energy value. The average BFE of ACE2_Silibinin is −38.27 kcal/mol which is negatively higher than others, indicating the better binding compared to MAPK8 and S systems with their corresponding ligands.

#### 3.3.7. Principal component analysis.

**Fig 8:**
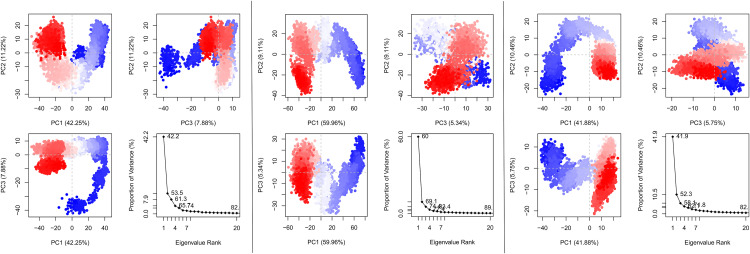
Principal Component Analysis (PCA) plots showing the dynamic behavior of (A) ACE2 *vs.* Silibinin, (B) Spike *vs.* Beta Amyrin, and (C) MAPK8 *vs.* Taraxerol. Where, the fluctuation pattern across structures clarified by color mapping, the red dots representing the least dynamic fluctuations (stable region), the white dots suggesting moderate movements, and the blue dots indicating the highest conformational change (dynamic region).

Principal component analysis (PCA) was used to compare the conformational change and flexibility of the three different P-L complexes of silibinin, beta-amyrin, and taraxerol. PCA analyzed the backbone atoms of the complex system by evaluating three principal conformations (PC1, PC2, and PC3) using standard MD simulation. The variance captured by PC1 ranged from 41.88% to 59.96% across the different systems, indicating varying levels of dominant structural rearrangements. The P-L complex of S_beta amyrin and ACE2_silibinin showed high PC1 variances of 59.96% and 42.25%, respectively (**Fig 8A & 8B**), indicating dominant conformational changes. Conversely, MAPK8_taraxerol induced the strongest conformational constraint, as reflected by its lower PC1 (41.88%), along with balanced variances in PC2 (10.46%) and PC3 (5.75%), forming a tightly clustered and stable trajectory (**Fig 8C**). These findings suggest that taraxerol confers the highest degree of structural stabilization to MAPK8, highlighting its potential as a promising therapeutic candidate.

### 3.4. Pharmacokinetics (ADME/T) analysis

#### 3.4.1. ADME analysis.

**Fig 9:**
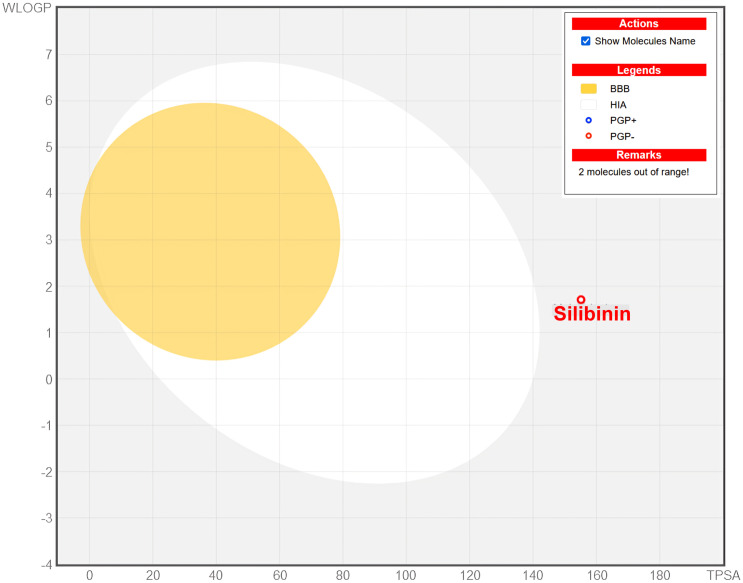
The graphical illustration of BIOLED-Egg model of the selected candidate drug molecules.

To assess the druggability and pharmacokinetics (PK) analysis for the newly suggested three drug molecules (silibinin, taraxerol, and beta-amyrin), we computed the ADME properties by using the pkCSM and SWISSADME web-tools, respectively (**Table 4**). According to the BOILED-Egg model (**Fig 9**), the selected compounds are not able to cross the blood brain barrier (BBB) and has no side effects on central nervous system (CNS). Thus, these drugs are safe and has reduced side effect on human body than other established drugs. Therefore, none of the selected compounds can cross the BBB, and they have low to moderate volumes of distribution, indicating limited tissue penetration and interaction to the CNS.

**Table 4:** Calculation of pharmacokinetics (ADME) characteristics of the selected top-ranked phytocompounds.

Compounds	Absorption		Desorption		Metabolism (inhibition)				Excretion	
	Caco2	HIA	BBB	VDs	CYP1A2	CYP2C19	CYP3A4	CYP2D6	TC	T_1/2_
Silibinin	0.5808	0.97	No	0.64	No	No	No	No	3.54	0.336
Taraxerol	0.8310	1.0	No	1.33	No	No	No	No	5.58	0.129
Beta Amyrin	0.8310	1.0	No	1.26	No	No	No	No	4.89	0.057

*Note: No = this property does not exist. Yes = this property does exist;*

According to ADME properties, the predicted Caco-2 permeability of the selected compounds is given as the log cm/s. A compound is considered to have a proper Cao-2 permeability if it has predicted value Papp<8 × 10 − 6 cm/s or if it has a value of Papp≥8 × 10 − 6 cm/s then it is considered as high Caco-2 permeable compounds [109]. Silibinin has proper Caco-2 and high HIA absorption property (>80%) whereas both of taraxerol and beta-amyrin has high Caco-2 and HIA absorption properties with a value of 0.831 and 1.0 or 100% respectively. None of the proposed compounds are able to enter the BBB which indicate that this could be help to reduce the CNS related side effect than the synthetic drugs others. The volume of distribution for both of Taraxerol and Beta Amyrin are moderately distributed which is generally preferable for SARS-CoV-2 treatment, as it allows the drug to reach and act in various tissues where the virus might reside and also the metabolic properties are characterized by the absence of CYP1A2, CYP2C19, CYP3A4, and CYP2D6 inhibition, indicate that these compounds are less likely to cause drug-drug interactions, which is an important benefit in treating SARS-CoV-2, as patients often require multiple drugs to manage symptoms and complications. The total clearance of the metabolic drug from the human body is high for taraxerol and beta amyrin as 5.58 and 4.89 mL/min/kg respectively whereas the clearance for silibinin is about 3.54 mL/min/kg indicates moderate clearance.

#### 3.4.2. Aquatic and non-aquatic toxicity analysis.

The toxicity of the proposed drug molecules was investigated including AMES toxicity, carcinogenicity, predicted toxicity class, cytotoxicity, hepatoxicity, and skin sensitization through the pkCSM and ProTox web-tool which indicated that the selected molecules are non-toxic. The AMES were found in optimal range of 0–0.3 [84,110], which indicates that all three suggested drug molecules are non-toxic. The scale of predicted toxicity class is ranging from 1 (toxic) to 6 (non-toxic) and the data shows that the proposed compounds are non-toxic in nature. All the suggested drug molecules were inactive for carcinogenicity, cytotoxicity, and hepatotoxicity (**Table 5**). The skin sensitization values shows that none of our proposed drug molecules exceeded the optimal score and they are safe.

**Table 5:** Toxicity analysis of the selected top-ranked phytocompounds.

Compounds	AMES Toxicity	Carcinogenicity	Predicted Toxicity Class	Skin sensitization	Cytotoxicity	Hepatotoxicity
Silibinin	0.311	Inactive	4	0.141	Inactive	Inactive
Taraxerol	0.001	Inactive	6	0.896	Inactive	Inactive
Beta Amyrin	0.001	Inactive	6	0.671	Inactive	Inactive

#### 3.4.3. Medicinal chemistry.

**Fig 10:**
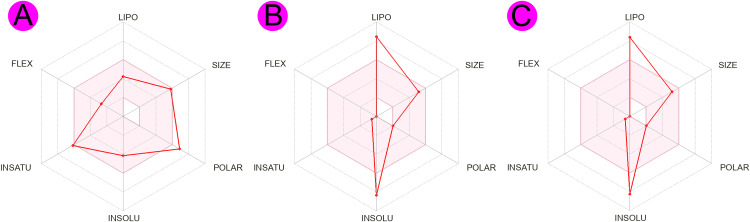
The bioavailability radar image of the selected top-ranked phytocompounds (A) silibinin, (B) taraxerol, and (C) beta-amyrin. Where the colored (pink) region represents the optimal range for each property of the candidate drug molecules.

The estimated properties for oral bioavailability, structural assays for medicinal chemistry parameters, and lead likeness properties of the top-ranked suggested candidate drug molecules are given in the **Table 6**. The oral bioavailability of a drug molecule is the percentage of the dosage that ultimately reaches the therapeutic site; it is represented quantitatively as %F [111]. The bioavailability score of all selected candidate drug molecules (silibinin, taraxerol, and beta-amyrin) is about (0.55 or 55%) in scale of 1.0 or 100% [112]. In the **Fig 10** the colored region represents the optimal area for the oral bioavailability radar. In medicinal chemistry, there is no structural alerts for the selected compounds according to PAINS method and in case Brenk there is an alert for (isolated_alkene group) present in taraxerol and beta-amyrin [113]. The silibinin has 1 violation in lead-likeness for its MW, whereas the taraxerol and beta-amyrin has 2 violations due to their MW and LogP value. The synthetic accessibility score measure in scale 1(easy) to 10 (very difficult), the estimated synthetic accessibility score for our selected compounds silibinin, beta-amyrin, and taraxerol 4.92, 6.04, and 6.04 respectively [89].

**Table 6:** Predicted medicinal chemistry, oral bioavailability, and lead-likeness of the selected top-ranked phytocompounds.

Compounds	Bioavailability Score	PAINS (alerts)	Brenk (alerts)	Leadlikeness (violation)	Synthetic accessibility
Silibinin	0.55	0	0	1	4.92
Taraxerol	0.55	0	1	2	6.04
Beta-amyrin	0.55	0	1	2	6.04

### 3.5. Density Functional Theory (DFT) analysis

#### 3.5.1. Optimized energy and dipole moment analysis.

The interactions of drug molecules with the receptor proteins and their biological functions are significantly influenced by their molecular structure, conformation, and geometry [44]. In **Table 7**, we observed that optimization energy values for silibinin is about −1717.004 Ha, whereas the optimized energy for taraxerol and beta-amyrin is almost similar. Therefore, silibinin has the least value of total energy than that of taraxerol and beta-amyrin, hence silibinin has the more stable structure compared to the others. The dipole moment of silibinin 7.28 D higher than the value of taraxerol (1.84 D) and beta-amyrin (1.78 D). This indicates that a larger dipole moment in the case of drug molecules suggests that the molecule has a stronger polarity, which means it has a better ability to interact with other polar molecules or binding sites/pockets.

**Table 7:** Data table for calculated optimization energies and other quantum chemical descriptors from density functional theory (DFT) calculation.

Parameter	Silibinin	Beta-amyrin	Taraxerol
Optimized energy (Ha)	−1717.004	−1248.472	−1248.457
Dipole moment, D	7.28	1.84	1.78
HOMO energy (E_HOMO_)	−0.21907	−0.22918	−0.23043
LUMO energy (E_LUMO_)	−0.07091	0.01961	0.01609
Energy Gap (∆E)	0.1382	0.2488	0.2465
Ionization potential [I = −E_HOMO_]	0.21907	0.22918	0.23043
Electron affinity [A = −E_LUMO_]	0.07091	−0.01961	−0.01609
Chemical hardness [ɳ = (I − A)/2]	0.07408	0.124395	0.12326
Softness (σ = 1/ ɳ)	13.4989	8.03891	8.11293
Electro-negativity [χ = (I + A)/2]	0.14499	0.104785	0.10717
Chemical potential (μ = − χ)	−0.14499	−0.104785	−0.10717
Electrophilicity (ω = χ^2^/2ɳ)	0.14189	0.04659	0.04413

#### 3.5.2. Frontier molecular orbital (FMO) analysis.

**Fig 11:**
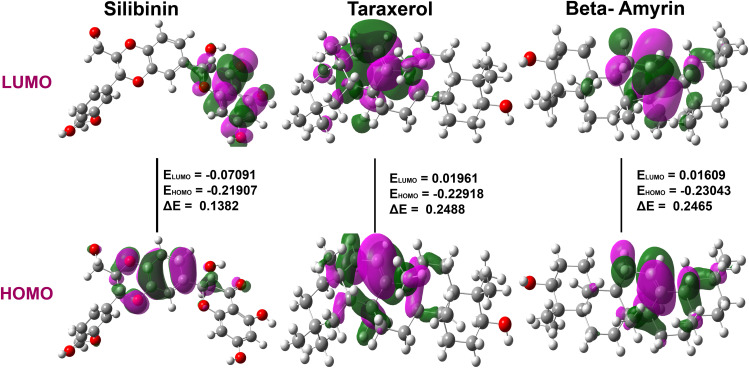
Frontier molecular orbitals diagram for HOMO and LUMO of top-ranked three phytocompounds (Silibinin, Taraxerol, and Beta-amyrin), regions where the wavefunction is positive (Purple) tend to contribute to bonding interactions, while negative regions (Green) might contribute antibonding interactions.

The FMO analysis was performed to investigate the reactivity and the reactive region in a compounds molecular system. The **Fig 11** represents the HOMO and LUMO molecular orbitals in details with their energy gap (∆E) value. In this figure the orbitals with red color indicate the positive field and the orbitals with green are negative field. The HOMO and LUMO energy gap serve as an indicator for the kinetic stability of the molecule. Generally, a smaller HOMO**–**LUMO energy gap denotes higher chemical reactivity, which can facilitate the better binding of the molecule within the key residues [114]. Whereas, the higher HOMO**–**LUMO energy gap indicates the lower reactivity and stability to being an inert molecule [85,92]. From the data **Table 7** it can be seen that, the HOMO energy levels are −0.21907, −0.22918, and −0.23043, while the LUMO energy levels are −0.07091, 0.01961, and 0.01609 Hartree (Ha) respectively. Thus, the compounds with less energy gap are silibinin (i.e., 0.1382) this indicate that these compounds are relatively more reactive comparing with taraxerol and beta-amyrin.

#### 3..5.3. Quantum chemical descriptors analysis.

A theoretical strategy for linking the chemical bioactivity of molecular structures to their electronic properties is represented by Koopmans’ theorem [115], this theorem is important to calculate the quantum chemical descriptors that helps to identify a molecule’s reactivity. The DFT method was used to calculate the descriptors values. These values were calculated using the mathematical equation as previously reported in the method section [116]. **Table 7** shows the all-parameter related to chemical reactivity, where the electronic affinity (A) is a measure of the tendency of an atom or molecule to attract an additional electron. While a molecule with a high electronic affinity value is more likely to accept electrons. Comparing the ionization potential and electron affinity values of the three suggested drug molecules, it can be observed that, molecule silibinin has the highest electron affinity, A (0.0709 Ha) and lowest ionization potential, I (0.21907 Ha), while molecule taraxerol and beta-amyrin has the lowest A value −0.01609 and −0.01961 Ha respectively. This suggests that molecule silibinin has the strongest tendency to attract additional electrons compared to taraxerol and beta-amyrin, whereas a higher ionization potential value suggests that the molecule need more energy to remove an electron, which may affect its reactivity or stability. Among the hit compounds, the values of hardness (ɳ) and softness (σ) between the three molecules, we can see that molecules taraxerol and beta-amyrin have approximately similar values for both parameters, while silibinin have much lower values of hardness (0.07408 Ha) and higher values of softness (13.4989 Ha). This indicates that the silibinin is more reactive and disposed to changes in their electronic structure than taraxerol and beta-amyrin. All of the compounds’ chemical potentials (μ) show negative values, indicating good stability and creating a stable complex with the receptor.

#### 3.5.4. Molecular electrostatic potential (MEP) analysis.

**Fig 12:**
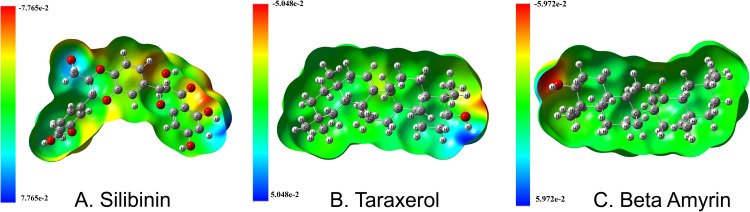
Charge distribution MAP of Molecular Electrostatic Potential (MEP) for the selected phytocompounds (Silibinin, Taraxerol, and Beta amyrin), where the region of positive potential represented by deep blue, green color show neutral potential zones and the negative one is represented by deep yellow.

Molecular Electrostatic Potential (MEP) calculation for the selected phytocompounds silibinin, beta-amayrin, and teraxerol are illustrated in the **Fig 12**. This map is used to identify the electrophilic and nucleophilic reactive sites of a molecule based on the electrostatic potential distribution [117,118]. Furthermore, the MEP surface offers valuable insights into potential intramolecular and intermolecular hydrogen bonding by emphasizing likely regions for hydrogen bond donors and acceptors. It can be clearly seen that; the positive potential areas are on H atoms and the negative areas are spread out over the electronegative atoms (Oxygen and Sulfur). Thus, the positive electrostatic potential and the negative electronegative potential are more promising for electrophilic and nucleophilic interaction [119]. The color code for compounds Silibinin, Taraxerol, and Beta amyrin ranges from −7.765e^-2^ to +7.765e^-2^, −5.048e^-2^ to +5.048e^-2^, and −5.972e^-2^ to +5.972e^-2^, respectively, where the color Red and blue in the map structures indicate more electron-rich and electron-poor region, respectively.

## 4. Discussion

To explore potential phytocompounds of BC as the drug molecules against SARS-CoV-2 infections integrating bioinformatics approaches, at first, we systematically reviewed 100 articles that suggested SARS-CoV-2 infection causing KGs. We found around 158 KGs associated with the infections and finally select the top-ranked 11 drug target proteins/receptors based on their highest frequency as a drug target. In our study we choose black cumin (BC) as a source of naturally occurring phytocompounds to explore the candidate drug molecules. Generally, BC contain two main types of groups (i) volatile/essential oil (e.g., thymoquinone and dithymoquinone), and non-volatile lipids (e.g., linoleic and oleic acids), along with notable broad chemical groups including, triterpenoids/sterols (e.g., beta-amyrin, taraxerol, 24-methylene-cycloartenol and stigmasterol), saponins (e.g., alpha-hederin), alkaloids (e.g., nigellidine), vitamin (e.g., folic acid), and some other minor groups [120–123]. A total of 300 phytocompounds derived from BC seed and other part of the plant were retrieved from an Indian database called IMPPAT [77] as SMILE file format and then we collect the 3D SDF file from PubChem database [124]. After removing the duplication, we found 241 unique BC phytocompounds.

The drug likeness properties of 241 compounds were performed to evaluate their physicochemical properties applying LRO5 and VR. In conventional, orally active drugs should not cross more than two Lipinski rules, otherwise, its bioavailability is compromised [81]. According to these results there are some exceptions such as, lipophilicity index (LogP), this high lipophilicity constitutes a violation of LRO5. High lipophilicity may sometimes lead to low aqueous solubility, reduced absorption, altered membrane permeability, increased risk of non-specific binding, and significant effects on metabolic stability or pathways in drug development sector. Though, the LRO5 cannot describe a compound’s whole physicochemical nature, the factors that determine lipophilicity have a significant impact on the behavior of organic compounds such as pharmaceuticals or drug targets [125,126]. Considering this, many medicines violate one or two of Lipinski’s criteria while remaining effective. Several therapeutic drugs, have higher MW than the standard value (for example, Erythromycin MW:733, Cyclosporine with LogP 7.5; MW:1202, Vancomycin MW: 1449, and Insulin has H-bond Acceptor 83, Donor atom 73; LogP −13.1; MW:5700 and etc.) [127–129], yet they are still used as medications. However, Lipinski’s principles include exceptions, and certain molecules that contravene them may nonetheless be orally accessible and effective medications [130,131]. Finally, a total of 206 filtered phytocompounds were passed through the rule of Lipinski and has only one (LogP) violation in drug likeness properties (see S5 Table).

In order to identify potential therapeutic, we performed molecular docking analysis of the reduced 206 BC phytochemical against the proposed top-ranked 11 receptor proteins/protease. From docking results, we selected top-ranked five phytocompounds Silibinin, Taraxerol, Beta-amyrin, Folic acid and 24-methylenelophenol as the candidate drugs based on their highest average BAS with −9.60, −9.10, −8.90, −8.60, and −7.50 kcal/mol, respectively. It should be mentioned here that the BAS of the proposed phytocompounds much larger than the BAS −7.18, −6.80, −5.81, −5.55, −5.52, −5.50, and −5.35 of the previously suggested major compounds nigellidine, dithymoquinone, nigellamine, carvacrol, thymol, thymohydroquinone, and thymoquinone, respectively [47,53–55,58,62]. Therefore, the proposed phytocompounds might be more effective compare to the previously recommended major phytocompounds.

For further *in-silico* validation of the proposed phytocompound, the protein-ligand (P-L) interaction analysis were performed. From their ligand’s interaction to the corresponding target protein, we finally select top three phytocompounds based on their BAS and interactions with the amino acid residues of the target protein. The highest BAS for these three compounds (Silibinin, Taraxerol and Beta amyrin) with the receptor proteins ACE2, MAPK8, and Spike (S) proteins were −9.60, −9.10, and −8.90 kcal/mol, respectively (Fig 2A). High binding affinity is primarily accompanied by H-bonding, which is an important indicator of strong P-L interaction [132,133]. The amount of hydrogen bonds in a P-L interaction usually increases the inhibitor’s activity against the target protein. As shown in Fig 3, the ACE2_Silibinin complex (Fig 3A) has 5 H-bonds with Gln102,108,398, ARG514, and SER511 respectively. The S_Beta amyrin complex has Only one H-bonds with ALA82 (Fig 3C_Left), but in LigPlot view it shows two H-bonds with the corresponding residues ALA82 and GLN85(Fig 3C_Middle). In the contrast, MAPK8_Taraxerol complex shows no significant hydrogen bonds (Fig 3B). All the hydrophilic and hydrophobic interactions are shown in Table 2 for top three P-L complex. Though, the complex of RdRp_Folic acid shows one conventional H-bonds and one unfavorable week interaction, whereas the Plpro_24-Methylenelophenol has no significant conventional hydrogen bonding in their interaction but has hydrophobic interaction with the key residue of the target protein (S1 Fig). Additionally, validate the proposed top-ranked phytocompounds (Silibinin, Taraxerol, and Beta amyrin) as drug molecules we performed further docking analysis with six FDA approved drugs and observed significant binding affinities with the proposed 11 receptor protein (Fig 2B). Where, the FDA suggested RDs ritonavir shows the highest binding score (−9.20 kcal/mol) against ACE2 whereas our suggested compound silibinin shows highest affinity against ACE2 with −9.60 kcal/mol. Furthermore, the top-ranked three phytocompounds (Silibinin, Taraxerol, and Beta amyrin) was considered for further investigation based on their molecular interaction with the amino acid residue of the target protein for MD simulation studies.

In drug design procedure, re-docking is a method which helps to validate the docking protocols. We calculate the RMSD of co-crystallized ligand of the top-ranked target protein to validate the docking protocols, where it can be observed that none of the ligands RMSD crosses the standard range (2.0 Å), which indicates that the docking protocol can reliably reproduce experimentally observed binding modes [134,135]. However, when the RMSD values exceed this reference value, several implications need to be considered, such as limitations in the scoring function, inadequate sampling, or low resolution of the target protein structure [134]. Consistently high RMSDs should prompt a review of the docking setup to ensure its reliability for predicting novel ligand binding. Therefore, finding of this study suggest that these top-ranked three (Silibinin, Taraxerol, and Beta amyrin) might have significant therapeutic effect for SARS CoV-2 infections.

Therefore, we considered these top-ranked three P-L complex for MD simulation, which is a powerful method for studying the dynamic behavior of biomolecular systems, to anticipate drug-target interactions [136,137]. To concise the findings of MD simulation, many key parameters including (RMSD, RMSF, SASA, Rg, H-Bonds & MM-GBSA BE) that provide insight into the mobility and dynamic behavior of the molecular system. The RMSD value of the P-L complexes helps to understand the structural stability and illustrates, how a ligand fit to the proteins active site [22]. The **Fig 5A** shows the RMSD profiles of the selected complex of ACE2_Silibinin, S_beta amyrin, MAPK8_Taraerol, this graph indicates that the system reached equilibrium after 40**–**45 ns and maintained a consistent deviation over the simulation time. Where, the ACE2_silibinin complex shows the average RMSD score about 3.16 Å and it fluctuate after its equilibration processes (2.25 to 3.25 Å), which showed comparatively low and stable performance of the system, suggesting high structural integrity and minimal confirmational movements. Whereas, the Spike complex fluctuated up to 100 ns between 2.5 to 3.0 Å, after 100 ns it deviated to ~4.30 Å and remain comparatively stable than the first 100 ns with higher flexibility [102,103]. The fluctuation could be caused by the absence of the ligand in the receptor’s binding site, causing the protein to explore greater conformational space [104,138]. The lowest RMSD was observed for the system of MAPK8_Taraxerol with ~2.5 Å RMSD values, indicating the strong binding stability with lowest deviation from its initial points. RMSF provides insights into the flexibility and dynamic behavior of different regions of the protein residues, particularly the active sites of the target protein. Regions with higher RMSF values are more flexible, whereas regions with lower RMSF values are more rigid and stable [137,139]. In our study, the maximum number of residues exhibited fluctuations of up to 2.0 Å, with higher peaks observed in specific regions and at terminal residues. These regions may not directly participate in the ligand binding, but contribute to the confirmational movements of the ligands and allosteric regulation. The S_Beta-amyrin and MAPK8_Taraxerol had the highest average RMSF values, 1.79 and 1.31 Å, respectively. The RMSF plot depicts that all the residues of the complexes were in the lower range of 1.0–4.0 Å, which indicates that the lower RMSF of these complexes is mostly rigid and remains stable throughout the simulation period [139]. It’s interesting to note that RMSF peaks matched up with flexible loop regions that could affect the movements of binding pockets. For example, In the ACE2–Silibinin complex, the moderate flexibility (~2.5–3.5 Å) seen around residues 30–60 probably corresponds to areas around the receptor-binding domain, which are known to be significant for ligand accommodation and induced fit. For the Spike–Beta Amyrin complex, the flexibility near residue ~110 could be linked to the receptor-binding motif, which is necessary for interacting with host receptors. This means that Beta Amyrin may stabilise or change this interface. In MAPK8–Taraxerol, flexible residues around ~215–250 near the activation loop of MAPK8 may help control the kinase. Overall, the RMSF plots suggest that these flexible loops, while not central to direct ligand binding but helps to allosteric regulation or confirmational adaptability, which are important for the biological function of the proteins. The SASA plots suggest that ACE2 is more stable than the Spike and MAPK8 system. The SASA plot shows that Spike and MAPK8 has the similar trend for their system compactness (**Fig 5C**) due to the dynamic loop movements. In contrast, the ACE2 complex has the consistently lower SASA (~150–200 nm^2^) value than others, indicating a compact and less solvent-exposed structure. This lower value suggests that, the residues are tightly packed and more stable. The RoG plots support the above observation, ACE2_silibinin complex maintain a high RoG value (~5.6 nm), suggesting the distribution uniformity and structures compactness during the simulation time. On the other hand, the S-Beta amyrin and MAPK8-Taraxerol follow the similar trends within (~4.4–4.5 nm) and remain stable within this range. This confirms that no significant structural expansion occurred during the whole simulation time frame. Therefore, the consistent SASA and RoG results support the hypothesis that all complexes stayed structurally compact and stable in physiological condition that we set during the MD simulation. The ACE2_Silibinin complex have the larger number of H bonds indicating stable binding than S_Beta-amyrin and MAPK8_Taraxerol which showed the almost similar trend for H bond formation. The number of hydrogen bond formation may vary due to the interaction hetero atom or water molecules at the proteins binding site [137]. The MM-GBSA derived BFE of the docking complex was calculated to validate the affinity of the compounds to the receptor protein that was obtained from the docking study. MM-GBSA for a P-L complex calculated using a combination of molecular mechanics and continuum solvent models. The average BFE for the three complex ACE2_Silibinin, S_Beta-amyrin, and MAPK8_Taraxerol −38.27, −24.67, and −22.84 kcal/mol. Thus, as we mentioned before, the higher the negative value of binding energy means better binding to the protein pocket, whereas positive energy means not no binding to the target [140]. Therefore, silibinin and beta amyrin shows highest negative BFE to their target proteins cavity.

To validate top-ranked three drug molecules (Silibinin, Taraxerol, and Beta-amyrin) computationally, we investigated their ADME, toxicity, and drug bioavailability properties, suggesting their promising pharmacokinetics profile [141]. The ADME analysis of Silibinin, Taraxerol, and Beta Amyrin reveals promising properties and notable challenges for their potential use as therapeutic agents for SARS-CoV-2. All three compounds exhibit proper Caco-2 permeability, indicating their potential for adequate intestinal absorption, which is a critical factor for oral drug bioavailability (Wang et al., 2015). The human intestinal absorption (HIA) values, suggesting that all the compounds highly absorbed by the HIA and they are efficiently taken up by the human intestine. None of the compounds can cross the BBB, minimizing potential CNS-related side effects, which is particularly advantageous for drugs targeting peripheral systems rather than central nervous system diseases. Regarding distribution, Silibinin has a low volume of distribution (VDs), while Taraxerol and Beta Amyrin show moderate distribution, enhancing their ability to target tissues affected by SARS-CoV-2. Importantly, none of the compounds inhibit key cytochrome P450 enzymes (CYP1A2, CYP2C19, CYP3A4, CYP2D6), indicating a low risk of drug-drug interactions and predictable pharmacokinetics, an essential consideration given the polypharmacy commonly seen in COVID-19 treatment regimens. However, their high clearance rates and very short half-lives suggest rapid elimination, necessitating frequent dosing to maintain therapeutic levels, which can be a significant limitation. For example, Remdesivir, an antiviral used in COVID-19 treatment, has a half-life of around 0.89 hours but is administered intravenously, allowing for controlled dosing in a clinical setting. Overall, while these compounds show promising absorption and distribution properties with minimal metabolic interaction risk, extending their half-lives through formulation strategies will be essential to enhance their practicality and effectiveness as SARS-CoV-2 therapeutic agents.

Furthermore, toxicity potential evaluation is important for avoiding inappropriate chemicals for subsequent drug screening in order to begin *in vitro* and *in vivo* evaluation [84]. The phytocomponents were investigated for toxicity class, skin sensitization, hepatotoxicity, AMES toxicity, and carcinogenicity. All three compounds are inactive in terms of carcinogenicity and cytotoxicity, indicating a favorable safety profile regarding cancer risk and general cell toxicity. So, these molecules could be use as drugs on the basis of their drug-likeness, ADME and Toxicity parameters. These findings highlight the importance of balancing efficacy with safety in drug development, and while these compounds show promising toxicity profiles, further in vivo and clinical studies are necessary to fully assess their safety and therapeutic potential.

We further performed the DFT calculation to evaluate the electronics properties and quantum chemical descriptors of the selected phytocompounds, to determine the distribution of electron density in distinct frontier molecular orbitals of molecules. A smaller energy gap (∆E) between the LUMO and HOMO energies has a considerable influence on molecules’ intermolecular charge transfer and bioactivity [142], hence the reactivity decreases when the ∆E increases [133]. Thus, the decreasing order of the ∆E value according to the following trend: beta-amyrin (0.2488 Ha)> taraxerol (0.2465 Ha)> silibinin (0. 1382 Ha). Hence, the reactivity order increases according to beta-amyrin > taraxerol > silibinin where the most reactive is clearly silibinin [94]. A higher electron affinity value suggests that the molecule has a greater tendency to accept electrons, which may affect its interactions with other molecules or biological systems. Electrophilicity (ω) indicates a molecule’s tendency to take an electron, with high values of indicating good electrophilicity in a molecule. It can be observed that, the silibinin has the highest electrophilicity (0.14189 Ha) among the three compounds, indicating a higher tendency to accept electrons compared to taraxerol and beta-amyrin. This higher electrophilicity is due to its relatively higher electronegativity (0.14499 Ha) and lower chemical hardness (0.07408 Ha). The larger value of electrophilicity suggests that Silibinin can act more effectively as an electrophile in chemical reactions, making it potentially more reactive in biological environment where electron acceptance is important and drugs having a high electrophilic nature are known to have strong antibacterial and anticancer activities [143]. The MEP study shows areas that are prone to electrophilic and nucleophilic attacks; collectively, these studies offer a deep understanding of the chemical behaviors of these compounds. The intriguing properties of silibinin, beta-amyrin, and taraxerol, such as the electronic structures and reaction profiles found through DFT analysis, as well as their MEP-derived potential for specific biochemical interactions, make them very promising for treating SARS-CoV-2. Based on these analyzed results we hypothesized that the suggested three phytocompound (Silibinin, Taraxerol, and Beta amyrin) might be act as potential anti-SARS-CoV-2 agents. Therefore, based on the above discussion and results interpretations it may be conclude that our suggested phytocompounds might be a source of effective treatment against SARS-CoV-2 infections, however, the findings require experimental validation.

## 5. Conclusion

To explore potential phytocompounds of black cumin as inhibitors against SARS-CoV-2 infections, we selected infection-causing top-ranked key genes/proteins as the receptors by the systematic literature review. Then we identified top-ranked three phytocompounds silibinin, taraxerol and beta-amyrin as the inhibitors of SARS-CoV-2 infections based on their average docking score (−8.38, −8.33, and −8.22 kcal/mol respectively) and MD simulation studies with the top-ranked P-L complexes indicate the stable performance of the drug binding to the protein’s cavity. The Drug likeness, ADME/T, medicinal chemistry parameters, and quantum chemical descriptors or DFT analysis results with those top-ranked phytocompounds indicated acceptable pharmacokinetics indicators and non-toxic class. Therefore, the proposed phytocompounds of black cumin might be useful resources to design effective non-toxic therapeutics against SARS CoV-2 infections.

## 6. Limitations of the study

This study employed various *in-silico* analysis, including molecular docking, molecular dynamics (MD) simulations, BFE calculations, and pharmacokinetic property predictions, which are insightful studies. Although this study addressed promising hypothesized binding affinity by molecular docking, binding stability information using MD simulation and MM-GBSA calculations may not fully capture the complexity of biological interactions that occur *in-vivo* or *in vitro* studies. Also, we acknowledge the lack of mutagenesis study, as the SARS-CoV-2 genes associated with the infections are highly mutated, so it is required to consider such kind of issues to emphasize the problems of drug resistance. Additionally, the analyzed pharmacokinetic predictions depend on computational models that require experimental validation to prove the selectivity and efficacy of the selected BC derived phytocompounds. To confirm the therapeutic potential of these compounds, further *in-vitro* and *in-vivo* studies are essential to evaluate their efficacy, bioavailability, and potential off-target effects in determining the clinical relevance of these phytochemicals as potential therapeutic agents against SARS-CoV-2.

## Supporting information

S1 Fig Molecular interaction of rest of the top ranked compounds (Folic acid & 24-Methylenelophenol with corresponding target protein. (DOCX)

S2 Fig Molecular interaction between reference drugs (ACE2_ ritonavir; MAPK8_ nirmatrelvir; S_Remdesivir) and top-ranked phytocompounds with top-ranked target protein. (DOCX)

S1 Table List of key genes/proteins/proteases sets associated with SARS CoV-2 infection by the literature review. (DOCX)

S2 Table Top-ranked SARS-CoV-2 infection causing key proteins/proteases highlighting their types, number of supporting articles and references. The number in the first bracket () indicates the number of supporting articles with Black Cumin.
(DOCX)

S3 Table Protein targets information’s and molecular docking parameters for re-docking study. (DOCX)

S4 Table Protein targets information’s and molecular docking parameters for site specific docking study. (DOCX)

S5 Table The physicochemical properties and drug-likeness properties of all selected BC derived phytocompounds. (DOCX)

S6 Table Ordered ligands/phytocompounds of black cumin based on the average of binding affinity scores (aBAS) across top-ranked 11 receptors, where the proposed top-ranked 3 phytocompounds with their BAS and aBAS were highlighted in green color, and the previously recommended major bioactive phytocompounds with their BAS and aBAS were highlighted in blue color. (DOCX)

## Abbreviations

BC: Black Cumin
SARS-CoV-2: Severe acute respiratory syndrome coronavirus 2
MD: Molecular Dynamics
MM-GBSA: Molecular Mechanics Generalized Born Surface Area
Da: Dalton
ADMET: Absorption, Distribution, Metabolism, Excretion and Toxicity
B3LYP: Three parameters of Lee Becke-Parr
BBB: Blood-brain barrier
CID: Compound identifier
CNS: Central nervous system
CYP1A2: Cytochrome P450 family 1 subfamily A member 2
CYP2C19: Cytochrome P450 family 2 subfamily C member 19
CYP2C9: Cytochrome P450 family 2 subfamily C member 9
CYP2D6: Cytochrome P450 family 2 subfamily D member 6
CYP3A4: Cytochrome P450 family 3 subfamily A member 4
DFT: Density functional theory
D: Dipole Moment
MW: Molecular Weight
PDB ID: Protein Data Bank identifier
PDBQT: Protein data bank, Partial charge, and Atom type
RCSB PDB: Research Collaboratory for Structural Bioinformatics Protein Data Bank
Rg: Radius of Gyration
RMSD: Root Mean Square Deviation
RMSF: Root Mean Square Fluctuation
SASA: Solvent-Accessible Surface Area
DCCM: Dynamic Cross-Correlation Matrix
PCA: Principal Component Analysis
FEL: Free Energy Landscape
SDF: Structured data format
IMPPAT: Indian Medicinal Plants, Phytochemistry And Therapeutics
2D: 2 dimensional
3D: 3 dimensional
UFF: Universal force field
LOR5: Lipinski’s rule of 5
BAS: Binding Affinity Score
RNA: Ribonucleic acid
MERS-CoV: Middle east respiratory syndrome coronavirus
ARDS: Acute respiratory distress syndrome
LogP: The logarithm of the (water/octanol) partition coefficient
HubGs: Hub-genes
NVT: Constant number of particles, volume, and temperature
NPT: Constant number of particles, pressure, and temperature
RoG: Radius of Gyration
MM-GBSA: Molecular Mechanics–Generalized Born Surface Area
PL: Protein–ligand
ROA: Rate of oral absorption
Caco2: Cancer coli-2
BBB: Blood brain barrier
HIA: Human intestinal absorption
hERG: Human Ether-a-go-go Related Gene
MO: Molecular orbital
HOMO: Highest Occupied Molecular Orbital
LUMO: Lowest Unoccupied Molecular Orbital
SMILE: Simplified Molecular Input Line Entry System
VR: Veber rule
nHBD: Number of hydrogen bond donor
nHBA: Number of hydrogen bond acceptor
PSA: Polar surface area
RotB: Rotatable bond
FDA: Food and Drug Administration
BFE: Binding free energy
PAINS: Pan-Assay Interference Compounds
FMO: Frontier molecular orbital
MEP: Molecular Electrostatic Potential
