# Supplementary material for: Exploring potential phytocompounds from black cumin as drug molecules against SARS-CoV-2 infections through bioinformatics analysis
Source: PLoS One. 2026 Mar 11;21(3):e0337970. doi: 10.1371/journal.pone.0337970 (PMC12978503; doi:10.1371/journal.pone.0337970)
Supplement: S1 Fig — (DOCX) [file pone.0337970.s001.docx]

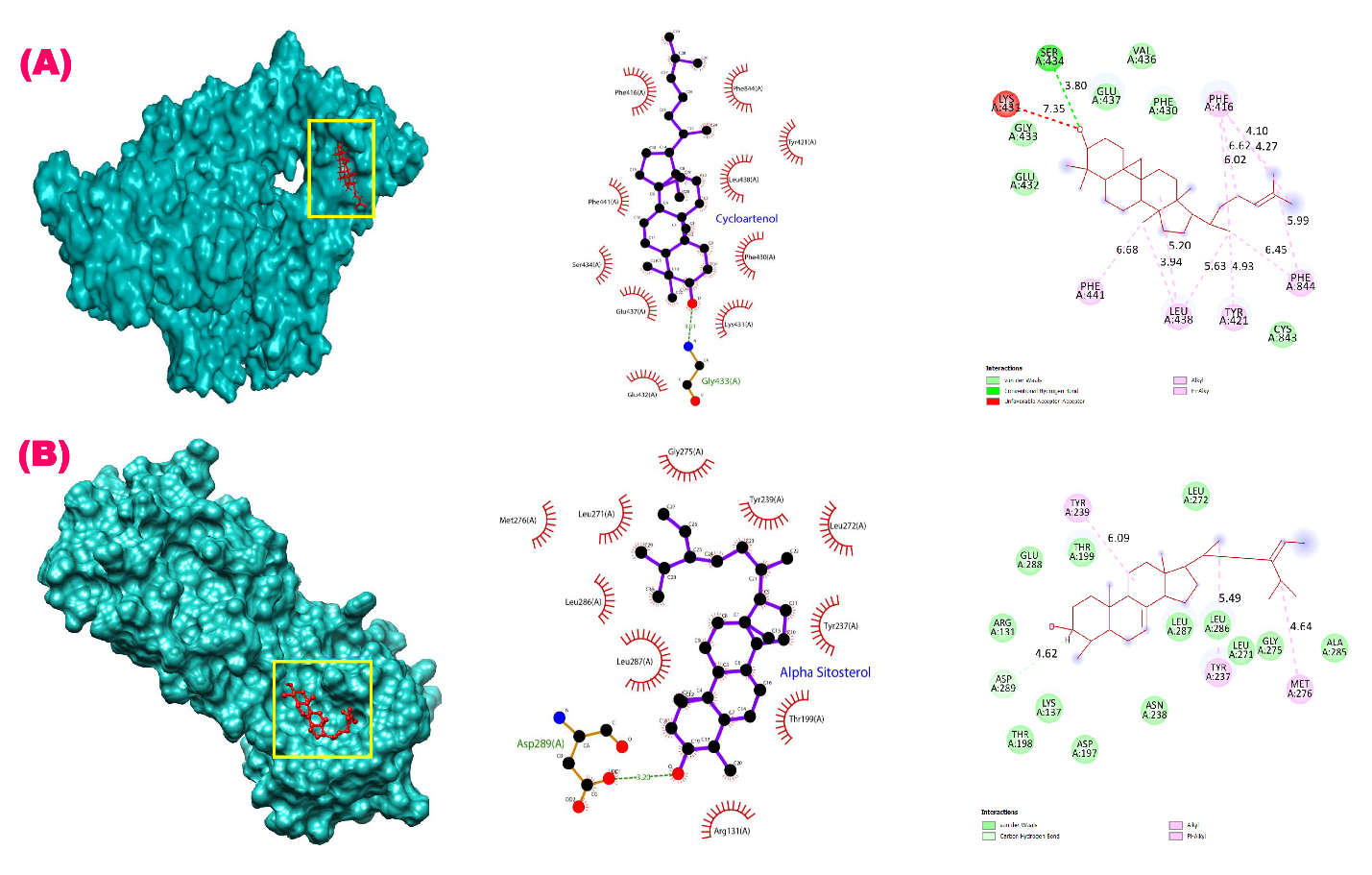


**S1 Fig**: Molecular interaction of rest of the top ranked compounds (Folic acid & 24-Methylenelophenol with corresponding target protein.
