# Supplementary material for: Exploring potential phytocompounds from black cumin as drug molecules against SARS-CoV-2 infections through bioinformatics analysis
Source: PLoS One. 2026 Mar 11;21(3):e0337970. doi: 10.1371/journal.pone.0337970 (PMC12978503; doi:10.1371/journal.pone.0337970)
Supplement: S2 Fig — (DOCX) [file pone.0337970.s002.docx]

**
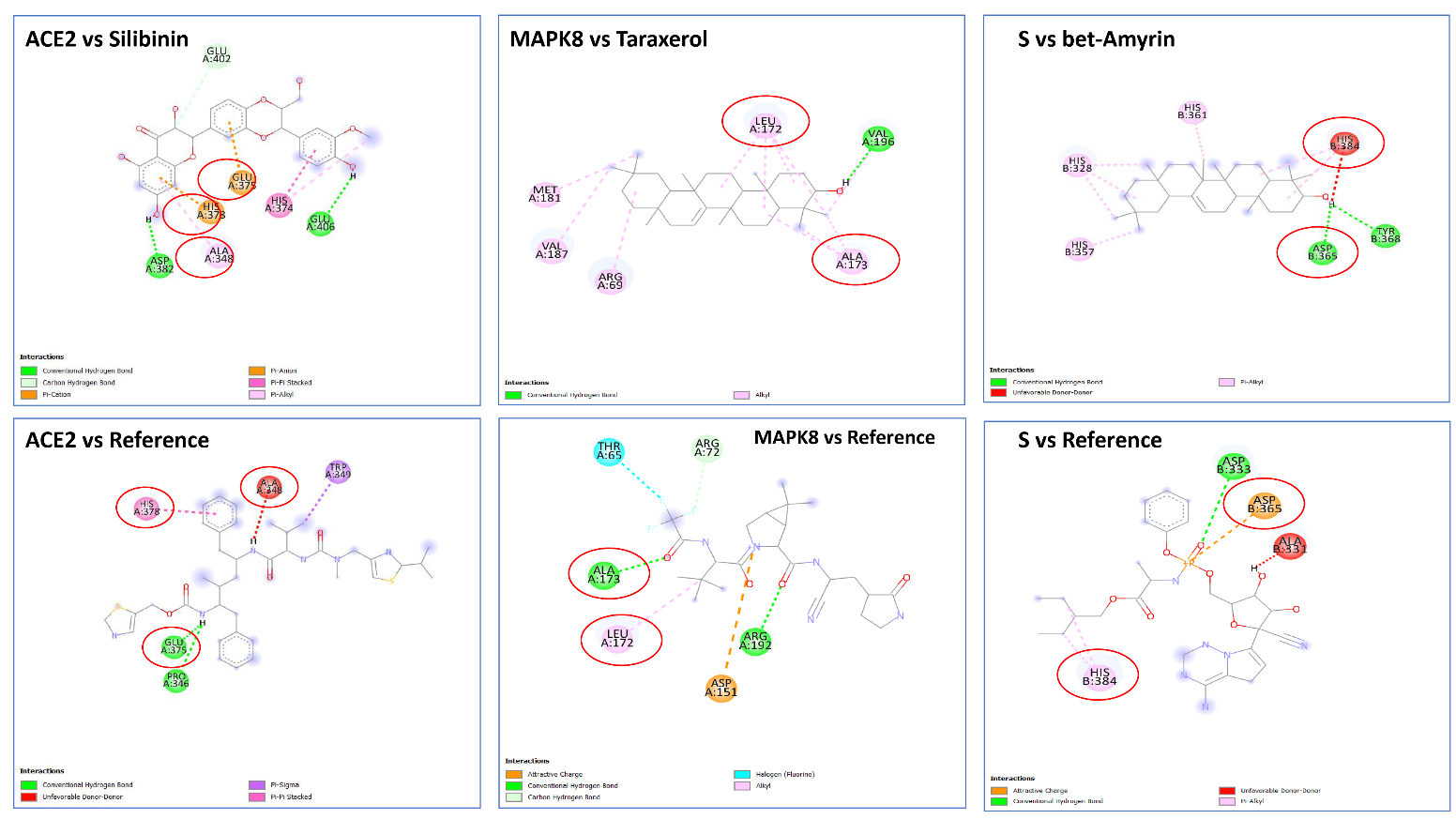
**

**S2 Fig:** Molecular interaction between reference drugs (ACE2_ ritonavir; MAPK8_ nirmatrelvir; S_Remdesivir) and top-ranked phytocompounds with top-ranked target protein.
