# Supplementary material for: Exploring potential phytocompounds from black cumin as drug molecules against SARS-CoV-2 infections through bioinformatics analysis
Source: PLoS One. 2026 Mar 11;21(3):e0337970. doi: 10.1371/journal.pone.0337970 (PMC12978503; doi:10.1371/journal.pone.0337970)
Supplement: S1 Table — (DOCX) [file pone.0337970.s003.docx]

**S1 Table:** List of key genes/proteins/proteases sets associated with SARS CoV-2 infection by the literature review

| **Article references** | SARS CoV-2 infection causing proteins/proteases |
| --- | --- |
| (S. A. Khan et al., 2021)[1] | 3CLpro |
| (Alves et al., 2021)[2] | M^pro^ |
| (Umesh et al., 2021)[3] | M^pro^ |
| (Liu et al., 2020)[4] | 3CLpro |
| (Aishwarya et al., 2020)[5] | M^pro^ |
| (Jin et al., 2020)[6] | M^pro^ |
| (Feng et al., 2021)[7] | 3CL ^Pro^ |
| (Günther et al., 2021)[8] | M^pro^ |
| (R. J. Khan et al., 2021)[9] | 3CLpro, 2'-O-MTase |
| (Raj, 2021)[10] | NSP-3, 5, 11, 14, 15 |
| (Kuo et al., 2021)[11] | 3CL^pro^, PL^pro^ |
| (Anand et al., 2021)[12] | NSP10, Nucleoprotein, NSP3, 3CLpro, |
| (Cavasotto and Di Filippo, 2021)[13] | M^pro^, PL^pro^, S-protein |
| (Gil et al., 2020)[14] | RNA polymerase, 3CLpro, PLpro, S |
| (Guedes et al., 2021)[15] | PLpro, Mpro, RdRp, N, S |
| (Liang et al., 2021)[16] | RdRp, 3CLpro, PLpro, S |
| (Rahman et al., 2021)[17] | M^pro^, RdRp, PL^pro^, S |
| (Murugan et al., 2020)⁠[18]⁠ | 3CLpro, Plpro, RdRp |
| (Manikyam and Joshi, 2020)⁠[19]⁠ | 3CLpro, Plpro,RdRp |
| (Wu et al., 2020)[20] | 3CLpro, PLpro, RdRp |
| (Beck et al., 2020)[21] | 3CL pro, RdRp, Helicase; 3’-to-5’ exonuclease; endoRNAse, 2ʹ-O-ribose methyltransferase |
| (Abdel-Basset et al., 2020)[22] | 3CLpro, RdRp |
| (Wang et al., 2021)[23] | NFκB1, CHUK, MAPK3, MAPK1, NFκB1A, CASP3, IL6, MAPK8, BAX, and TNF, TMPRSS2, ACE2, 3CLpro, RdRp, PLpro, Spike |
| (Mishra et al., 2021)[24] | S, hACE2, 3CLpro, CTSL, nucleocapsid protein, RdRp, NSP6 |
| (Nelakuditi and Shrivastava, 2020)[25] | Mpro, S, ACE2, RdRp |
| (Mhatre et al., 2021)⁠[26]⁠ | 3CLpro, S, PLpro, RdRp, ACE2 |
| (Joshi et al., 2020)[27] | SARS-CoV-2 M^Pro^, RdRp and hACE-2 |
| (Shi et al., 2021)[28] | ACE2, Mpro, RdRp |
| (Panda et al., 2020)[29] | SARS-CoV-2 Mpro, S, RBD, ACE2 |
| (Jena et al., 2021)[30] | ACE2, Mpro |
| (Tao et al., 2020)[31] | ACE2, 3CLpro |
| (Duverger et al., 2021)⁠[32]⁠ | ACE-2 |
| (Xiang et al., 2021)[33] | ACE2 |
| (Li and Yang, 2020)[34] | BALF, ACE2 |
| (Han et al., 2020)[35] | IL6, ACE2 |
| (de Oliveira et al., 2021)[36] | ACE2 |
| (Bardaweel et al., 2021)⁠[37]⁠ | DPP4, ACE2 |
| (Kabir et al., 2021)[38] | ACE2, TMPRSS2 |
| (Bojkova et al., 2020)  [39] | ACE2, RdRp |
| (Aftab et al., 2020)[40] | RdRp, ASP760, ASP761, |
| (Elfiky, 2021)[41] | RdRp |
| (Pirzada et al., 2021)[42] | RdRp |
| (Agrawal et al., 2021)[43] | S, RdRp |
| (Ruan et al., 2021)[44] | NSP12‐NSP7‐NSP8 |
| (Y. J. Sun et al., 2021)[45] | TMPRSS2 |
| (Cho et al., 2021)⁠[46]⁠ | TMPRSS2 |
| (Gao et al., 2021)[47] | PLpro |
| (Zhao et al., 2021)[48] | PLpro |
| (Weglarz-Tomczak et al., 2021)[49] | PL^pro^ |
| (Sinha et al., 2021)[50] | NSP15, S |
| (Yang et al., 2021)[51] | SARS-CoV-2 S protein |
| (Jeon et al., 2020)[52] | N protein |
| (Kumar et al., 2021)[53] | Noscapines protease |
| (C. Liu et al., 2021)[54] | NSP14 |
| (El Hassab et al., 2021)[55] | nsp16 |
| (F. Liu et al., 2021)[56] | AKT1, TP53, TNF, IL6, BCL2L, ATM |
| (G. Li et al., 2021)[57] | TNF |
| (Zhu et al., 2021)[58] | RELA, TNF, IL6, IL1B, MAPK14, TP53, CXCL8, MAPK3, MAPK1, IL4, MAPK8, CASP8, STAT1 |
| (Kumar, 2020)[59] | VEGFA, TNF, IL-6, CXCL8, IL10, CCL2, IL1B, TLR4, ICAM1, MMP9. |
| (Ge and He, 2020)[60] | MMP13, NLRP3, GBP1, ADORA2A, PTAFR, TNF, MLNR, IL1B, NFKBIA, ADRB2, IL6 |
| (Moni et al., 2020)[61] | MX1, IRF7, BST2 |
| (F. Sun et al., 2021)[62] | AKT1, AKT2 and AKT3 |
| (Dittmar et al., 2021)[63] | Cyclophilin |
| (Han et al., 2021)[64] | NFKBIA, IKBKB, CYP450 |
| (Y. Li et al., 2021)[65] | SRC, HDAC, MEK |
| (Sauvat et al., 2020)[66] | SARS-CoV-2 |
| (Krafcikova et al., 2020)[67] | 2′-O-RNA methyltransferase (MTase), nsp10-nsp16, RdRp, RNA cap |
| (Francis Borgio et al., 2020)[68] | SARS-CoV-2 helicase, |
| (Díaz, 2020)[69] | orf8, M, Nsp7, orf1b |
| (Gordon et al., 2020)[70] | SIGMAR1 |
| (Auwul et al., 2021)[71] | PLK1, AURKB, AURKA, CDK1, CDC20, KIF11, CCNB1, KIF2C, DTL, CDC6 |
| (Belyaeva et al., 2021)⁠[72]⁠ | HEK293T, ACAT1, ADK,AGA |
| (Lee et al., 2021)⁠[73]⁠ | SLC3A2, SLC2A3, FOLR2 |
| (Islam et al., 2020)⁠[74]⁠ | ICAM1, TNFAIP3 |
| (Desvaux et al., 2021)⁠[75]⁠ | ST2,  RAGE |
| (Nain et al., 2021)[76] | NFKBIA, BUB3, EIF2S3, GADD45A, MET, MCL1, SOCS3 |
| (Taz et al., 2020)[77] | SAA2, MMP9, SAA1, S100A8, ICAM1, PI3, SOD2, C8orf4, SERPINA3, S100A12, S100A9 |
| (Prasad et al., 2020)[78] | STAT1, IRF7, IFIH1, MX1, ISG15, IFIT3, OAS2, DDX58, IRF9, IFIT1, OAS1, OAS3, DDX60, OASL, IFIT2 |
| (O’Donovan et al., 2021)[79] | MEK inhibitor |
| (Yee et al., 2021)[80] | OATP2B1, OCT1, OCT2, OAT1, OAT3, MATE1, and MATE2 |
| (Zhou et al., 2020)⁠[81]⁠ | ORF1ab, ACE2, JUN, XPO1,  NPM1, HNRNPA1 |
| (Alam et al., 2021)[82]⁠ | hsa-miR-1307-3p, hsa-miR-1912-5p, hsa-miR-766-3p, hsa-miR-1910-5p, hsa-miR-1304-5p |
| Alanazi, Farah, and Hor 2022) [83] | NSP1, PLpro, Mpro, NSP9, RdRp, NSP13, NSP15, ORF3a, S, E, M, ORF6, ORF7a, N |
| (Jose et al. 2022)[84] | S |
| (Rahim et al. 2020) [85] | N, NSP2, Mpro, IL1, IL6 |
| (Xu et al. 2021) [86] | ACE2 |
| (Ruchi et al. 2020) [87] | ACE2 |
| (Ahmad et al. 2022) [88] | RdRp |
| (Duru, Duru, and Adegboyega 2021) [89] | rep-1a, NSP9, NSP3, 3CLpro, RdRp and ACE2– |
| (Maiti, Banerjee, and Kanwar 2020) [90] | ACE1, ACE2, AT1 and AT2 and Spike |
| (Esharkawy, Almalki, and Ben 2020) [91] | spike and envelopeproteins |
| (Khan et al. 2022) [92] | 3Clpro and NSP5 |
| [93] [93] | Spike & ACE2 |
| (Ullah et al. 2022) [94] | 3Clpro |
| (Siddiqui et al. 2022) [95] | Spike, NRBD, 3Clpro, PLpro |
| (Afroz et al. 2021) [96] | TGF-β1, 4 DPP-4, TNNI3K |
| (Baig and Srinivasan 2022) [97] | Nsp3.Nsp12, 3Clpro, Nsp13, Nsp15, Rep-1a, RdRp |
| (Choe, Har Yong, and Xiang Ng 2022) [98] | 3Clpro |
| (Salim and Noureddine 2020) [99] | 3Clpro |
| (Mir et al. 2022) [100] | RdRp |

**Reference:**

[1] S. A. Khan, K. Zia, S. Ashraf, R. Uddin, and Z. Ul-Haq, “Identification of chymotrypsin-like protease inhibitors of SARS-CoV-2 via integrated computational approach,” *J. Biomol. Struct. Dyn.*, vol. 39, no. 7, pp. 2607–2616, 2021, doi: 10.1080/07391102.2020.1751298.

[2] V. M. Alves *et al.*, “QSAR Modeling of SARS-CoV Mpro Inhibitors Identifies Sufugolix, Cenicriviroc, Proglumetacin, and other Drugs as Candidates for Repurposing against SARS-CoV-2,” *Mol. Inform.*, vol. 40, no. 1, 2021, doi: 10.1002/minf.202000113.

[3] Umesh, D. Kundu, C. Selvaraj, S. K. Singh, and V. K. Dubey, “Identification of new anti-nCoV drug chemical compounds from Indian spices exploiting SARS-CoV-2 main protease as target,” *J. Biomol. Struct. Dyn.*, vol. 39, no. 9, pp. 3428–3434, 2021, doi: 10.1080/07391102.2020.1763202.

[4] S. Liu, Q. Zheng, and Z. Wang, “Potential covalent drugs targeting the main protease of the SARS-CoV-2 coronavirus,” *Bioinformatics*, vol. 36, no. 11, 2020, doi: 10.1093/bioinformatics/btaa224.

[5] S. Aishwarya, K. Gunasekaran, and A. A. Margret, “Computational gene expression profiling in the exploration of biomarkers, non-coding functional RNAs and drug perturbagens for COVID-19,” *J. Biomol. Struct. Dyn.*, 2020, doi: 10.1080/07391102.2020.1850360.

[6] Z. Jin *et al.*, “Structure of Mpro from SARS-CoV-2 and discovery of its inhibitors,” *Nature*, vol. 582, no. 7811, pp. 289–293, 2020, doi: 10.1038/s41586-020-2223-y.

[7] Z. Feng *et al.*, “MCCS: a novel recognition pattern-based method for fast track discovery of anti-SARS-CoV-2 drugs,” *Brief. Bioinform.*, vol. 22, no. 2, pp. 946–962, 2021, doi: 10.1093/bib/bbaa260.

[8] S. Günther *et al.*, “X-ray screening identifies active site and allosteric inhibitors of SARS-CoV-2 main protease,” *Science (80-. ).*, vol. 372, no. 6542, 2021, doi: 10.1126/science.abf7945.

[9] R. J. Khan *et al.*, “Targeting SARS-CoV-2: a systematic drug repurposing approach to identify promising inhibitors against 3C-like proteinase and 2′-O-ribose methyltransferase,” *J. Biomol. Struct. Dyn.*, vol. 39, no. 8, pp. 2679–2692, 2021, doi: 10.1080/07391102.2020.1753577.

[10] R. Raj, “Analysis of non-structural proteins, NSPs of SARS-CoV-2 as targets for computational drug designing,” *Biochem. Biophys. Reports*, vol. 25, p. 100847, 2021, doi: 10.1016/j.bbrep.2020.100847.

[11] C. J. Kuo *et al.*, “Kinetic characterization and inhibitor screening for the proteases leading to identification of drugs against SARS-CoV-2,” *Antimicrob. Agents Chemother.*, vol. 65, no. 4, 2021, doi: 10.1128/AAC.02577-20.

[12] N. M. Anand *et al.*, “A comprehensive SARS-CoV-2 genomic analysis identifies potential targets for drug repurposing,” *PLoS One*, vol. 16, no. 3 March, 2021, doi: 10.1371/journal.pone.0248553.

[13] C. N. Cavasotto and J. I. Di Filippo, “In silico Drug Repurposing for COVID-19: Targeting SARS-CoV-2 Proteins through Docking and Consensus Ranking,” *Mol. Inform.*, vol. 40, no. 1, pp. 1–8, 2021, doi: 10.1002/minf.202000115.

[14] C. Gil *et al.*, “COVID-19: Drug Targets and Potential Treatments,” *J. Med. Chem.*, vol. 63, no. 21, pp. 12359–12386, 2020, doi: 10.1021/acs.jmedchem.0c00606.

[15] I. A. Guedes *et al.*, “Drug design and repurposing with DockThor-VS web server focusing on SARS-CoV-2 therapeutic targets and their non-synonym variants,” *Sci. Rep.*, vol. 11, no. 1, pp. 1–20, 2021, doi: 10.1038/s41598-021-84700-0.

[16] H. Liang, L. Zhao, X. Gong, M. Hu, and H. Wang, “Virtual screening FDA approved drugs against multiple targets of SARS-CoV-2,” *Clin. Transl. Sci.*, vol. 14, no. 3, pp. 1123–1132, 2021, doi: 10.1111/cts.13007.

[17] F. Rahman, S. Tabrez, R. Ali, A. S. Alqahtani, M. Z. Ahmed, and A. Rub, “Molecular docking analysis of rutin reveals possible inhibition of SARS-CoV-2 vital proteins,” *J. Tradit. Complement. Med.*, vol. 11, no. 2, pp. 173–179, 2021, doi: 10.1016/j.jtcme.2021.01.006.

[18] N. A. Murugan, S. Kumar, J. Jeyakanthan, and V. Srivastava, “Searching for target-specific and multi-targeting organics for Covid-19 in the Drugbank database with a double scoring approach,” *Sci. Rep.*, vol. 10, no. 1, 2020, doi: 10.1038/s41598-020-75762-7.

[19] H. K. Manikyam and S. K. Joshi, “Whole Genome Analysis and Targeted Drug Discovery Using Computational Methods and High Throughput Screening Tools for Emerged Novel Coronavirus (2019-nCoV).,” *J. Pharm. drug Res.*, vol. 3, no. 2, 2020.

[20] C. Wu *et al.*, “Analysis of therapeutic targets for SARS-CoV-2 and discovery of potential drugs by computational methods,” *Acta Pharm. Sin. B*, vol. 10, no. 5, pp. 766–788, 2020, doi: 10.1016/j.apsb.2020.02.008.

[21] B. R. Beck, B. Shin, Y. Choi, S. Park, and K. Kang, “Predicting commercially available antiviral drugs that may act on the novel coronavirus (SARS-CoV-2) through a drug-target interaction deep learning model,” *Comput. Struct. Biotechnol. J.*, 2020, doi: 10.1016/j.csbj.2020.03.025.

[22] M. Abdel-Basset, H. Hawash, M. Elhoseny, R. K. Chakrabortty, and M. Ryan, “Deeph-DTA: Deep learning for predicting drug-target interactions: A case study of covid-19 drug repurposing,” *IEEE Access*, vol. 8, pp. 170433–170451, 2020, doi: 10.1109/ACCESS.2020.3024238.

[23] Z. Z. Wang *et al.*, “A small molecule compound berberine as an orally active therapeutic candidate against COVID-19 and SARS: A computational and mechanistic study,” *FASEB J.*, vol. 35, no. 4, 2021, doi: 10.1096/fj.202001792R.

[24] C. B. Mishra *et al.*, “Identifying the natural polyphenol catechin as a multi-targeted agent against SARS-CoV-2 for the plausible therapy of COVID-19: An integrated computational approach,” *Brief. Bioinform.*, vol. 22, no. 2, pp. 1346–1360, 2021, doi: 10.1093/bib/bbaa378.

[25] B. Nelakuditi and A. Shrivastava, “Drug Repurposing 57 well-known drugs for three COVID-19 targets : Mpro , Spike , RdRp,” *Biol. Med. Chem.*, no. March 2020, 2020, doi: 10.33774/chemrxiv-2021-smfn8.

[26] S. Mhatre, S. Naik, and V. Patravale, “A molecular docking study of EGCG and theaflavin digallate with the druggable targets of SARS-CoV-2,” *Comput. Biol. Med.*, vol. 129, 2021, doi: 10.1016/j.compbiomed.2020.104137.

[27] R. S. Joshi *et al.*, “Discovery of potential multi-target-directed ligands by targeting host-specific SARS-CoV-2 structurally conserved main protease,” *J. Biomol. Struct. Dyn.*, vol. 39, no. 9, pp. 1–16, 2020, doi: 10.1080/07391102.2020.1760137.

[28] A. M. Shi, R. Guo, Q. Wang, and J. R. Zhou, “Screening and molecular modeling evaluation of food peptides to inhibit key targets of covid‐19 virus,” *Biomolecules*, vol. 11, no. 2, 2021, doi: 10.3390/biom11020330.

[29] P. K. Panda *et al.*, “Structure-based drug designing and immunoinformatics approach for SARS-CoV-2,” *Sci. Adv.*, vol. 6, no. 28, pp. 1–15, 2020, doi: 10.1126/sciadv.abb8097.

[30] S. Jena, P. Munusami, B. Mm, and K. Chanda, “Computationally approached inhibition potential of Tinospora cordifolia towards COVID-19 targets,” *VirusDisease*, vol. 32, no. 1, 2021, doi: 10.1007/s13337-021-00666-7.

[31] Q. Tao *et al.*, “Network pharmacology and molecular docking analysis on molecular targets and mechanisms of Huashi Baidu formula in the treatment of COVID-19,” *Drug Dev. Ind. Pharm.*, 2020, doi: 10.1080/03639045.2020.1788070.

[32] E. Duverger, G. Herlem, and F. Picaud, “A potential solution to avoid overdose of mixed drugs in the event of Covid-19: Nanomedicine at the heart of the Covid-19 pandemic,” *J. Mol. Graph. Model.*, vol. 104, 2021, doi: 10.1016/j.jmgm.2021.107834.

[33] and G. L. He, Xiang, JUNYI WANG, Lei Zhang, Qin Ran, Anying Xiong, Shengbin Liu, Dehong Wu, Bin Niu, Ying Xiong, “Virtual Screening of Potential AEC2 Inhibitors for COVID-19 from Traditional Chinese Medicine,” 2021, doi: https://doi.org/10.21203/rs.3.rs-145338/v1.

[34] Z. Li and L. Yang, “Underlying Mechanisms and Candidate Drugs for COVID-19 Based on the Connectivity Map Database,” *Front. Genet.*, vol. 11, 2020, doi: 10.3389/fgene.2020.558557.

[35] L. Han *et al.*, “Potential mechanism prediction of Cold-Damp Plague Formula against COVID-19 via network pharmacology analysis and molecular docking,” *Chinese Med. (United Kingdom)*, vol. 15, no. 1, 2020, doi: 10.1186/s13020-020-00360-8.

[36] O. V. de Oliveira, G. B. Rocha, A. S. Paluch, and L. T. Costa, “Repurposing approved drugs as inhibitors of SARS-CoV-2 S-protein from molecular modeling and virtual screening,” *J. Biomol. Struct. Dyn.*, vol. 39, no. 11, 2021, doi: 10.1080/07391102.2020.1772885.

[37] S. K. Bardaweel, R. Hajjo, and D. A. Sabbah, “Sitagliptin: A potential drug for the treatment of COVID-19?,” *Acta Pharm.*, vol. 71, no. 2, 2021, doi: 10.2478/acph-2021-0013.

[38] E. R. Kabir, N. Mustafa, N. Nausheen, M. K. Sharif Siam, and E. U. Syed, “Exploring existing drugs: proposing potential compounds in the treatment of COVID-19,” *Heliyon*, vol. 7, no. 2, 2021, doi: 10.1016/j.heliyon.2021.e06284.

[39] D. Bojkova *et al.*, “Proteomics of SARS-CoV-2-infected host cells reveals therapy targets,” *Nature*, vol. 583, no. 7816, pp. 469–472, 2020, doi: 10.1038/s41586-020-2332-7.

[40] S. O. Aftab *et al.*, “Analysis of SARS-CoV-2 RNA-dependent RNA polymerase as a potential therapeutic drug target using a computational approach,” *J. Transl. Med.*, vol. 18, no. 1, pp. 1–15, 2020, doi: 10.1186/s12967-020-02439-0.

[41] A. A. Elfiky, “SARS-CoV-2 RNA dependent RNA polymerase (RdRp) targeting: an in silico perspective,” *J. Biomol. Struct. Dyn.*, vol. 39, no. 9, pp. 3204–3212, 2021, doi: 10.1080/07391102.2020.1761882.

[42] R. H. Pirzada, M. Haseeb, M. Batool, M. S. Kim, and S. Choi, “Remdesivir and Ledipasvir among the FDA-Approved Antiviral Drugs Have Potential to Inhibit SARS-CoV-2 Replication,” *Cells*, vol. 10, no. 5, 2021, doi: 10.3390/cells10051052.

[43] L. Agrawal *et al.*, “Viroinformatics-Based Analysis of SARS-CoV-2 Core Proteins for Potential Therapeutic Targets,” *Antibodies*, vol. 10, no. 1, p. 3, 2021, doi: 10.3390/antib10010003.

[44] Z. Ruan *et al.*, “SARS-CoV-2 and SARS-CoV: Virtual screening of potential inhibitors targeting RNA-dependent RNA polymerase activity (NSP12),” *J. Med. Virol.*, vol. 93, no. 1, pp. 389–400, 2021, doi: 10.1002/jmv.26222.

[45] Y. J. Sun *et al.*, “Structure-based phylogeny identifies avoralstat as a TMPRSS2 inhibitor that prevents SARS-CoV-2 infection in mice,” *J. Clin. Invest.*, vol. 131, no. 10, 2021, doi: 10.1172/JCI147973.

[46] T. Cho, H. S. Han, J. Jeong, E. M. Park, and K. S. Shim, “A novel computational approach for the discovery of drug delivery system candidates for covid-19,” *Int. J. Mol. Sci.*, vol. 22, no. 6, 2021, doi: 10.3390/ijms22062815.

[47] X. Gao *et al.*, “Crystal structure of SARS-CoV-2 papain-like protease,” *Acta Pharm. Sin. B*, vol. 11, no. 1, pp. 237–245, 2021, doi: 10.1016/j.apsb.2020.08.014.

[48] Y. Zhao *et al.*, “High-throughput screening identifies established drugs as SARS-CoV-2 PLpro inhibitors,” *Protein Cell*, 2021, doi: 10.1007/s13238-021-00836-9.

[49] E. Weglarz-Tomczak, J. M. Tomczak, M. Talma, M. Burda-Grabowska, M. Giurg, and S. Brul, “Identification of ebselen and its analogues as potent covalent inhibitors of papain-like protease from SARS-CoV-2,” *Sci. Rep.*, vol. 11, no. 1, pp. 1–10, 2021, doi: 10.1038/s41598-021-83229-6.

[50] S. K. Sinha *et al.*, “An in-silico evaluation of different Saikosaponins for their potency against SARS-CoV-2 using NSP15 and fusion spike glycoprotein as targets,” *J. Biomol. Struct. Dyn.*, vol. 39, no. 9, pp. 3244–3255, 2021, doi: 10.1080/07391102.2020.1762741.

[51] L. Yang *et al.*, “Identification of SARS-CoV-2 entry inhibitors among already approved drugs,” *Acta Pharmacol. Sin.*, vol. 42, no. 8, pp. 1347–1353, 2021, doi: 10.1038/s41401-020-00556-6.

[52] S. Jeon *et al.*, “Identification of antiviral drug candidates against SARS-CoV-2 from FDA-approved drugs,” *Antimicrob. Agents Chemother.*, vol. 64, no. 7, pp. 1–9, 2020, doi: 10.1128/AAC.00819-20.

[53] D. Kumar *et al.*, “Understanding the binding affinity of noscapines with protease of SARS-CoV-2 for COVID-19 using MD simulations at different temperatures,” *J. Biomol. Struct. Dyn.*, vol. 39, no. 7, pp. 2659–2672, 2021, doi: 10.1080/07391102.2020.1752310.

[54] C. Liu, X. Zhu, Y. Lu, X. Zhang, X. Jia, and T. Yang, “Potential treatment with Chinese and Western medicine targeting NSP14 of SARS-CoV-2,” *J. Pharm. Anal.*, vol. 11, no. 3, pp. 272–277, 2021, doi: 10.1016/j.jpha.2020.08.002.

[55] M. A. El Hassab *et al.*, “In silico identification of potential SARS COV-2 2′- O -methyltransferase inhibitor: Fragment-based screening approach and MM-PBSA calculations,” *RSC Adv.*, vol. 11, no. 26, pp. 16026–16033, 2021, doi: 10.1039/d1ra01809d.

[56] F. Liu *et al.*, “Study on mechanism of matrine in treatment of COVID-19 combined with liver injury by network pharmacology and molecular docking technology,” *Drug Deliv.*, vol. 28, no. 1, 2021, doi: 10.1080/10717544.2021.1879313.

[57] G. Li, S. Ruan, X. Zhao, Q. Liu, Y. Dou, and F. Mao, “Transcriptomic signatures and repurposing drugs for COVID-19 patients: findings of bioinformatics analyses,” *Comput. Struct. Biotechnol. J.*, vol. 19, 2021, doi: 10.1016/j.csbj.2020.11.056.

[58] Y. W. Zhu *et al.*, “Analyzing the potential therapeutic mechanism of Huashi Baidu Decoction on severe COVID-19 through integrating network pharmacological methods,” *J. Tradit. Complement. Med.*, vol. 11, no. 2, 2021, doi: 10.1016/j.jtcme.2021.01.004.

[59] S. Kumar, “COVID-19: A drug repurposing and biomarker identification by using comprehensive gene-disease associations through protein-protein interaction network analysis,” *Preprints*, no. December 2019, 2020, doi: 10.20944/preprints202003.0440.v1.

[60] C. Ge and Y. He, “In Silico Prediction of Molecular Targets of Astragaloside IV for Alleviation of COVID-19 Hyperinflammation by Systems Network Pharmacology and Bioinformatic Gene Expression Analysis,” *Front. Pharmacol.*, vol. 11, 2020, doi: 10.3389/fphar.2020.556984.

[61] M. A. Moni, J. M. W. Quinn, N. Sinmaz, and M. A. Summers, “Gene expression profiling of SARS-CoV-2 infections reveal distinct primary lung cell and systemic immune infection responses that identify pathways relevant in COVID-19 disease,” *Brief. Bioinform.*, vol. 2020, no. 00, pp. 1–14, Dec. 2020, doi: 10.1093/bib/bbaa376.

[62] F. Sun *et al.*, “Capivasertib restricts SARS-CoV-2 cellular entry: a potential clinical application for COVID-19,” *Int. J. Biol. Sci.*, vol. 17, no. 9, pp. 2348–2355, 2021, doi: 10.7150/ijbs.57810.

[63] M. Dittmar *et al.*, “Drug repurposing screens reveal cell-type-specific entry pathways and FDA-approved drugs active against SARS-Cov-2,” *Cell Rep.*, vol. 35, no. 1, p. 108959, 2021, doi: 10.1016/j.celrep.2021.108959.

[64] N. Han *et al.*, “Identification of SARS-CoV-2–induced pathways reveals drug repurposing strategies,” *Sci. Adv.*, vol. 7, no. 27, pp. 1–15, 2021, doi: 10.1126/sciadv.abh3032.

[65] Y. Li *et al.*, “SARS-CoV-2 early infection signature identified potential key infection mechanisms and drug targets,” *BMC Genomics*, vol. 22, no. 1, pp. 1–13, 2021, doi: 10.1186/s12864-021-07433-4.

[66] A. Sauvat *et al.*, “On-target versus off-target effects of drugs inhibiting the replication of SARS-CoV-2,” *Cell Death Dis.*, vol. 11, no. 8, 2020, doi: 10.1038/s41419-020-02842-x.

[67] P. Krafcikova, J. Silhan, R. Nencka, and E. Boura, “Structural analysis of the SARS-CoV-2 methyltransferase complex involved in RNA cap creation bound to sinefungin,” *Nat. Commun.*, vol. 11, no. 1, pp. 1–7, 2020, doi: 10.1038/s41467-020-17495-9.

[68] J. Francis Borgio *et al.*, “State-of-the-art tools unveil potent drug targets amongst clinically approved drugs to inhibit helicase in SARS-CoV-2,” *Arch. Med. Sci.*, vol. 16, no. 2, pp. 508–518, 2020, doi: 10.5114/aoms.2020.94567.

[69] J. Díaz, “SARS-CoV-2 Molecular Network Structure,” *Front. Physiol.*, vol. 11, no. July, pp. 1–8, 2020, doi: 10.3389/fphys.2020.00870.

[70] D. E. Gordon *et al.*, “A SARS-CoV-2 protein interaction map reveals targets for drug repurposing,” *Nature*, vol. 583, no. 7816, 2020, doi: 10.1038/s41586-020-2286-9.

[71] M. R. Auwul, M. R. Rahman, E. Gov, M. Shahjaman, and M. A. Moni, “Bioinformatics and machine learning approach identifies potential drug targets and pathways in COVID-19,” *Brief. Bioinform.*, 2021, doi: 10.1093/bib/bbab120.

[72] A. Belyaeva *et al.*, “Causal network models of SARS-CoV-2 expression and aging to identify candidates for drug repurposing,” *Nat. Commun.*, vol. 12, no. 1, 2021, doi: 10.1038/s41467-021-21056-z.

[73] H. Lee, J. Park, H. J. Im, K. J. Na, and H. Choi, “Discovery of potential imaging and therapeutic targets for severe inflammation in COVID-19 patients,” *Sci. Rep.*, vol. 11, no. 1, 2021, doi: 10.1038/s41598-021-93743-2.

[74] T. Islam, M. R. Rahman, B. Aydin, H. Beklen, K. Y. Arga, and M. Shahjaman, “Integrative transcriptomics analysis of lung epithelial cells and identification of repurposable drug candidates for COVID-19,” *Eur. J. Pharmacol.*, vol. 887, 2020, doi: 10.1016/j.ejphar.2020.173594.

[75] E. Desvaux *et al.*, “Network-based repurposing identifies anti-alarmins as drug candidates to control severe lung inflammation in COVID-19,” *PLoS One*, vol. 16, no. 7 July, 2021, doi: 10.1371/journal.pone.0254374.

[76] Z. Nain, H. K. Rana, P. Liò, S. M. S. Islam, M. A. Summers, and M. A. Moni, “Pathogenetic profiling of COVID-19 and SARS-like viruses,” *Brief. Bioinform.*, vol. 22, no. 2, 2021, doi: 10.1093/bib/bbaa173.

[77] T. A. Taz *et al.*, “Network-based identification genetic effect of SARS-CoV-2 infections to Idiopathic pulmonary fibrosis (IPF) patients,” *Brief. Bioinform.*, vol. 00, no. August, pp. 1–13, 2020, doi: 10.1093/bib/bbaa235.

[78] K. Prasad *et al.*, “Targeting hub genes and pathways of innate immune response in COVID-19: A network biology perspective,” *Int. J. Biol. Macromol.*, vol. 163, 2020, doi: 10.1016/j.ijbiomac.2020.06.228.

[79] S. M. O’Donovan *et al.*, “Identification of candidate repurposable drugs to combat COVID-19 using a signature-based approach,” *Sci. Rep.*, vol. 11, no. 1, 2021, doi: 10.1038/s41598-021-84044-9.

[80] S. W. Yee *et al.*, “Drugs in COVID-19 Clinical Trials: Predicting Transporter-Mediated Drug-Drug Interactions Using In Vitro Assays and Real-World Data,” *Clin. Pharmacol. Ther.*, vol. 110, no. 1, 2021, doi: 10.1002/cpt.2236.

[81] Y. Zhou, Y. Hou, J. Shen, Y. Huang, W. Martin, and F. Cheng, “Network-based drug repurposing for novel coronavirus 2019-nCoV/SARS-CoV-2,” *Cell Discov.*, vol. 6, no. 1, 2020, doi: 10.1038/s41421-020-0153-3.

[82] T. Alam and L. Lipovich, “Mircovid-19: Potential targets of human mirnas in sars-cov-2 for rna-based drug discovery,” *Non-coding RNA*, vol. 7, no. 1, 2021, doi: 10.3390/NCRNA7010018.

[83] K. M. Alanazi, M. A. Farah, and Y. Y. Hor, “Multi-targeted approaches and drug repurposing reveal possible SARS-CoV-2 inhibitors,” *Vaccines*, vol. 10, no. 1, 2022, doi: 10.3390/vaccines10010024.

[84] S. Jose, M. Gupta, U. Sharma, J. Quintero-Saumeth, and M. Dwivedi, “Potential of phytocompounds from Brassica oleracea targeting S2-domain of SARS-CoV-2 spike glycoproteins: Structural and molecular insights,” *J. Mol. Struct.*, vol. 1254, 2022, doi: 10.1016/j.molstruc.2022.132369.

[85] M. Smarajit, B. Amrita, N. Aarifa, K. Mehak, and D. Shilpa, “Active-site Molecular docking of Nigellidine with nucleocapsid- NSP2-MPro of COVID-19 and to human IL1R-IL6R and strong antioxidant role of Nigella-sativa in experimental rats,” *J. Drug Target.*, vol. 0, no. 0, p. 000, 2020, doi: 10.1080/1061186X.2020.1817040.

[86] H. Xu, B. Liu, Z. Xiao, M. Zhou, and L. Ge, “Computational and Experimental Studies Reveal That Thymoquinone Blocks the Entry of Coronaviruses Into In Vitro Cells,” *Infect. Dis. Ther.*, vol. 10, no. 1, pp. 483–494, 2021, doi: 10.1007/s40121-021-00400-2.

[87] J. M. Ruchi, S. Nikita, D. Nitu, S. Shikha, and P. K. Deepshikha, “Deciphering underlying mechanism of Sars-CoV-2 infection in humans and revealing the therapeutic potential of bioactive constituents from Nigella sativa to combat COVID19 : in-silico study,” *J. Biomol. Struct. Dyn.*, vol. 0, no. 0, pp. 1–13, 2020, doi: 10.1080/07391102.2020.1839560.

[88] S. Ahmad *et al.*, “Identification of SARS-CoV-2 RNA-dependent RNA polymerase inhibitors from the major phytochemicals of Nigella sativa : An in silico approach,” *Saudi J. Biol. Sci.*, vol. 29, no. 1, pp. 394–401, 2022, doi: 10.1016/j.sjbs.2021.09.002.

[89] C. E. Duru, I. A. Duru, and A. E. Adegboyega, “In silico identification of compounds from Nigella sativa seed oil as potential inhibitors of SARS-CoV-2 targets,” *Bull. Natl. Res. Cent.*, vol. 45, no. 1, 2021, doi: 10.1186/s42269-021-00517-x.

[90] S. Maiti, A. Banerjee, and M. Kanwar, “In silico Nigellidine (N. sativa) bind to viral spike/active-sites of ACE1/2, AT1/2 to prevent COVID-19 induced vaso-tumult/vascular-damage/ comorbidity,” *Vascul. Pharmacol.*, vol. 138, no. January, p. 106856, 2020.

[91] E. R. Esharkawy, F. Almalki, and T. Ben, “In vitro potential antiviral SARS-CoV-19- activity of natural product thymohydroquinone and dithymoquinone from Nigella sativa,” *Bioorg. Chem.*, vol. 120, no. January, 2020, [Online]. Available: https://doi.org/10.1016/j.bioorg.2021.105587.

[92] M. T. Khan *et al.*, “Inhibitory effect of thymoquinone from Nigella sativa against SARS-CoV-2 main protease. An in-silico study,” *Brazilian J. Biol.*, vol. 84, pp. 1–7, 2024, doi: 10.1590/1519-6984.25066.

[93] S. Ahmad, H. W. Abbasi, S. Shahid, S. Gul, and S. W. Abbasi, “Molecular docking, simulation and MM-PBSA studies of nigella sativa compounds: a computational quest to identify potential natural antiviral for COVID-19 treatment,” *J. Biomol. Struct. Dyn.*, vol. 39, no. 12, pp. 4225–4233, 2021, doi: 10.1080/07391102.2020.1775129.

[94] S. Ullah *et al.*, “Identification of phytochemical inhibitors of SARS-CoV-2 protease 3CLpro from selected medicinal plants as per molecular docking, bond energies and amino acid binding energies,” *Saudi J. Biol. Sci.*, vol. 29, no. 6, p. 103274, 2022, doi: 10.1016/j.sjbs.2022.03.024.

[95] S. Siddiqui *et al.*, “Virtual screening of phytoconstituents from miracle herb nigella sativa targeting nucleocapsid protein and papain-like protease of SARS-CoV-2 for COVID-19 treatment,” *J. Biomol. Struct. Dyn.*, vol. 40, no. 9, pp. 3928–3948, 2022, doi: 10.1080/07391102.2020.1852117.

[96] S. Afroz, S. Fairuz, J. A. Joty, M. N. Uddin, and M. A. Rahman, “Virtual screening of functional foods and dissecting their roles in modulating gene functions to support post COVID-19 complications,” *Journal of Food Biochemistry*, vol. 45, no. 12. 2021, doi: 10.1111/jfbc.13961.

[97] A. Baig and H. Srinivasan, *SARS-CoV-2 Inhibitors from Nigella Sativa*, vol. 194, no. 3. Springer US, 2022.

[98] J. Choe, P. Har Yong, and Z. Xiang Ng, “The Efficacy of Traditional Medicinal Plants in Modulating the Main Protease of SARS-CoV-2 and Cytokine Storm,” *Chem. Biodivers.*, 2022, doi: 10.1002/cbdv.202200655.

[99] B. Salim and M. Noureddine, “Identification of Compounds from Nigella Sativa as New Potential Inhibitors of 2019 Novel Coronasvirus (Covid-19): Molecular Docking Study.,” *ChemRxiv*, vol. 19, pp. 1–12, 2020, [Online]. Available: https://chemrxiv.org/engage/chemrxiv/article-details/60c7495c469df4070af43bbf%0Ahttps://doi.org/10.26434/chemrxiv.12055716.v1.

[100] S. A. Mir *et al.*, “Identification of SARS-CoV-2 RNA-dependent RNA polymerase inhibitors from the major phytochemicals of Nigella sativa: An in silico approach,” *Saudi J. Biol. Sci.*, vol. 29, no. 1, pp. 394–401, 2022, doi: https://doi.org/10.1016/j.sjbs.2021.09.002.
