# Supplementary material for: Exploring potential phytocompounds from black cumin as drug molecules against SARS-CoV-2 infections through bioinformatics analysis
Source: PLoS One. 2026 Mar 11;21(3):e0337970. doi: 10.1371/journal.pone.0337970 (PMC12978503; doi:10.1371/journal.pone.0337970)
Supplement: S2 Table — The number in the first bracket () indicates the number of supporting articles with Black Cumin. (DOCX) [file pone.0337970.s004.docx]

**S2 Table:** Top-ranked SARS-CoV-2 infection causing key proteins/proteases highlighting their types, number of supporting articles and references. The number in the first bracket () indicates the number of supporting articles with Black Cumin.

| Key proteins/proteases | Sources | Number of supporting articles (with black cumin) | References of supporting articles |
| --- | --- | --- | --- |
| 3CLpro | Host | 40 (7) | [1]–[9], [11]–[31][83]–[85][89]  [92], [94], [95], [97]–[99] |
| PLpro | Host | 19 (2) | [23], [26], [47]–[49], [83], [89], [95][10]–[21] |
| RdRp | SARS CoV-2 | 26 (4) | [39]–[44], [15]–[28], [67], [88], [89], [97], [100] |
| S | SARS CoV-2 | 18 (5) | [13], [14], [43], [50], [51], [83], [84], [90], [91], [93], [15]–[17], [23]–[26], [29] |
| N | SARS CoV-2 | 5 (1) | [12], [15], [52], [83], [95] |
| ACE2 | Human | 22 (5) | [30]–[39], [82], [86], [87], [89], [90], [93] |
| MAPK8 | Human | 2 | [23], [58] |
| TMPRSS2 | Human | 4 | [23], [38], [45], [46] |
| IL6 | Human | 6 | [23], [35], [56], [58]–[60] |
| TNF | Human | 8 (1) | [23], [56]–[60], [74]⁠, [96] |
| NFKBIA | Human | 4 | [23], [60], [64], [76] |
