# Supplementary material for: Exploring potential phytocompounds from black cumin as drug molecules against SARS-CoV-2 infections through bioinformatics analysis
Source: PLoS One. 2026 Mar 11;21(3):e0337970. doi: 10.1371/journal.pone.0337970 (PMC12978503; doi:10.1371/journal.pone.0337970)
Supplement: S3 Table — (DOCX) [file pone.0337970.s005.docx]

**S3 Table:** Protein targets information’s and molecular docking parameters for re-docking study.

| Target | PDB ID | Resolution | Native Ligand | Docking Grid box coordinates | | | Docking Type | exhaustiveness | Number of Pose |
| --- | --- | --- | --- | --- | --- | --- | --- | --- | --- |
|  |  |  |  | Center_x | Center_y | Center_z |  |  |  |
| ACE2 | 2AJF | 2.90 | NAG130 | 29.3197 | -0.5012 | 48.2035 | Blind | 8 | 10 |
| Spike | 7T9K | 2.15 | NAG701 | 151.3841 | 124.9822 | 225.1277 | Blind | 8 | 10 |
| MAPK8 | 4HYU | 2.45 | 1BK401 | 9.8994 | 90.4006 | 130.9171 | Blind | 8 | 10 |
