# Supplementary material for: Exploring potential phytocompounds from black cumin as drug molecules against SARS-CoV-2 infections through bioinformatics analysis
Source: PLoS One. 2026 Mar 11;21(3):e0337970. doi: 10.1371/journal.pone.0337970 (PMC12978503; doi:10.1371/journal.pone.0337970)
Supplement: S4 Table — (DOCX) [file pone.0337970.s006.docx]

**S4 Table:** Protein targets information’s and molecular docking parameters for site specific docking study.

| Target | PDB ID | Resolution | Ligand | Docking Grid box coordinates | | | Docking Score | exhaustiveness | Number of Pose |
| --- | --- | --- | --- | --- | --- | --- | --- | --- | --- |
|  |  |  |  | Center_x | Center_y | Center_z |  |  |  |
| ACE2 | 2AJF | 2.90 | Silibinin | 27.959 | 5.457 | 60.569 | -7.3 | 8 | 10 |
|  |  |  | Ritonavir |  |  |  | -6.5 |  |  |
| Spike | 7T9K | 2.15 | Beta-amyrin | 152.752 | 125.700 | 230.574 | -8.8 | 8 | 10 |
|  |  |  | Remdesivir |  |  |  | -6.8 |  |  |
| MAPK8 | 4HYU | 2.45 | Taraxerol | 4.122 | 82.611 | 134.061 | -8.9 | 8 | 10 |
|  |  |  | Nirmatrelvir |  |  |  | -8.4 |  |  |
