# Supplementary material for: Exploring potential phytocompounds from black cumin as drug molecules against SARS-CoV-2 infections through bioinformatics analysis
Source: PLoS One. 2026 Mar 11;21(3):e0337970. doi: 10.1371/journal.pone.0337970 (PMC12978503; doi:10.1371/journal.pone.0337970)
Supplement: S5 Table — (DOCX) [file pone.0337970.s007.docx]

**S5 Table**: The physicochemical properties and drug-likeness properties of all selected BC derived phytocompounds

| **IMPPAT Phytochemical identifier** | **Phytochemical name** | **Physicochemical Properties** | | | | | |
| --- | --- | --- | --- | --- | --- | --- | --- |
|  |  | **MW** | **LogP** | **nHA** | **nHD** | **TPSA** | **nRot** |
| IMPHY000005 | Thiamine | 265.11 | -1.681 | 5 | 3 | 76.64 | 4 |
| IMPHY000099 | Myrtenol | 152.12 | 2.554 | 1 | 1 | 20.23 | 1 |
| IMPHY000108 | (3S,4S)-4-ethenyl-4-methyl-3-prop-1-en-2-ylcyclohexene | 162.14 | 4.053 | 0 | 0 | 0 | 2 |
| IMPHY000158 | Pyridoxine | 169.07 | -0.426 | 4 | 3 | 73.58 | 2 |
| IMPHY000399 | beta-Bisabolene | 204.19 | 6.252 | 0 | 0 | 0 | 4 |
| IMPHY000437 | Nigellidine | 294.14 | 3.832 | 4 | 1 | 43.78 | 0 |
| IMPHY000545 | O-Cymene | 134.11 | 3.838 | 0 | 0 | 0 | 1 |
| IMPHY000602 | M-Cymene | 134.11 | 3.946 | 0 | 0 | 0 | 1 |
| IMPHY000846 | Riboflavin | 376.14 | -0.379 | 10 | 5 | 161.56 | 5 |
| IMPHY001144 | Dillapiol | 222.09 | 2.501 | 4 | 0 | 36.92 | 4 |
| IMPHY001218 | Thymoquinone | 164.08 | 3.08 | 2 | 2 | 40.46 | 1 |
| IMPHY001246 | Carvacrol | 150.1 | 3.259 | 1 | 1 | 20.23 | 1 |
| IMPHY001516 | Decane | 142.17 | 5.668 | 0 | 0 | 0 | 7 |
| IMPHY001548 | Geranylacetone | 194.17 | 4.447 | 1 | 0 | 17.07 | 6 |
| IMPHY001658 | Thymol methyl ether | 164.12 | 3.753 | 1 | 0 | 9.23 | 2 |
| IMPHY001800 | Nigellicine | 246.1 | 0.846 | 5 | 1 | 64.23 | 1 |
| IMPHY001817 | p-Menth-3-en-1-ol | 154.14 | 2.696 | 1 | 1 | 20.23 | 1 |
| IMPHY001881 | 2-Tridecanone | 198.2 | 4.664 | 1 | 0 | 17.07 | 10 |
| IMPHY002053 | D-arabinonic acid | 166.05 | -2.273 | 6 | 5 | 118.22 | 4 |
| IMPHY002072 | Pinocarvone | 150.1 | 2.239 | 1 | 0 | 17.07 | 0 |
| IMPHY002139 | (-)-Butyrospermol | 426.39 | 8.445 | 1 | 1 | 20.23 | 4 |
| IMPHY002229 | Cycloeucalenol | 426.39 | 7.651 | 1 | 1 | 20.23 | 5 |
| IMPHY002825 | 2-(4-Methylphenyl)propan-2-ol | 150.1 | 2.354 | 1 | 1 | 20.23 | 1 |
| IMPHY003016 | Lauric acid | 200.18 | 4.793 | 2 | 1 | 37.3 | 10 |
| IMPHY003104 | Decanoic acid | 172.15 | 3.828 | 2 | 1 | 37.3 | 8 |
| IMPHY003398 | Myristicin | 192.08 | 2.665 | 3 | 0 | 27.69 | 3 |
| IMPHY003459 | Pimara-8(14),15-diene | 272.25 | 6.489 | 0 | 0 | 0 | 1 |
| IMPHY003467 | Dithymoquinone | 328.17 | 2.321 | 4 | 0 | 68.28 | 0 |
| IMPHY003482 | 4-Methoxybenzaldehyde | 136.05 | 1.623 | 2 | 0 | 26.3 | 2 |
| IMPHY003485 | Myrcene | 136.13 | 4.321 | 0 | 0 | 0 | 4 |
| IMPHY003490 | Coumarin | 146.04 | 1.672 | 2 | 0 | 30.21 | 0 |
| IMPHY003525 | Nonanal | 142.14 | 3.147 | 1 | 0 | 17.07 | 7 |
| IMPHY003536 | Eugenol | 164.08 | 2.291 | 2 | 1 | 29.46 | 3 |
| IMPHY003545 | 4-Isopropylbenzaldehyde | 148.09 | 2.887 | 1 | 0 | 17.07 | 2 |
| IMPHY003567 | alpha-Fenchene | 136.13 | 3.647 | 0 | 0 | 0 | 0 |
| IMPHY003695 | (-)-Germacrene A | 204.19 | 5.911 | 0 | 0 | 0 | 1 |
| IMPHY003723 | 2,4-Decadienal | 152.12 | 2.583 | 1 | 0 | 17.07 | 6 |
| IMPHY003915 | 2-Decenal | 154.14 | 3.352 | 1 | 0 | 17.07 | 7 |
| IMPHY003977 | (-)-beta-Bourbonene | 204.19 | 4.692 | 0 | 0 | 0 | 1 |
| IMPHY003982 | gamma-Terpinene | 136.13 | 4.307 | 0 | 0 | 0 | 1 |
| IMPHY004209 | Davanone D | 236.18 | 3.315 | 2 | 0 | 26.3 | 5 |
| IMPHY004215 | (1R,2R,7R,8R)-2,6,6,9-tetramethyltricyclo[5.4.0.02,8]undec-9-ene | 204.19 | 6.054 | 0 | 0 | 0 | 0 |
| IMPHY004216 | (1S,2S,7S,8S)-2,6,6,9-tetramethyltricyclo[5.4.0.02,8]undec-9-ene | 204.19 | 6.012 | 0 | 0 | 0 | 0 |
| IMPHY004286 | Longifolene | 204.19 | 5.175 | 0 | 0 | 0 | 0 |
| IMPHY004789 | 5-Dehydro-avenasterol | 410.35 | 6.492 | 1 | 1 | 20.23 | 5 |
| IMPHY005569 | alpha-Ionone | 192.15 | 3.535 | 1 | 0 | 17.07 | 2 |
| IMPHY005821 | gamma-Himachalene | 204.19 | 6.224 | 0 | 0 | 0 | 0 |
| IMPHY005974 | Longicyclene | 204.19 | 4.887 | 0 | 0 | 0 | 0 |
| IMPHY006145 | p-Cymene | 134.11 | 3.994 | 0 | 0 | 0 | 1 |
| IMPHY006163 | 2,2,5-Trimethyl-4-cyclohepten-1-one | 152.12 | 2.557 | 1 | 0 | 17.07 | 0 |
| IMPHY006177 | Methyl geranate | 182.13 | 3.145 | 2 | 0 | 26.3 | 5 |
| IMPHY006243 | 2,5-Dimethoxy-p-cymene | 194.13 | 3.778 | 2 | 0 | 18.46 | 3 |
| IMPHY006300 | Cholesterol | 386.35 | 7.651 | 1 | 1 | 20.23 | 5 |
| IMPHY006325 | Myrtenal | 150.1 | 2.544 | 1 | 0 | 17.07 | 1 |
| IMPHY006362 | Ascorbic acid | 176.03 | -1.42 | 6 | 5 | 114.29 | 2 |
| IMPHY006550 | Thymol | 150.1 | 3.153 | 1 | 1 | 20.23 | 1 |
| IMPHY006659 | Obtusifoliol | 426.39 | 7.275 | 1 | 1 | 20.23 | 5 |
| IMPHY006678 | 4-Acetyl-1,4-dimethyl-1-cyclohexene | 152.12 | 2.47 | 1 | 0 | 17.07 | 1 |
| IMPHY006696 | Methyleugenol | 178.1 | 2.54 | 2 | 0 | 18.46 | 4 |
| IMPHY006709 | Acetyleugenol | 206.09 | 2.28 | 3 | 0 | 35.53 | 5 |
| IMPHY006944 | Estragole | 148.09 | 2.99 | 1 | 0 | 9.23 | 3 |
| IMPHY006947 | 1-Decanol | 158.17 | 3.892 | 1 | 1 | 20.23 | 8 |
| IMPHY006950 | Tricyclene | 136.13 | 3.828 | 0 | 0 | 0 | 0 |
| IMPHY007067 | Linalyl acetate | 196.15 | 3.295 | 2 | 0 | 26.3 | 6 |
| IMPHY007202 | Nonane | 128.16 | 5.147 | 0 | 0 | 0 | 6 |
| IMPHY007204 | Dodecanal | 184.18 | 4.42 | 1 | 0 | 17.07 | 10 |
| IMPHY007224 | Hederagenin | 472.36 | 5.096 | 4 | 3 | 77.76 | 2 |
| IMPHY007286 | Umbellulon | 150.1 | 2.284 | 1 | 0 | 17.07 | 1 |
| IMPHY007357 | Nicotinic acid | 123.03 | 0.405 | 3 | 1 | 50.19 | 1 |
| IMPHY007421 | Citronellyl acetate | 198.16 | 4.192 | 2 | 0 | 26.3 | 7 |
| IMPHY007606 | Thymohydroquinone | 166.1 | 2.486 | 2 | 2 | 40.46 | 1 |
| IMPHY007840 | Spathulenol | 220.18 | 4.223 | 1 | 1 | 20.23 | 0 |
| IMPHY008150 | 1-Methyl-4-(prop-1-en-2-yl)benzene | 132.09 | 3.904 | 0 | 0 | 0 | 1 |
| IMPHY008162 | Citronellyl butyrate | 226.19 | 4.931 | 2 | 0 | 26.3 | 9 |
| IMPHY008451 | Isolongifolene | 204.19 | 5.544 | 0 | 0 | 0 | 0 |
| IMPHY008483 | 6,7-Dimethoxy-1-methylisoquinoline | 203.09 | 2.066 | 3 | 0 | 31.35 | 2 |
| IMPHY008729 | Lophenol | 400.37 | 8.014 | 1 | 1 | 20.23 | 5 |
| IMPHY008933 | 24-Ethyllophenol | 428.4 | 7.732 | 1 | 1 | 20.23 | 6 |
| IMPHY009737 | beta-Longipinene | 204.19 | 5.213 | 0 | 0 | 0 | 0 |
| IMPHY009752 | beta-Cyclocitral | 152.12 | 3.239 | 1 | 0 | 17.07 | 1 |
| IMPHY009764 | 2,10-Epoxypinane | 152.12 | 2.985 | 1 | 0 | 12.53 | 0 |
| IMPHY009840 | Cyclosativene | 204.19 | 4.148 | 0 | 0 | 0 | 1 |
| IMPHY009865 | alpha,3-Dimethylstyrene | 132.09 | 3.811 | 0 | 0 | 0 | 1 |
| IMPHY009866 | 2-(4-Methylphenyl)propan-1-ol | 150.1 | 2.376 | 1 | 1 | 20.23 | 2 |
| IMPHY009871 | Isoterpinolene | 136.13 | 3.895 | 0 | 0 | 0 | 0 |
| IMPHY009946 | Benzaldehyde | 106.04 | 1.495 | 1 | 0 | 17.07 | 1 |
| IMPHY010072 | Eucalyptol | 154.14 | 2.582 | 1 | 0 | 9.23 | 0 |
| IMPHY010080 | beta-Elemene | 204.19 | 4.998 | 0 | 0 | 0 | 3 |
| IMPHY010609 | (-)-alpha-Himachalene | 204.19 | 5.919 | 0 | 0 | 0 | 0 |
| IMPHY010712 | Nigellimine n-oxide | 219.09 | 0.691 | 4 | 0 | 45.4 | 2 |
| IMPHY010781 | Limonene oxide, cis-(-)- | 152.12 | 3.251 | 1 | 0 | 12.53 | 1 |
| IMPHY010815 | Thujopsene | 204.19 | 5.537 | 0 | 0 | 0 | 0 |
| IMPHY011004 | p-Mentha-1,3,8-triene | 134.11 | 3.158 | 0 | 0 | 0 | 1 |
| IMPHY011354 | trans-Sabinene hydrate acetate | 196.15 | 2.483 | 2 | 0 | 26.3 | 3 |
| IMPHY011371 | cis-Chrysanthenyl acetate | 194.13 | 3.559 | 2 | 0 | 26.3 | 2 |
| IMPHY011393 | 24-Methylenecycloartanol | 440.4 | 7.904 | 1 | 1 | 20.23 | 5 |
| IMPHY011396 | 4-Carvomenthenol | 154.14 | 3.06 | 1 | 1 | 20.23 | 1 |
| IMPHY011521 | 2-Undecanone | 170.17 | 3.815 | 1 | 0 | 17.07 | 8 |
| IMPHY011542 | beta-Eudesmol | 222.2 | 3.943 | 1 | 1 | 20.23 | 1 |
| IMPHY011545 | delta7-Avenasterol | 412.37 | 7.247 | 1 | 1 | 20.23 | 5 |
| IMPHY011552 | (1R)-2-methyl-5-propan-2-ylbicyclo[3.1.0]hex-2-ene | 136.13 | 3.907 | 0 | 0 | 0 | 1 |
| IMPHY011557 | 4-Isopropylbenzyl alcohol | 150.1 | 2.41 | 1 | 1 | 20.23 | 2 |
| IMPHY011558 | Apiole | 222.09 | 2.472 | 4 | 0 | 36.92 | 4 |
| IMPHY011570 | (2Z,6E)-Farnesyl acetate | 264.21 | 6.603 | 2 | 0 | 26.3 | 9 |
| IMPHY011576 | alpha-Spinasterol | 412.37 | 7.492 | 1 | 1 | 20.23 | 5 |
| IMPHY011581 | alpha-Selinene | 204.19 | 5.19 | 0 | 0 | 0 | 1 |
| IMPHY011589 | 7-epi-alpha-Eudesmol | 222.2 | 4.506 | 1 | 1 | 20.23 | 1 |
| IMPHY011590 | d-Borneol | 154.14 | 2.806 | 1 | 1 | 20.23 | 0 |
| IMPHY011599 | Terpinolene | 136.13 | 4.409 | 0 | 0 | 0 | 0 |
| IMPHY011631 | (E,Z)-farnesol | 222.2 | 4.263 | 1 | 1 | 20.23 | 7 |
| IMPHY011632 | Farnesol | 222.2 | 4.701 | 1 | 1 | 20.23 | 7 |
| IMPHY011642 | Cycloartenol | 426.39 | 8.377 | 1 | 1 | 20.23 | 4 |
| IMPHY011643 | alpha-Terpinene | 136.13 | 3.092 | 0 | 0 | 0 | 1 |
| IMPHY011648 | Neryl acetate | 196.15 | 3.253 | 2 | 0 | 26.3 | 6 |
| IMPHY011654 | alpha1-Sitosterol | 426.39 | 7.558 | 1 | 1 | 20.23 | 5 |
| IMPHY011658 | beta-Farnesene | 204.19 | 5.663 | 0 | 0 | 0 | 7 |
| IMPHY011677 | Taraxerol | 426.39 | 6.782 | 1 | 1 | 20.23 | 0 |
| IMPHY011703 | Silibinin | 482.12 | 2.21 | 10 | 5 | 155.14 | 4 |
| IMPHY011709 | alpha-Eudesmol | 222.2 | 4.494 | 1 | 1 | 20.23 | 1 |
| IMPHY011745 | Zingiberene | 204.19 | 5.703 | 0 | 0 | 0 | 4 |
| IMPHY011761 | Humulene | 204.19 | 5.795 | 0 | 0 | 0 | 0 |
| IMPHY011763 | Anethole | 148.09 | 2.572 | 1 | 0 | 9.23 | 2 |
| IMPHY011777 | Farnesyl acetate | 264.21 | 5.975 | 2 | 0 | 26.3 | 9 |
| IMPHY011789 | Citral | 152.12 | 2.792 | 1 | 0 | 17.07 | 4 |
| IMPHY011793 | (+)-gamma-Cadinene | 204.19 | 5.517 | 0 | 0 | 0 | 1 |
| IMPHY011839 | (Z)-gamma-bisabolene | 204.19 | 5.913 | 0 | 0 | 0 | 3 |
| IMPHY011855 | Tirucallol | 426.39 | 8.133 | 1 | 1 | 20.23 | 4 |
| IMPHY011882 | Cinnamaldehyde | 132.06 | 1.755 | 1 | 0 | 17.07 | 2 |
| IMPHY011884 | Pulegone | 152.12 | 2.803 | 1 | 0 | 17.07 | 0 |
| IMPHY011894 | (-)-7-Epi-alpha-selinene | 204.19 | 5.304 | 0 | 0 | 0 | 1 |
| IMPHY011957 | (+)-delta-Cadinene | 204.19 | 5.629 | 0 | 0 | 0 | 1 |
| IMPHY011973 | (-)-cis-Carveol | 152.12 | 2.478 | 1 | 1 | 20.23 | 1 |
| IMPHY011988 | (-)-trans-Carveol | 152.12 | 2.709 | 1 | 1 | 20.23 | 1 |
| IMPHY012002 | (+)-Dihydrocarvone | 152.12 | 2.264 | 1 | 0 | 17.07 | 1 |
| IMPHY012003 | Betulinic acid | 456.36 | 5.867 | 3 | 2 | 57.53 | 2 |
| IMPHY012036 | Camphor | 152.12 | 2.324 | 1 | 0 | 17.07 | 0 |
| IMPHY012058 | Linalool | 154.14 | 2.978 | 1 | 1 | 20.23 | 4 |
| IMPHY012061 | alpha-Pinene | 136.13 | 4.125 | 0 | 0 | 0 | 0 |
| IMPHY012075 | Carvone | 150.1 | 2.136 | 1 | 0 | 17.07 | 1 |
| IMPHY012082 | Gamma-nonalactone | 156.12 | 2.822 | 2 | 0 | 26.3 | 4 |
| IMPHY012144 | 1-Methyl-3-propylbenzene | 134.11 | 4.099 | 0 | 0 | 0 | 2 |
| IMPHY012147 | beta-Pinene | 136.13 | 3.625 | 0 | 0 | 0 | 0 |
| IMPHY012160 | alpha-Terpineol | 154.14 | 3.084 | 1 | 1 | 20.23 | 1 |
| IMPHY012165 | Sabinene | 136.13 | 3.274 | 0 | 0 | 0 | 1 |
| IMPHY012175 | D-Limonene | 136.13 | 4.446 | 0 | 0 | 0 | 1 |
| IMPHY012205 | Sabinene hydrate | 154.14 | 2.44 | 1 | 1 | 20.23 | 1 |
| IMPHY012223 | beta-Amyrin | 426.39 | 7.713 | 1 | 1 | 20.23 | 0 |
| IMPHY012255 | (+)-trans-Piperitenol | 154.14 | 3.009 | 1 | 1 | 20.23 | 1 |
| IMPHY012279 | alpha-Curcumene | 202.17 | 5.9 | 0 | 0 | 0 | 4 |
| IMPHY012402 | Campesterol | 400.37 | 7.735 | 1 | 1 | 20.23 | 5 |
| IMPHY012638 | Epizonarene | 204.19 | 4.637 | 0 | 0 | 0 | 1 |
| IMPHY012654 | Nerol | 154.14 | 2.518 | 1 | 1 | 20.23 | 4 |
| IMPHY012665 | Levomenol | 222.2 | 5.781 | 1 | 1 | 20.23 | 4 |
| IMPHY012737 | (1S,4E,9S)-4,11,11-trimethyl-8-methylidenebicyclo[7.2.0]undec-4-ene | 204.19 | 6.138 | 0 | 0 | 0 | 0 |
| IMPHY012738 | Isocaryophyllene | 204.19 | 4.95 | 0 | 0 | 0 | 0 |
| IMPHY012739 | (Z)-beta-Ocimene | 136.13 | 4.41 | 0 | 0 | 0 | 3 |
| IMPHY012769 | 24-Methylenelophenol | 412.37 | 7.529 | 1 | 1 | 20.23 | 5 |
| IMPHY012921 | gamma-Elemene | 204.19 | 5.126 | 0 | 0 | 0 | 2 |
| IMPHY013080 | alpha-Calacorene | 200.16 | 5.283 | 0 | 0 | 0 | 1 |
| IMPHY013764 | (-)-Carvomenthone | 154.14 | 2.548 | 1 | 0 | 17.07 | 1 |
| IMPHY013782 | 1-Ethyl-2,3-dimethylbenzene | 134.11 | 4.069 | 0 | 0 | 0 | 1 |
| IMPHY013804 | Octyl isobutyrate | 200.18 | 4.583 | 2 | 0 | 26.3 | 9 |
| IMPHY013836 | Fenchone | 152.12 | 2.338 | 1 | 0 | 17.07 | 0 |
| IMPHY013838 | 3,7-Dimethyloct-6-en-3-ol | 156.15 | 3.252 | 1 | 1 | 20.23 | 4 |
| IMPHY013977 | p-Mentha-1,5,8-triene | 134.11 | 3.483 | 0 | 0 | 0 | 1 |
| IMPHY014207 | (2E,4E)-3,7-dimethylocta-2,4,6-trienal | 150.1 | 1.744 | 1 | 0 | 17.07 | 3 |
| IMPHY014219 | 1,2-Dihydronaphthalen-2-one | 144.06 | 2.762 | 1 | 1 | 20.23 | 0 |
| IMPHY014249 | Naphthalen-1(2h)-one | 144.06 | 2.797 | 1 | 1 | 20.23 | 0 |
| IMPHY014513 | Longiborneol acetate | 264.21 | 4.935 | 2 | 0 | 26.3 | 2 |
| IMPHY014708 | beta-Selinene | 204.19 | 4.757 | 0 | 0 | 0 | 1 |
| IMPHY014749 | (E,Z)-2,4-Decadienal | 152.12 | 2.583 | 1 | 0 | 17.07 | 6 |
| IMPHY014806 | Caswell No. 264AB | 204.19 | 5.156 | 0 | 0 | 0 | 1 |
| IMPHY014811 | alpha-Phellandrene | 136.13 | 3.857 | 0 | 0 | 0 | 1 |
| IMPHY014817 | Aromadendrene | 204.19 | 4.585 | 0 | 0 | 0 | 0 |
| IMPHY014831 | beta-Caryophyllene | 204.19 | 6.057 | 0 | 0 | 0 | 0 |
| IMPHY014835 | (E)-beta-ocimene | 136.13 | 3.404 | 0 | 0 | 0 | 3 |
| IMPHY014836 | beta-Sitosterol | 414.39 | 8.025 | 1 | 1 | 20.23 | 6 |
| IMPHY014842 | Stigmasterol | 412.37 | 7.5 | 1 | 1 | 20.23 | 5 |
| IMPHY014847 | Bornyl acetate | 196.15 | 3.263 | 2 | 0 | 26.3 | 2 |
| IMPHY014852 | Camphene | 136.13 | 3.781 | 0 | 0 | 0 | 0 |
| IMPHY014866 | 2-Cyclohexen-1-ol, 2-methyl-5-(1-methylethenyl)-, acetate, cis- | 194.13 | 3.159 | 2 | 0 | 26.3 | 3 |
| IMPHY014872 | cis-Pinocarveol | 152.12 | 2.412 | 1 | 1 | 20.23 | 0 |
| IMPHY014873 | 2-Cyclohexen-1-ol, 3-methyl-6-(1-methylethyl)-, (1R,6S)-rel- | 154.14 | 2.654 | 1 | 1 | 20.23 | 1 |
| IMPHY014874 | cis-Sabinene hydrate | 138.14 | 3.77 | 0 | 0 | 0 | 1 |
| IMPHY014881 | Copaene | 204.19 | 4.844 | 0 | 0 | 0 | 1 |
| IMPHY014899 | Stigmastanol | 416.4 | 8.645 | 1 | 1 | 20.23 | 6 |
| IMPHY014907 | 6-Epi-beta-bisabolol | 222.2 | 5.199 | 1 | 1 | 20.23 | 4 |
| IMPHY014942 | Bicyclo[2.2.1]heptan-2-ol, 1,7,7-trimethyl-, formate, (1R,2R,4R)-rel- | 182.13 | 3.23 | 2 | 0 | 26.3 | 2 |
| IMPHY014988 | Limonene | 136.13 | 4.368 | 0 | 0 | 0 | 1 |
| IMPHY015094 | (+)-trans-Limonene oxide | 152.12 | 3.143 | 1 | 0 | 12.53 | 1 |
| IMPHY015095 | 2-Cyclohexen-1-ol, 1-methyl-4-(1-methylethyl)-, trans- | 154.14 | 2.739 | 1 | 1 | 20.23 | 1 |
| IMPHY015098 | trans-Verbenol | 152.12 | 2.998 | 1 | 1 | 20.23 | 0 |
| IMPHY015123 | alpha-Copaene | 204.19 | 5.437 | 0 | 0 | 0 | 1 |
| IMPHY015127 | alpha-Fenchyl alcohol | 154.14 | 2.765 | 1 | 1 | 20.23 | 0 |
| IMPHY015436 | 3-Buten-2-one, 4-(1,2,6,6-tetramethyl-2-cyclohexen-1-yl)- | 206.17 | 3.565 | 1 | 0 | 17.07 | 2 |
| IMPHY015562 | (1R,4S,5R)-4-methoxy-4-methyl-1-propan-2-ylbicyclo[3.1.0]hexane | 168.15 | 3.121 | 1 | 0 | 9.23 | 2 |
| IMPHY016027 | trans-Sabinene hydrate | 154.14 | 2.368 | 1 | 1 | 20.23 | 1 |
| IMPHY016037 | trans-4-Methoxythujane | 168.15 | 3.14 | 1 | 0 | 9.23 | 2 |
| IMPHY016046 | trans-4-Thujanol | 154.14 | 2.365 | 1 | 1 | 20.23 | 1 |
| IMPHY016054 | trans-alpha-Bergamotene | 204.19 | 5.755 | 0 | 0 | 0 | 3 |
| IMPHY017035 | gamma-Thujaplicin | 164.08 | 1.954 | 2 | 1 | 37.3 | 1 |
| IMPHY017038 | Alloisolongifolene | 204.19 | 4.126 | 0 | 0 | 0 | 1 |
| IMPHY017280 | 3-Methylcatechol | 124.05 | 1.359 | 2 | 2 | 40.46 | 0 |
| IMPHY017663 | alpha-Santalyl acetate | 262.19 | 4.45 | 2 | 0 | 26.3 | 6 |
| IMPHY017689 | 2'-Hydroxy-5'-methoxyacetophenone | 166.06 | 2.165 | 3 | 1 | 46.53 | 2 |
