# Supplementary material for: Exploring potential phytocompounds from black cumin as drug molecules against SARS-CoV-2 infections through bioinformatics analysis
Source: PLoS One. 2026 Mar 11;21(3):e0337970. doi: 10.1371/journal.pone.0337970 (PMC12978503; doi:10.1371/journal.pone.0337970)
Supplement: S6 Table — (DOCX) [file pone.0337970.s008.docx]

**S6 Table:** Ordered ligands/phytocompounds of black cumin based on the average of binding affinity scores (aBAS) across top-ranked 11 receptors, where the proposed top-ranked 3 phytocompounds with their BAS and aBAS were highlighted in green color, and the previously recommended major bioactive phytocompounds with their BAS and aBAS were highlighted in blue color.

| **Compounds ID** | **Phytocompounds Name** | **S** | **MAPK8** | **ACE2** | **RdRp** | **Plpro** | **TMPRSS2** | **Mpro** | **IL6** | **N** | **TNF** | **NFKBIA** | **Avg. Score** |
| --- | --- | --- | --- | --- | --- | --- | --- | --- | --- | --- | --- | --- | --- |
| IMPHY011677 | Taraxerol | -8.9 | -8.9 | -9.1 | -9.6 | -7.9 | -8.9 | -8.1 | -7.9 | -7.8 | -7.5 | -7.1 | -8.3 |
| IMPHY011703 | Silibinin | -8.9 | -9.4 | -9.6 | -8.2 | -7.5 | -8.8 | -7.7 | -7.2 | -8.3 | -8.2 | -7.4 | -8.3 |
| IMPHY012223 | beta-Amyrin | -8.9 | -9 | -9.5 | -9.1 | -7.8 | -8 | -8 | -7.8 | -7.8 | -7.5 | -7.7 | -8.3 |
| IMPHY006310 | Folic | -8.2 | -8.5 | -8.6 | -8.6 | -8.1 | -8 | -7.8 | -7.4 | -8.1 | -7.7 | -7.1 | -8 |
| IMPHY012769 | 24-Methylenelophenol | -8.6 | -7.7 | -9 | -8.2 | -7.5 | -7.9 | -7.8 | -7.7 | -7.3 | -7.2 | -7.3 | -7.8 |
| IMPHY011545 | Isoavenasterol | -8.5 | -7.9 | -9.2 | -8.5 | -7.1 | -6.9 | -7.8 | -7.6 | -7.5 | -7.5 | -7.1 | -7.8 |
| IMPHY011654 | alpha1-Sitosterol | -8.2 | -7.4 | -9.4 | -8 | -7.4 | -7.5 | -8 | -6.6 | -7.5 | -7.7 | -7.4 | -7.7 |
| IMPHY012003 | Betulinic acid | -8.5 | -8.5 | -8.5 | -8.2 | -7.3 | -7.8 | -8 | -6.7 | -7.3 | -7.3 | -6.3 | -7.7 |
| IMPHY011576 | alpha-Spinasterol | -7.9 | -7.7 | -9.3 | -8.3 | -7.2 | -7.3 | -7.6 | -7.4 | -7.3 | -7.3 | -6.8 | -7.6 |
| IMPHY004789 | 5-Dehydro-avenasterol | -8.2 | -7.6 | -7.6 | -8.6 | -7.4 | -7.3 | -7.8 | -7.3 | -7.4 | -7.7 | -7.1 | -7.6 |
| IMPHY002139 | Butyrospermol | -9.3 | -7.7 | -8.9 | -8 | -7.6 | -7.3 | -7.4 | -6.4 | -7.1 | -7.1 | -6.8 | -7.6 |
| IMPHY011642 | Cycloartenol | -8.7 | -8 | -8.9 | -8.7 | -6.9 | -7.3 | -7.5 | -7.2 | -6.6 | -6.5 | -7 | -7.6 |
| IMPHY007224 | Hederagenin | -7.6 | -7.5 | -8.6 | -8.6 | -7.4 | -7.4 | -7.9 | -7 | -7.6 | -7 | -6.6 | -7.6 |
| IMPHY014842 | Stigmasterol | -8.5 | -7.6 | -8.8 | -8.4 | -6.8 | -7.1 | -7.8 | -6.7 | -7.4 | -7.3 | -6.1 | -7.5 |
| IMPHY008729 | Lophenol | -7.6 | -7.6 | -8.7 | -7.7 | -7.2 | -7.1 | -7.7 | -7.4 | -7.2 | -6.3 | -7.2 | -7.4 |
| IMPHY011855 | Tirucallol | -8.4 | -7.7 | -8.4 | -7.8 | -7.1 | -7.8 | -7.3 | -6.8 | -6.5 | -6.9 | -6.9 | -7.4 |
| IMPHY011393 | 24-Methylene-cycloartanol | -8.6 | -7.4 | -8.5 | -8.4 | -6.7 | -7.2 | -7 | -6.5 | -6.7 | -7.3 | -6.7 | -7.4 |
| IMPHY012402 | Campesterol | -8.2 | -7.8 | -8.5 | -7.9 | -6.7 | -6.9 | -7.8 | -6.7 | -7 | -6.3 | -6.9 | -7.3 |
| IMPHY006659 | Obtusifoliol | -8.4 | -7.6 | -8.7 | -7.6 | -7 | -6.9 | -7.2 | -6.3 | -6.6 | -6.9 | -6.6 | -7.3 |
| IMPHY014899 | Stigmastanol | -7.7 | -7.7 | -8.6 | -7.6 | -6.8 | -7.1 | -7.3 | -6.6 | -7.2 | -6.5 | -6.4 | -7.2 |
| IMPHY002229 | Cycloeucalenol | -8.2 | -7.7 | -8.6 | -7.7 | -7.1 | -7 | -6.7 | -6.5 | -6.7 | -6.3 | -6.9 | -7.2 |
| IMPHY000437 | Nigellidine | -7.2 | -8.4 | -7.8 | -7.1 | -6.8 | -7.5 | -7.3 | -7 | -6.8 | -7 | -6.1 | -7.2 |
| IMPHY006300 | Cholesterol | -8.4 | -7.8 | -7.6 | -7.8 | -6.8 | -6.8 | -7.8 | -6.7 | -6.4 | -6.2 | -6.6 | -7.2 |
| IMPHY014836 | beta-Sitosterol | -7.6 | -7.1 | -8.2 | -7.8 | -7 | -7 | -7.5 | -6.6 | -6.5 | -6.6 | -6.3 | -7.1 |
| IMPHY000846 | Riboflavin | -7.5 | -7.3 | -7.9 | -8.1 | -6.2 | -7.8 | -7.1 | -6.7 | -6.4 | -6.4 | -5.9 | -7 |
| IMPHY008933 | 24-Ethyllophenol | -8.1 | -6.8 | -7.4 | -7.6 | -7 | -7.5 | -7.1 | -6.4 | -6.3 | -6.6 | -6.4 | -7 |
| IMPHY003467 | Dithymoquinone | -7.1 | -8 | -7.2 | -6.7 | -6.8 | -6.9 | -6.8 | -6.5 | -6.3 | -6.3 | -6.3 | -6.8 |
| IMPHY003459 | Pimara-8(14),15-diene | -7 | -6.8 | -6.9 | -7.5 | -6.8 | -6.7 | -6.9 | -6.3 | -6 | -6.2 | -6.2 | -6.7 |
| IMPHY001800 | Nigellicine | -6.7 | -8.6 | -6.4 | -6.6 | -5.8 | -7.5 | -6.3 | -6.4 | -6.1 | -6 | -5.4 | -6.5 |
| IMPHY013080 | alpha-Calacorene | -6.9 | -7.3 | -6.4 | -7.4 | -6.8 | -6.1 | -6.4 | -5.9 | -6.2 | -5.5 | -5.5 | -6.4 |
| IMPHY011709 | alpha-Eudesmol | -6.7 | -7.9 | -6.9 | -6.8 | -6.2 | -6.5 | -6.1 | -6.3 | -5.6 | -5.5 | -5.5 | -6.4 |
| IMPHY014817 | Aromadendrene | -6.6 | -8 | -6.4 | -6.9 | -6.4 | -6.2 | -6.4 | -6 | -5.5 | -5.4 | -5.4 | -6.3 |
| IMPHY005821 | gamma-Himachalene | -6.5 | -6.9 | -6.6 | -7.5 | -6.4 | -5.9 | -6 | -6.1 | -5.7 | -5.6 | -5.4 | -6.2 |
| IMPHY011542 | beta-Eudesmol | -6.7 | -7.5 | -6.5 | -6.9 | -6.2 | -6.9 | -6 | -5.6 | -5.5 | -5.6 | -5.2 | -6.2 |
| IMPHY011957 | (+)-delta-Cadinene | -6.4 | -7.1 | -6.5 | -6.9 | -6.4 | -6.2 | -5.9 | -5.9 | -6 | -5.6 | -5.5 | -6.2 |
| IMPHY011589 | 7-epi-alpha-Eudesmol | -6.4 | -6.4 | -6.7 | -6.7 | -6.2 | -6.4 | -6.6 | -6.1 | -5.7 | -5.6 | -5.4 | -6.2 |
| IMPHY011581 | alpha-Selinene | -6.1 | -7.6 | -6.6 | -6.8 | -6.3 | -6.3 | -6 | -5.9 | -5.4 | -5.7 | -5.5 | -6.2 |
| IMPHY007840 | Spathulenol | -6.7 | -7 | -6.4 | -6.8 | -6.2 | -6.4 | -6.3 | -6.3 | -5.4 | -5.4 | -5.1 | -6.2 |
| IMPHY014881 | Copaene | -7 | -6.3 | -6.9 | -6.5 | -6.6 | -6.1 | -6 | -5.8 | -5.7 | -5.4 | -5.5 | -6.2 |
| IMPHY012638 | Epizonarene | -6.6 | -7.5 | -6.8 | -6.9 | -5.9 | -6.1 | -5.8 | -5.8 | -5.6 | -5.6 | -5.2 | -6.2 |
| IMPHY014806 | Caswell No. 264AB | -6.7 | -6.4 | -6.5 | -6.4 | -6.5 | -6.3 | -6.3 | -5.9 | -5.8 | -5.6 | -5.3 | -6.2 |
| IMPHY015123 | alpha-Copaene | -7.2 | -6.3 | -7 | -6.4 | -6.6 | -6.2 | -5.7 | -6.1 | -5.2 | -5.3 | -5.6 | -6.1 |
| IMPHY014708 | beta-Selinene | -6.6 | -6.3 | -6.3 | -6.9 | -6.3 | -6.8 | -6.1 | -5.6 | -5.4 | -5.4 | -5.4 | -6.1 |
| IMPHY008451 | Isolongifolene | -6.4 | -6.6 | -5.8 | -7.5 | -6.1 | -6.1 | -6.5 | -6.2 | -5.4 | -5.2 | -5.2 | -6.1 |
| IMPHY011793 | (+)-gamma-Cadinene | -6.8 | -7.1 | -6.8 | -6.5 | -6 | -6.1 | -5.7 | -5.6 | -5.8 | -5.6 | -5 | -6.1 |
| IMPHY010815 | Thujopsene | -6.2 | -6.5 | -6.5 | -6.9 | -6.2 | -6.1 | -6.1 | -6.3 | -5.5 | -5.3 | -5.3 | -6.1 |
| IMPHY010609 | (-)-alpha-Himachalene | -6.7 | -6.2 | -6.2 | -6.7 | -6.2 | -6.4 | -6.2 | -6 | -5.3 | -5.3 | -5.7 | -6.1 |
| IMPHY014513 | Longiborneol acetate | -6.3 | -6.8 | -6.2 | -6.8 | -6.5 | -6.2 | -6 | -6.1 | -5.4 | -5.2 | -5.3 | -6.1 |
| IMPHY012738 | Isocaryophyllene | -6.3 | -7.1 | -6.7 | -6.3 | -6.3 | -6.1 | -6.1 | -5.9 | -5.7 | -5.1 | -5.2 | -6.1 |
| IMPHY003695 | (-)-Germacrene A | -6.7 | -6.5 | -6.4 | -6.6 | -6.1 | -5.9 | -6.2 | -6 | -5.7 | -5.4 | -5.2 | -6.1 |
| IMPHY009840 | Cyclosativene | -6 | -6.7 | -7 | -6.7 | -6.3 | -6 | -6 | -6.2 | -5.3 | -5 | -5.4 | -6.1 |
| IMPHY004286 | Longifolene | -5.9 | -6.3 | -6 | -7.1 | -6.7 | -6 | -6.4 | -6.1 | -5.3 | -5.3 | -5.2 | -6 |
| IMPHY009737 | beta-Longipinene | -6.7 | -6.6 | -6 | -6.5 | -6.4 | -5.7 | -6.3 | -5.8 | -5.3 | -5.5 | -5.5 | -6 |
| IMPHY012737 | (1S,4E,9S)-4,11,11-trimethyl-8-methylidenebicyclo[7.2.0]undec-4-ene | -6.1 | -6.5 | -6.2 | -6.5 | -6.5 | -6.2 | -6 | -6.1 | -5.8 | -5.1 | -5.3 | -6 |
| IMPHY003977 | (-)-beta-Bourbonene | -6.4 | -6.4 | -6.8 | -6.4 | -6.1 | -6.2 | -6.2 | -5.7 | -5.5 | -5.3 | -5.2 | -6 |
| IMPHY011894 | (-)-7-Epi-alpha-selinene | -6.7 | -6.7 | -6.2 | -6.4 | -6.3 | -6.9 | -5.6 | -5.6 | -5.7 | -5.1 | -5 | -6 |
| IMPHY005974 | Longicyclene | -6.4 | -6.4 | -5.9 | -6.5 | -6.4 | -5.9 | -6.3 | -6.3 | -5.6 | -5.5 | -5 | -6 |
| IMPHY012279 | alpha-Curcumene | -7.3 | -7.4 | -6.3 | -6.4 | -6 | -5.4 | -5.2 | -5.3 | -5.5 | -5.5 | -5.8 | -6 |
| IMPHY011839 | (Z)-gamma-bisabolene | -7.4 | -7.5 | -6.9 | -5.5 | -6.4 | -5.4 | -5.3 | -5.8 | -4.8 | -5.2 | -5.8 | -6 |
| IMPHY004216 | (1S,2S,7S,8S)-2,6,6,9-tetramethyltricyclo[5.4.0.02,8]undec-9-ene | -6.1 | -6.3 | -5.9 | -6.8 | -6.2 | -6 | -6.4 | -6.2 | -5.4 | -5.5 | -5.1 | -6 |
| IMPHY014907 | 6-Epi-beta-bisabolol | -6.5 | -7 | -6.5 | -5.7 | -6.8 | -6 | -5.9 | -5.7 | -5.3 | -5 | -5.3 | -6 |
| IMPHY011761 | Humulene | -6.2 | -6.4 | -6.7 | -6 | -6.1 | -5.8 | -6 | -6.2 | -5.7 | -5.3 | -5.2 | -6 |
| IMPHY000399 | beta-Bisabolene | -7.6 | -7.2 | -6.6 | -5.7 | -5.8 | -5.8 | -5.4 | -5.1 | -5.4 | -4.9 | -6 | -6 |
| IMPHY014831 | beta-Caryophyllene | -6.2 | -6.5 | -6.3 | -6.4 | -6 | -5.9 | -6 | -5.8 | -5.7 | -5.3 | -5.2 | -5.9 |
| IMPHY012665 | Levomenol | -6.9 | -7.2 | -6.5 | -6.1 | -5.6 | -5.8 | -5.5 | -5.5 | -5.1 | -5.2 | -5.8 | -5.9 |
| IMPHY004215 | (1R,2R,7R,8R)-2,6,6,9-tetramethyltricyclo[5.4.0.02,8]undec-9-ene | -5.6 | -6.3 | -5.9 | -6.8 | -6.2 | -6.1 | -6.4 | -5.8 | -5.4 | -5.5 | -5.2 | -5.9 |
| IMPHY017663 | alpha-Santalyl acetate | -6 | -6.6 | -6.5 | -6.9 | -6.2 | -6 | -5.6 | -5.7 | -5.4 | -5.2 | -4.7 | -5.9 |
| IMPHY016054 | trans-alpha-Bergamotene | -6.8 | -6.6 | -6.5 | -6.3 | -5.9 | -5.3 | -5.9 | -5.8 | -4.7 | -5.3 | -5.2 | -5.8 |
| IMPHY010712 | Nigellimine n-oxide | -6.5 | -7.1 | -5.8 | -5.7 | -5.7 | -7.2 | -5.6 | -5 | -5.3 | -5.3 | -4.8 | -5.8 |
| IMPHY017038 | Alloisolongifolene | -5.8 | -6.3 | -5.7 | -6.3 | -6.3 | -6.5 | -6 | -5.7 | -5.4 | -4.9 | -4.8 | -5.8 |
| IMPHY003490 | Coumarin | -6.8 | -6.7 | -7.1 | -5.6 | -5.7 | -6.1 | -5.7 | -5.3 | -4.9 | -5 | -4.6 | -5.8 |
| IMPHY014249 | Naphthalen-1(2h)-one | -6.8 | -6.8 | -5.8 | -5.8 | -5.7 | -6.1 | -5.7 | -5.3 | -5 | -5.1 | -4.7 | -5.7 |
| IMPHY014866 | 2-Cyclohexen-1-ol, 2-methyl-5-(1-methylethenyl)-, acetate, cis- | -6.9 | -7.1 | -5.8 | -5.6 | -5.4 | -5.9 | -5.2 | -5.7 | -5 | -5.3 | -4.7 | -5.7 |
| IMPHY014219 | 1,2-Dihydronaphthalen-2-one | -6 | -7 | -5.7 | -5.9 | -6.5 | -5.8 | -5.6 | -5.2 | -5.1 | -5.1 | -4.6 | -5.7 |
| IMPHY012921 | gamma-Elemene | -5.8 | -6.1 | -5.7 | -5.8 | -6.2 | -5.5 | -5.8 | -5.5 | -5.8 | -5.3 | -5 | -5.7 |
| IMPHY017035 | gamma-Thujaplicin | -6.1 | -6.6 | -5.9 | -5.6 | -5.8 | -6.6 | -5.5 | -5.5 | -5 | -4.8 | -4.8 | -5.7 |
| IMPHY010080 | beta-Elemene | -5.8 | -6.9 | -5.6 | -6.4 | -5.9 | -5.9 | -5.4 | -5.3 | -5.2 | -5 | -4.8 | -5.7 |
| IMPHY004209 | Davanone D | -6.5 | -5.9 | -5.8 | -6 | -5.9 | -5.8 | -5.2 | -5.4 | -5.4 | -5.1 | -5 | -5.6 |
| IMPHY008483 | 6,7-Dimethoxy-1-methylisoquinoline | -6.6 | -7.1 | -5.8 | -5.7 | -5.1 | -5.7 | -5.4 | -5.4 | -4.9 | -5.5 | -4.7 | -5.6 |
| IMPHY009865 | alpha,3-Dimethylstyrene | -6.8 | -6.3 | -6.3 | -5.4 | -6.4 | -5.5 | -5.1 | -5.2 | -5.2 | -4.8 | -4.6 | -5.6 |
| IMPHY011777 | Farnesyl acetate | -6.4 | -6 | -6.5 | -6.1 | -5.3 | -5.6 | -5.4 | -5.1 | -4.8 | -5.5 | -4.9 | -5.6 |
| IMPHY011745 | Zingiberene | -7 | -7.3 | -6.6 | -5.3 | -5.6 | -5 | -5.2 | -4.8 | -4.5 | -4.2 | -5.7 | -5.6 |
| IMPHY001246 | Carvacrol | -6.9 | -6.3 | -5.7 | -5.4 | -5.9 | -5.6 | -5.2 | -5.4 | -4.9 | -5.3 | -4.5 | -5.6 |
| IMPHY006550 | Thymol | -7.2 | -6.1 | -5.8 | -5.4 | -5.6 | -5.7 | -5.2 | -5.1 | -5 | -5.1 | -4.6 | -5.5 |
| IMPHY011632 | Farnesol | -6.2 | -6.8 | -6.4 | -5.7 | -5.1 | -5.7 | -5.5 | -4.5 | -5 | -5.1 | -4.8 | -5.5 |
| IMPHY000602 | M-Cymene | -6.8 | -6.4 | -6.3 | -5.6 | -5.6 | -5.3 | -5.2 | -5 | -5.1 | -4.8 | -4.6 | -5.5 |
| IMPHY005569 | alpha-Ionone | -5.6 | -5.7 | -5.7 | -5.8 | -6.5 | -5.3 | -5.5 | -5.9 | -5.1 | -4.8 | -4.7 | -5.5 |
| IMPHY007606 | Thymohydroquinone | -6.7 | -6.2 | -5.5 | -5.2 | -5.6 | -6.2 | -5.4 | -5.4 | -4.9 | -5.1 | -4.4 | -5.5 |
| IMPHY015436 | 3-Buten-2-one, 4-(1,2,6,6-tetramethyl-2-cyclohexen-1-yl)- | -6.3 | -5.7 | -5.5 | -6 | -5.8 | -5.3 | -5.3 | -5.6 | -5.1 | -5.2 | -4.8 | -5.5 |
| IMPHY000005 | Thiamine | -6.2 | -5.6 | -5.5 | -5.6 | -5.9 | -5.6 | -5.5 | -5.2 | -5.5 | -5.3 | -4.5 | -5.5 |
| IMPHY011557 | 4-Isopropylbenzyl alcohol | -6.5 | -6.3 | -6.7 | -5.3 | -5.6 | -5.4 | -5.4 | -5 | -4.7 | -4.9 | -4.6 | -5.5 |
| IMPHY011371 | cis-Chrysanthenyl acetate | -5.8 | -5.7 | -5.4 | -5.8 | -5.6 | -5.7 | -5.3 | -6.2 | -5.2 | -4.9 | -4.6 | -5.5 |
| IMPHY013782 | 1-Ethyl-2,3-dimethylbenzene | -6.8 | -6.1 | -6.8 | -5.5 | -5.6 | -5.5 | -5.2 | -4.7 | -5 | -4.8 | -4.2 | -5.5 |
| IMPHY006709 | Acetyleugenol | -6.7 | -6.6 | -5.5 | -5.4 | -5.6 | -5.7 | -5.1 | -5 | -4.7 | -4.7 | -4.9 | -5.4 |
| IMPHY003536 | Eugenol | -6.4 | -5.9 | -5.9 | -5.1 | -5.4 | -6.2 | -5.5 | -5.2 | -4.7 | -5.1 | -4.4 | -5.4 |
| IMPHY003982 | gamma-Terpinene | -6.7 | -6.1 | -7 | -5.4 | -5.7 | -5.2 | -5 | -4.7 | -4.8 | -4.7 | -4.5 | -5.4 |
| IMPHY002825 | 2-(4-Methylphenyl)propan-2-ol | -6.8 | -6.2 | -5.9 | -5.1 | -5.6 | -5.5 | -5.3 | -5.1 | -4.9 | -4.7 | -4.6 | -5.4 |
| IMPHY003545 | 4-Isopropylbenzaldehyde | -6.8 | -6.3 | -6.3 | -5.3 | -5.6 | -5.2 | -5.3 | -4.7 | -4.8 | -4.9 | -4.4 | -5.4 |
| IMPHY008150 | 1-Methyl-4-(prop-1-en-2-yl)benzene | -6.7 | -6.2 | -6.8 | -5.3 | -5.7 | -5.3 | -5 | -4.7 | -4.8 | -4.7 | -4.4 | -5.4 |
| IMPHY011631 | (E,Z)-farnesol | -6 | -6.5 | -6.4 | -5.6 | -6.2 | -5.4 | -4.6 | -4.7 | -4.6 | -5.2 | -4.3 | -5.4 |
| IMPHY009866 | 2-(4-Methylphenyl)propan-1-ol | -6.6 | -6.1 | -5.9 | -5.2 | -5.6 | -5.4 | -5.6 | -5.2 | -4.9 | -4.7 | -4.3 | -5.4 |
| IMPHY014847 | Bornyl acetate | -5.9 | -5.4 | -5.8 | -5.6 | -5.5 | -5.6 | -5.3 | -6.2 | -4.8 | -4.7 | -4.6 | -5.4 |
| IMPHY011973 | (-)-cis-Carveol | -6.7 | -6.2 | -5.3 | -5.3 | -5.8 | -5.7 | -5.2 | -5.2 | -4.8 | -4.8 | -4.4 | -5.4 |
| IMPHY001658 | Thymol methyl ether | -7 | -6.2 | -5.9 | -4.9 | -5.8 | -5.4 | -5.1 | -5.1 | -4.8 | -4.7 | -4.4 | -5.4 |
| IMPHY006145 | p-Cymene | -6.8 | -6.2 | -6 | -5.4 | -5.8 | -5.2 | -5.1 | -4.8 | -4.8 | -4.7 | -4.5 | -5.4 |
| IMPHY011599 | Terpinolene | -7.2 | -6.2 | -5.7 | -5.4 | -6.1 | -5.4 | -4.9 | -4.7 | -4.8 | -4.6 | -4.3 | -5.4 |
| IMPHY011570 | (2Z,6E)-Farnesyl acetate | -6 | -5 | -6.1 | -5.6 | -5.8 | -5.3 | -4.5 | -5.5 | -5.1 | -5.4 | -4.9 | -5.4 |
| IMPHY012144 | 1-Methyl-3-propylbenzene | -6.5 | -6.1 | -6.6 | -5.4 | -5.5 | -5.1 | -4.8 | -4.9 | -5 | -4.8 | -4.5 | -5.4 |
| IMPHY011884 | Pulegone | -5.9 | -6.7 | -5.4 | -5.4 | -5.5 | -5.5 | -5.2 | -5 | -4.9 | -5 | -4.6 | -5.4 |
| IMPHY001218 | Thymoquinone | -5.5 | -6.4 | -5.5 | -5.4 | -5.6 | -5.6 | -5.2 | -5.5 | -4.9 | -4.9 | -4.4 | -5.4 |
| IMPHY017689 | 2'-Hydroxy-5'-methoxyacetophenone | -6.3 | -5.8 | -5.3 | -5.3 | -5.3 | -5.9 | -5.1 | -5.4 | -4.9 | -5.1 | -4.5 | -5.4 |
| IMPHY000545 | O-Cymene | -6.8 | -6 | -5.4 | -5.3 | -6.1 | -5.4 | -5.2 | -4.8 | -5 | -4.5 | -4.3 | -5.3 |
| IMPHY011988 | (-)-trans-Carveol | -6.5 | -6.1 | -5.7 | -5.1 | -5.6 | -5.8 | -4.8 | -5.4 | -4.8 | -4.5 | -4.5 | -5.3 |
| IMPHY003398 | Myristicin | -6.4 | -6.5 | -5.4 | -5.1 | -5.4 | -5.4 | -5.1 | -5.1 | -4.7 | -5 | -4.6 | -5.3 |
| IMPHY011354 | trans-Sabinene hydrate acetate | -5.9 | -5.6 | -5.3 | -5.4 | -5.6 | -6 | -5.4 | -5.2 | -4.7 | -4.7 | -4.9 | -5.3 |
| IMPHY012075 | Carvone | -6.6 | -6.4 | -5.5 | -5.2 | -5.9 | -5.2 | -4.8 | -5.4 | -4.6 | -4.6 | -4.5 | -5.3 |
| IMPHY011643 | alpha-Terpinene | -6.8 | -6.2 | -5.4 | -5.5 | -6.1 | -5.2 | -4.9 | -4.8 | -4.9 | -4.5 | -4.4 | -5.3 |
| IMPHY011004 | p-Mentha-1,3,8-triene | -6.9 | -6.2 | -6.3 | -5.3 | -5.6 | -5.3 | -4.8 | -4.6 | -4.6 | -4.5 | -4.5 | -5.3 |
| IMPHY012160 | alpha-Terpineol | -6.4 | -6 | -5.9 | -5.2 | -5.6 | -5.5 | -5.2 | -5 | -4.9 | -4.5 | -4.4 | -5.3 |
| IMPHY012255 | (+)-trans-Piperitenol | -6.6 | -6.1 | -5.6 | -5.1 | -5.5 | -5.5 | -5 | -5.1 | -4.9 | -4.7 | -4.5 | -5.3 |
| IMPHY017280 | 3-Methylcatechol | -6.2 | -5.4 | -6 | -5 | -5.3 | -6.4 | -4.9 | -5 | -4.8 | -5.2 | -4.3 | -5.3 |
| IMPHY006362 | Ascorbic acid | -5.3 | -5.2 | -5.7 | -5.3 | -5.5 | -6.3 | -5.1 | -5.3 | -5.5 | -5 | -4.3 | -5.3 |
| IMPHY011658 | beta-Farnesene | -6.3 | -6.6 | -6.1 | -5.2 | -5.2 | -5.5 | -4.9 | -4.2 | -4.9 | -4.7 | -4.7 | -5.3 |
| IMPHY014872 | cis-Pinocarveol | -5.7 | -5.5 | -5.6 | -5.6 | -5.4 | -5.6 | -5.2 | -5.7 | -4.7 | -4.6 | -4.6 | -5.3 |
| IMPHY011558 | Apiole | -5.6 | -6.5 | -5.5 | -5.2 | -5.3 | -5.8 | -5.4 | -5 | -4.7 | -4.8 | -4.4 | -5.3 |
| IMPHY014811 | alpha-Phellandrene | -6.6 | -6.2 | -5.6 | -5.4 | -5.5 | -5.2 | -4.8 | -4.9 | -4.8 | -4.7 | -4.5 | -5.3 |
| IMPHY009871 | Isoterpinolene | -6.2 | -6.3 | -5.8 | -5.4 | -5.4 | -5.2 | -5 | -4.8 | -4.9 | -4.6 | -4.5 | -5.3 |
| IMPHY015098 | trans-Verbenol | -5.7 | -5.3 | -5.3 | -5.7 | -5.7 | -5.7 | -5.4 | -5.6 | -4.6 | -4.6 | -4.4 | -5.3 |
| IMPHY014988 | Limonene | -6.7 | -6.1 | -5.7 | -5.2 | -5.6 | -5.2 | -4.9 | -4.9 | -4.7 | -4.4 | -4.5 | -5.3 |
| IMPHY015095 | 2-Cyclohexen-1-ol, 1-methyl-4-(1-methylethyl)-, trans- | -6.3 | -6 | -5.7 | -5.3 | -5 | -5.7 | -5.1 | -5.3 | -4.5 | -4.6 | -4.4 | -5.3 |
| IMPHY011648 | Neryl acetate | -6.2 | -6.1 | -5.5 | -5.3 | -5.5 | -5.5 | -4.9 | -4.7 | -5 | -4.6 | -4.5 | -5.3 |
| IMPHY001548 | Geranylacetone | -5.9 | -6.3 | -5.9 | -5.1 | -4.8 | -5.3 | -5 | -4.9 | -4.7 | -4.9 | -4.9 | -5.2 |
| IMPHY013764 | (-)-Carvomenthone | -6.2 | -6.3 | -5.6 | -5.3 | -5.4 | -5.4 | -4.8 | -5.2 | -4.7 | -4.4 | -4.4 | -5.2 |
| IMPHY001817 | p-Menth-3-en-1-ol | -5.6 | -5.9 | -5.4 | -5.2 | -5.6 | -5.8 | -4.9 | -5.1 | -4.9 | -4.7 | -4.6 | -5.2 |
| IMPHY011882 | Cinnamaldehyde | -6.2 | -6 | -6.1 | -5.1 | -5.4 | -5.1 | -5.1 | -4.7 | -4.8 | -4.8 | -4.4 | -5.2 |
| IMPHY014873 | 2-Cyclohexen-1-ol, 3-methyl-6-(1-methylethyl)-, (1R,6S)-rel- | -6.2 | -6.1 | -5.7 | -5.2 | -5.5 | -5.3 | -4.8 | -5 | -4.7 | -4.6 | -4.5 | -5.2 |
| IMPHY011552 | (1R)-2-methyl-5-propan-2-ylbicyclo[3.1.0]hex-2-ene | -6.2 | -6 | -6.1 | -5.3 | -5.5 | -5.3 | -4.8 | -4.8 | -4.8 | -4.4 | -4.4 | -5.2 |
| IMPHY012002 | (+)-Dihydrocarvone | -6.1 | -6.3 | -5.5 | -5.3 | -5.6 | -5.2 | -4.9 | -5.3 | -4.6 | -4.5 | -4.3 | -5.2 |
| IMPHY012175 | D-Limonene | -6.7 | -6.1 | -5.6 | -5.3 | -5.5 | -5.2 | -4.8 | -4.8 | -4.6 | -4.6 | -4.4 | -5.2 |
| IMPHY013977 | p-Mentha-1,5,8-triene | -6.2 | -5.7 | -5.9 | -5.4 | -5.7 | -5.2 | -4.9 | -4.8 | -5.1 | -4.3 | -4.4 | -5.2 |
| IMPHY012205 | Sabinene hydrate | -5.7 | -5.6 | -5.8 | -5.1 | -5.4 | -5.7 | -4.9 | -5.5 | -5 | -4.5 | -4.3 | -5.2 |
| IMPHY011396 | 4-Carvomenthenol | -5.3 | -5.6 | -5.3 | -5.3 | -5.7 | -5.4 | -5 | -5.3 | -4.8 | -5.1 | -4.3 | -5.2 |
| IMPHY006243 | 2,5-Dimethoxy-p-cymene | -5.7 | -6.6 | -5.2 | -5.1 | -5.1 | -5.2 | -5.1 | -5.2 | -4.8 | -4.7 | -4.3 | -5.2 |
| IMPHY011763 | Anethole | -6.5 | -6.2 | -5.5 | -5.4 | -5.3 | -5.3 | -4.6 | -4.7 | -4.6 | -4.5 | -4.4 | -5.2 |
| IMPHY013836 | Fenchone | -5.7 | -5.3 | -6 | -5.7 | -5.2 | -5.6 | -5.1 | -5.4 | -4.3 | -4.4 | -4.3 | -5.2 |
| IMPHY001144 | Dillapiol | -5.8 | -6.2 | -5.6 | -5 | -5 | -5.4 | -5.2 | -4.9 | -4.6 | -4.7 | -4.5 | -5.2 |
| IMPHY016027 | trans-Sabinene hydrate | -5.7 | -5.6 | -5.6 | -5.1 | -5.4 | -5.7 | -4.9 | -5 | -5 | -4.6 | -4.3 | -5.2 |
| IMPHY000158 | Pyridoxine | -5.7 | -5.4 | -5.1 | -4.9 | -5.4 | -5.5 | -5.2 | -5.4 | -5 | -5 | -4.2 | -5.2 |
| IMPHY009752 | beta-Cyclocitral | -5.6 | -6 | -5.3 | -5.3 | -5.1 | -5.4 | -5.4 | -4.9 | -4.8 | -4.6 | -4.3 | -5.2 |
| IMPHY002072 | Pinocarvone | -5.7 | -5.3 | -5.4 | -5.5 | -5.4 | -5.1 | -5.1 | -5.6 | -4.4 | -4.5 | -4.6 | -5.1 |
| IMPHY006163 | 2,2,5-Trimethyl-4-cyclohepten-1-one | -5.5 | -5.3 | -5.4 | -5.4 | -5.7 | -5.3 | -5.2 | -5.3 | -4.6 | -4.6 | -4.3 | -5.1 |
| IMPHY015562 | (1R,4S,5R)-4-methoxy-4-methyl-1-propan-2-ylbicyclo[3.1.0]hexane | -5.8 | -5.9 | -5.3 | -5.5 | -5.2 | -5.7 | -4.8 | -5.1 | -4.6 | -4.6 | -4.1 | -5.1 |
| IMPHY012165 | Sabinene | -6.5 | -5.6 | -5.6 | -5.2 | -5.5 | -5.3 | -4.6 | -4.8 | -4.5 | -4.4 | -4.4 | -5.1 |
| IMPHY016037 | trans-4-Methoxythujane | -5.8 | -5.3 | -5.5 | -5.4 | -5.2 | -5.7 | -5 | -5.1 | -4.5 | -4.5 | -4.2 | -5.1 |
| IMPHY006678 | 4-Acetyl-1,4-dimethyl-1-cyclohexene | -5.8 | -5.5 | -5.1 | -5.5 | -5.5 | -5.4 | -5.2 | -5 | -4.7 | -4.3 | -4.1 | -5.1 |
| IMPHY000108 | (3S,4S)-4-ethenyl-4-methyl-3-prop-1-en-2-ylcyclohexene | -5.3 | -5.9 | -5.4 | -5.4 | -5.4 | -5.2 | -5.1 | -5.2 | -4.6 | -4.4 | -4.1 | -5.1 |
| IMPHY015094 | (+)-trans-Limonene oxide | -5.4 | -5.9 | -5.5 | -4.9 | -5.4 | -5.6 | -4.7 | -5 | -4.6 | -4.6 | -4.4 | -5.1 |
| IMPHY015127 | alpha-Fenchyl alcohol | -5.5 | -5.3 | -5.3 | -5.3 | -5.5 | -5.4 | -5.1 | -5.3 | -4.5 | -4.5 | -4.3 | -5.1 |
| IMPHY010781 | Limonene oxide, cis-(-)- | -5.5 | -6.1 | -5.4 | -5 | -5.5 | -5.4 | -4.8 | -4.9 | -4.5 | -4.5 | -4.4 | -5.1 |
| IMPHY006696 | Methyleugenol | -6.5 | -6 | -5.2 | -5.1 | -5.1 | -5.3 | -4.7 | -4.6 | -4.5 | -4.6 | -4.3 | -5.1 |
| IMPHY000099 | Myrtenol | -5.2 | -5.3 | -5.4 | -5.2 | -5.3 | -5.3 | -5.2 | -5.4 | -4.6 | -4.6 | -4.3 | -5.1 |
| IMPHY006944 | Estragole | -6.3 | -5.9 | -5.6 | -5.1 | -5.2 | -5.2 | -4.6 | -4.6 | -4.5 | -4.5 | -4.2 | -5.1 |
| IMPHY014852 | Camphene | -5.4 | -5.3 | -5.6 | -5.8 | -5.3 | -5.1 | -5 | -5.3 | -4.4 | -4.4 | -4.1 | -5.1 |
| IMPHY014942 | Bicyclo[2.2.1]heptan-2-ol, 1,7,7-trimethyl-, formate, (1R,2R,4R)-rel- | -5.3 | -5.2 | -5 | -5.7 | -5.2 | -4.9 | -5.5 | -5.5 | -4.3 | -4.5 | -4.4 | -5 |
| IMPHY010072 | Eucalyptol | -5.4 | -5.1 | -5.7 | -5.5 | -5.1 | -5 | -5.1 | -5.1 | -5 | -4.2 | -4.2 | -5 |
| IMPHY006177 | Methyl geranate | -5.7 | -6 | -5.2 | -5.2 | -5 | -5.2 | -4.9 | -4.8 | -4.6 | -4.5 | -4.3 | -5 |
| IMPHY014207 | (2E,4E)-3,7-dimethylocta-2,4,6-trienal | -5.9 | -5.9 | -5.5 | -5 | -5 | -5.1 | -4.7 | -4.6 | -4.7 | -4.6 | -4.3 | -5 |
| IMPHY006325 | Myrtenal | -5.3 | -5 | -5.2 | -5.4 | -5.5 | -5.3 | -5.2 | -5.1 | -4.4 | -4.4 | -4.3 | -5 |
| IMPHY011590 | d-Borneol | -5.6 | -5 | -4.8 | -5.2 | -5.2 | -5 | -5.6 | -5.1 | -4.5 | -4.7 | -4.4 | -5 |
| IMPHY009764 | 2,10-Epoxypinane | -5.3 | -5 | -5.2 | -5.5 | -5.1 | -5.3 | -5 | -5.3 | -4.3 | -4.4 | -4.6 | -5 |
| IMPHY012061 | alpha-Pinene | -5.7 | -4.9 | -5.5 | -5.7 | -5.2 | -4.9 | -4.9 | -5.4 | -4.3 | -4.2 | -4.3 | -5 |
| IMPHY012036 | Camphor | -5.4 | -5.2 | -4.8 | -5.8 | -5.2 | -4.9 | -5.2 | -5.1 | -4.4 | -4.5 | -4.2 | -5 |
| IMPHY007286 | Umbellulon | -4.7 | -6.2 | -5.3 | -4.9 | -5.3 | -5.5 | -4.9 | -4.8 | -4.6 | -4.2 | -4.3 | -5 |
| IMPHY014874 | cis-Sabinene hydrate | -5.7 | -5.5 | -5.4 | -5.5 | -5.1 | -4.8 | -4.7 | -4.9 | -4.7 | -4.1 | -4.2 | -5 |
| IMPHY012147 | beta-Pinene | -5.5 | -4.9 | -5.7 | -5.6 | -5.3 | -4.8 | -5 | -5 | -4.2 | -4.3 | -4.3 | -5 |
| IMPHY016046 | trans-4-Thujanol | -4.8 | -5.5 | -5.3 | -5 | -5.6 | -5.1 | -5.1 | -4.8 | -4.9 | -4.3 | -4.2 | -5 |
| IMPHY011789 | Citral | -6.1 | -5.9 | -5.3 | -5.2 | -5.1 | -4.9 | -4.6 | -4.8 | -4.4 | -4.3 | -3.9 | -5 |
| IMPHY003567 | alpha-Fenchene | -5.4 | -5.3 | -5.1 | -5.8 | -5.4 | -5 | -4.8 | -4.9 | -4 | -4.3 | -4.3 | -4.9 |
| IMPHY006950 | Tricyclene | -5.3 | -4.9 | -5.3 | -5.8 | -5.2 | -5 | -5 | -5.1 | -4.1 | -4.2 | -4.2 | -4.9 |
| IMPHY014835 | (E)-beta-ocimene | -5.8 | -5.8 | -5.2 | -4.7 | -5.2 | -4.8 | -4.5 | -4.7 | -4.5 | -4.4 | -4.3 | -4.9 |
| IMPHY007067 | Linalyl acetate | -5.2 | -5.7 | -5 | -4.8 | -5.2 | -5 | -4.5 | -4.9 | -4.5 | -4.8 | -4.2 | -4.9 |
| IMPHY007357 | Nicotinic acid | -5.3 | -5 | -5.3 | -4.8 | -5.2 | -5.5 | -4.6 | -4.8 | -4.5 | -4.4 | -4 | -4.9 |
| IMPHY012739 | (Z)-beta-Ocimene | -5.8 | -5.8 | -5.3 | -5 | -5.1 | -4.6 | -4.4 | -4.3 | -4.2 | -4.5 | -4.2 | -4.8 |
| IMPHY009946 | Benzaldehyde | -5.5 | -5 | -5.8 | -4.9 | -5 | -4.9 | -4.5 | -4.8 | -4.3 | -4.1 | -3.8 | -4.8 |
| IMPHY002053 | D-arabinonic acid | -4.9 | -4.7 | -5.1 | -5 | -4.7 | -5.9 | -4.3 | -4.5 | -5 | -4.4 | -4 | -4.8 |
| IMPHY012058 | Linalool | -5.5 | -5.3 | -5.2 | -4.5 | -5 | -4.8 | -4.2 | -5 | -4.7 | -4.3 | -4 | -4.8 |
| IMPHY007421 | Citronellyl acetate | -5.3 | -5.6 | -5.2 | -4.9 | -5 | -4.9 | -4.5 | -4.3 | -4.2 | -4.4 | -4.1 | -4.8 |
| IMPHY003482 | 4-Methoxybenzaldehyde | -5.7 | -5.4 | -5.2 | -4.4 | -5 | -4.9 | -4.7 | -4.6 | -4.1 | -4.2 | -3.9 | -4.7 |
| IMPHY013838 | 3,7-Dimethyloct-6-en-3-ol | -5.4 | -5.2 | -5.1 | -4.5 | -4.5 | -4.9 | -4.8 | -4.7 | -4.5 | -4.3 | -4.1 | -4.7 |
| IMPHY003485 | Myrcene | -5.8 | -5.2 | -5.7 | -4.4 | -5.1 | -4.5 | -4.4 | -4.2 | -4.1 | -4.3 | -4.2 | -4.7 |
| IMPHY012654 | Nerol | -5.1 | -4.6 | -5.4 | -4.6 | -5 | -5.1 | -4.3 | -4.6 | -4.4 | -4.6 | -3.8 | -4.7 |
| IMPHY008162 | Citronellyl butyrate | -5 | -5.8 | -5 | -4.4 | -4.8 | -4.4 | -4.5 | -4.2 | -4.3 | -4.6 | -4.3 | -4.7 |
| IMPHY003723 | 2,4-Decadienal | -5.2 | -5.4 | -5.2 | -4.6 | -4.6 | -4.6 | -4.3 | -4.4 | -4.3 | -4.1 | -3.9 | -4.6 |
| IMPHY012082 | Gamma-nonalactone | -5.7 | -5.4 | -4.8 | -4.5 | -4.7 | -4.8 | -4.5 | -4.3 | -4.1 | -3.9 | -3.8 | -4.6 |
| IMPHY003104 | Decanoic acid | -4.8 | -5.3 | -5.2 | -4.6 | -5 | -4.6 | -4.3 | -4.1 | -4.6 | -4.1 | -3.8 | -4.6 |
| IMPHY001881 | 2-Tridecanone | -5 | -5.3 | -5.2 | -4.6 | -4.7 | -4.4 | -3.8 | -4.5 | -4.3 | -4 | -3.7 | -4.5 |
| IMPHY014749 | (E,Z)-2,4-Decadienal | -5 | -5.1 | -5 | -4.5 | -4.5 | -4.7 | -3.9 | -4.1 | -4.5 | -4.1 | -3.7 | -4.5 |
| IMPHY003016 | Lauric acid | -4.9 | -5.3 | -5.5 | -4.1 | -4.7 | -4.2 | -4.5 | -4 | -4.5 | -3.9 | -3.5 | -4.5 |
| IMPHY013804 | Octyl isobutyrate | -4.2 | -5 | -4.9 | -4.6 | -4.8 | -4.4 | -4.1 | -4.1 | -3.9 | -4.3 | -3.6 | -4.4 |
| IMPHY007204 | Dodecanal | -4.8 | -5.2 | -5.1 | -4.5 | -4 | -4.3 | -3.9 | -3.7 | -4 | -4 | -4 | -4.3 |
| IMPHY003915 | 2-Decenal | -5.4 | -4.8 | -5 | -4 | -4.5 | -4 | -3.8 | -4.1 | -4.2 | -3.8 | -3.8 | -4.3 |
| IMPHY006947 | 1-Decanol | -5 | -5 | -4.8 | -4.1 | -4.8 | -4.3 | -4 | -3.6 | -4.1 | -3.7 | -3.7 | -4.3 |
| IMPHY003525 | Nonanal | -4.3 | -4.8 | -4.7 | -4.1 | -4.6 | -4.3 | -3.9 | -4.1 | -3.9 | -3.6 | -3.6 | -4.2 |
| IMPHY011521 | 2-Undecanone | -4.4 | -5.4 | -4.7 | -4.2 | -4.5 | -4.2 | -3.8 | -3.8 | -3.5 | -4.1 | -3.2 | -4.2 |
| IMPHY001516 | Decane | -5 | -4.6 | -4.6 | -4 | -4 | -3.8 | -3.8 | -3.7 | -3.9 | -3.8 | -3.5 | -4.1 |
| IMPHY007202 | Nonane | -4.9 | -4.6 | -4.6 | -4.2 | -3.7 | -3.9 | -3.7 | -3.9 | -3.6 | -3.6 | -3.3 | -4 |
